# General Synthesis of 2-Substituted Benzoxazoles Based on Tf_2_O-Promoted Electrophilic Activation of Tertiary Amides

**DOI:** 10.3390/molecules30071510

**Published:** 2025-03-28

**Authors:** Hongchen Li, Xingyong Wang, Fujun Zhao, Lu Wang, Songbao Fu

**Affiliations:** 1 CNOOC Institute of Chemicals & Advanced Materials, Beijing 102209, China; wangxy130@cnooc.com.cn (X.W.); zhaofj@cnooc.com.cn (F.Z.); wanglu30@cnooc.com.cn (L.W.); 2 Department of Chemistry, Tsinghua University, Beijing 100084, China

**Keywords:** benzoxazoles, electrophilic activation, triflic anhydride, tertiary amides

## Abstract

We report a method for the synthesis of 2-substituted benzoxazoles from tertiary amides and 2-aminophenols in the presence of triflic anhydride (Tf_2_O) and 2-Fluoropyridine (2-F-Pyr). The cascade reaction involves the activation of the amide carbonyl group by Tf_2_O, nucleophilic addition, intramolecular cyclization, and elimination. Furthermore, we explore the scope of this method by varying both the amide and 2-aminophenol substrates, highlighting its versatility in the synthesis of a wide range of functionalized benzoxazole derivatives.

## 1. Introduction

**Figure 1:**
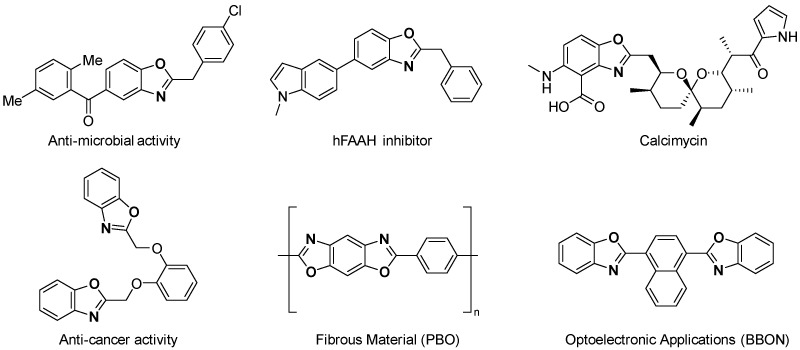
Representative 2-substituted benzoxazole derivatives and applications.

The 2-substituted benzoxazoles are an important class of heterocyclic compounds [1,2,3,4]. These compounds have attracted significant attention for their promising applications in medicinal chemistry [5,6,7,8,9,10,11,12,13,14,15,16,17], materials science [18], and organic electronics [19]. In particular, 2-substituted benzoxazoles exhibit a range of pharmacological activities, including anti-microbial [6], anti-cancer [8], anti-inflammatory [16], and anticonvulsant effects [17], which makes them valuable scaffolds for the development of new therapeutic agents (Figure 1).

**Scheme 1:**
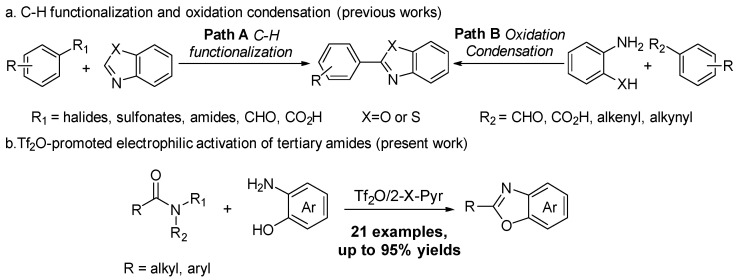
(**a**) C-H functionalization and oxidation condensation; (**b**) Tf_2_O-promoted electrophilic activation of tertiary amides.

Given the importance of 2-substituted benzoxazoles, numerous synthetic strategies have been developed to construct these valuable heterocyclic frameworks. Currently, the synthesis of 2-substituted benzoxazoles can be broadly categorized into two main approaches (Scheme 1a) [20,21,22]. The first approach involves the direct functionalization of the C-H bond at the C2 position, which allows the direct functionalization of the benzoxazole core (Scheme 1a, Path A) [23,24,25,26]. This strategy has attracted considerable attention due to its high atom economy and the ability to introduce a wide variety of functional groups without the need for pre-functionalized substrates. However, many of these transformations still require strong oxidants, high temperatures or very low temperatures, and special catalysts, which can limit their practical application. In addition, the use of transition metal catalysts poses challenges in pharmaceutical and industrial applications due to the presence of metal residues that require additional purification steps.

In view of the challenges associated with direct C-H functionalization at the C2 position of benzoxazoles, an alternative and widely used strategy for the synthesis of 2-substituted benzoxazoles is the cyclization of suitably functionalized precursors [27,28,29,30,31,32,33]. This method makes it possible to construct the benzoxazole core straightforwardly and to introduce the desired substituent at the C2 position at the same time (Scheme 1a, Path B). One of the most common methods is the cyclization of 2-aminophenols with aryl or alkyl precursors, such as benzoic acids [27], aryl methyl ketones [28,29], alkynes [30], and styrene derivatives [31,32,33]. These cyclization strategies provide a more general and versatile route to 2-substituted benzoxazoles. They often show high selectivity and better substrate tolerance compared to C-H activation methods.

In recent years, amide transformation has gained significant attention as a starting material for the synthesis of heterocyclic compounds [34,35,36,37,38]. This is due to the availability and versatility of amide substrates. However, efficient activation of the amide functional group is the key challenge in using amides for heterocycle synthesis. Amides are generally considered to be relatively inert compared to other carbonyl-containing compounds, such as esters or acids. This is mainly because the amide is affected by the increased stability of the electrophilic carbonyl carbon, resulting in much lower reactivity with nucleophilic reagents [34]. Under mild conditions, this makes direct reactions difficult. Recent advances in electrophilic activation strategies, including using strong acids like Tf_2_O, have proven to be very effective [39,40,41,42,43,44]. Herein, we report a method that explores the use of Tf_2_O-promoted electrophilic activation to synthesize 2-substituted benzoxazoles, highlighting its potential as a more selective and efficient activation strategy compared to traditional methods (Scheme 1b).

The ability of Tf_2_O to activate amides through the formation of highly reactive intermediates opens up new ways of functionalizing both amides and 2-aminophenols. This provides access to a wide range of substituted benzoxazoles.

## 2. Results and Discussion

### 2.1. Synthesis

#### 2.1.1. Optimization of Reaction Conditions

As part of our continuing research into amide activation to construct heterocyclic compounds, we hypothesized that amides could be directly cyclized as starting materials to synthesize 2-substituted benzoxazoles. As expected, we successfully obtained the target product 2-benzylbenzo[d]oxazole (**3aa**) by the reaction of 1-morpholino-2-phenylethan-1-one (**1a**) and 2-aminophenol (**2a**) in the presence of Tf_2_O (Table 1, entry 1). Based on these preliminary results, we investigated different reaction conditions to optimize the process. First, the effect of different bases on the reaction was tested. The results are summarized in Table 1. The yield of the product was significantly increased by adding pyridine-based organic bases to the reaction (Table 1, entries 2-9). However, if the pyridine ring contains strong electron-absorbing functional groups (such as –SO_3_H and –NO_2_), the effect of the pyridine base is less obvious and the yield is reduced (Table 1, entries 8-9). When inorganic bases, such as CsF and K_2_CO_3_, were used, the reaction showed little improvement (Table 1, entries 10-11). The reaction yield was significantly increased by increasing the amount of 2-Fluoropyridine (Table 1, entries 12–14).

We then further optimized the reaction by exploring the impact of different conditions. A variety of solvents were tested, and DCM gave the best results (Table 2, entries 1–4). Reducing the reaction temperature had little effect on the yield, with the highest yield being obtained at room temperature (Table 2, entries 5–6). There is no significant increase in yield by further extension of the reaction time above one hour (Table 2, entries 7–8). By adjusting the ratio of the two starting materials, the highest yield, which was 95%, was obtained when the ratio of **1a**/**2a** was 1.1/1 (Table 2, entries 9–11).

#### 2.1.2. The Effect of Different Amide N-Leaving Groups on the Reaction

**Scheme 2:**
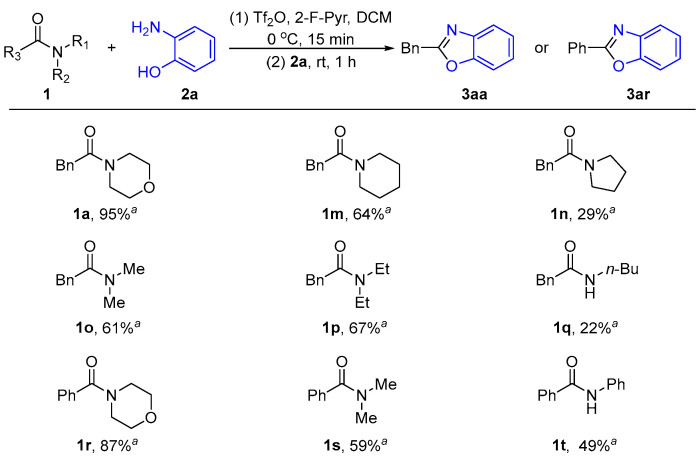
Scope of N-leaving groups ^a^. Reactions were performed with 0.55 mmol of **1**, 0.5 mmol of **2a**, 1.0 mmol of 2-F-Pyr, and 0.6 mmol of Tf_2_O in DCM (1.0 mL) at room temperature for 1 h. In each case, the yield refers to the effective amount of isolated compound.

After establishing the optimal reaction conditions, we tested the effect of different amide N-leaving groups on the reaction, focusing primarily on the influence of various alkyl and aryl groups (R_1_ and R_2_) on the nitrogen atom in amides (Scheme 2). At first, we examined the impact of different functional groups (R_1_ and R_2_) on the nitrogen atom of benzylamides. Unfortunately, the reaction only worked best when the nitrogen atom contained the morpholine functional group (Scheme 2, **1a**). In cases where other functional groups (R_1_ and R_2_) were present on the nitrogen, the yields ranged from 22% to 67% (Scheme 2, **1m**–**1q**). In order to verify these findings, we obtained similar results by substituting the benzamides for benzylamides (Scheme 2, **1r**–**1t**). This variation in yield is probably due to the polar oxygen atom in morpholine.

#### 2.1.3. Substrates’ Scope Under Optimized Conditions

**Scheme 3:**
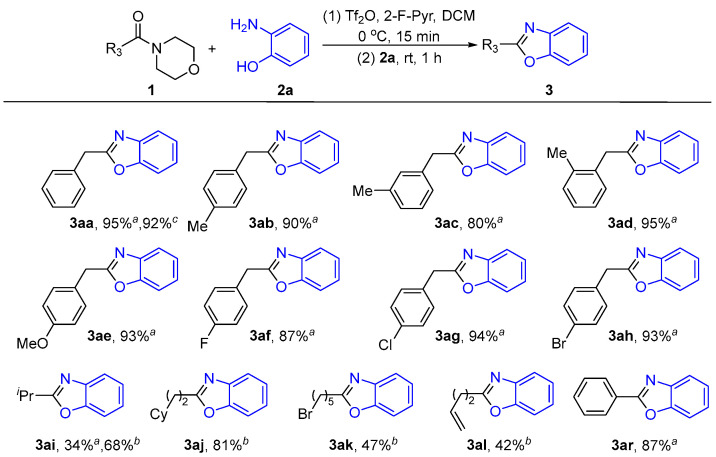
Substrate scopes of amide compounds ^a–c^. ^a^ Reactions were performed with 0.55 mmol of **1**, 0.5 mmol of **2a**, 1.0 mmol of 2-F-Pyr, and 0.6 mmol of Tf_2_O in DCM (1.0 mL) at room temperature for 1 h. ^b^ stirred at 90 °C for 1 h. ^c^ gram scale: Reaction was performed with 22 mmol of **1a**, 20 mmol of **2a**, 40 mmol of 2-F-Pyr, and 24 mmol of Tf_2_O in DCM (50 mL) at room temperature for 1 h. In each case, the yield refers to the effective amount of isolated compound.

Under the optimal reaction conditions, we explored the scope of amide compounds in the reaction. As shown in Scheme 3, amides (**1**) with different alkyl groups (R_3_) have been reacted with 2-aminophenol (**2a**) to give the corresponding products (**3**). The yields ranged from moderate to excellent. When the benzyl group contains various functional groups, such as methyl, methoxy, or halogen groups, the reaction generally proceeds with excellent results (**3aa**–**3ah**). No significant steric or electronic effects were observed over the above substrates. It is noteworthy that other alkyl amides, such as isopropyl and cycloalkyl amides, also reacted efficiently (**3ai**–**3aj**). Excitingly, the alkylamides containing halogens and alkenes also led to the desired products (**3ak**–**3al**). This significantly extends the scope of 2-alkylbenzoxazole synthesis. The same method can also be used to prepare 2-arylbenzooxazole with a yield of 87% (**3ar**). This can be achieved by using benzamide as the starting material. To demonstrate the potential usefulness of the method, we carried out the reaction on a gram scale and obtained the product **3aa** in a yield comparable to that of the small-scale reaction.

**Scheme 4:**
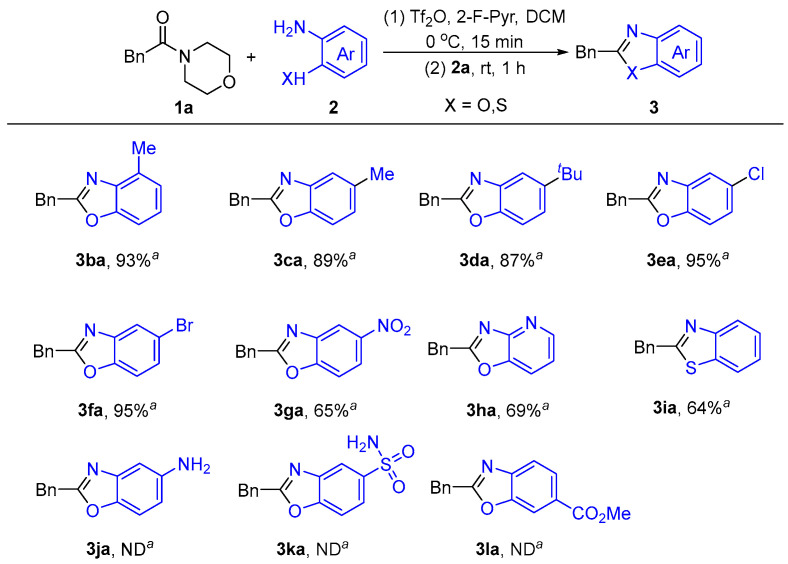
Substrate scopes of nucleophiles ^a^. ^a^ Reactions were performed with 0.55 mmol of **1a**, 0.5 mmol of **2**, 1.0 mmol of 2-F-Pyr, and 0.6 mmol of Tf_2_O in DCM (1.0 mL) at room temperature for 1 h. In each case, the yield refers to the effective amount of isolated compound.

We also explored the effects of different substituents on the nucleophiles in the reaction (Scheme 4). Alkyl-substituted 2-aminophenols, such as methyl and tert-butyl 2-aminophenols, can be used to give good yields of 2-benzylbenzoxazoles (**3ba**–**3da**). If the 2-aminophenols contains halogen substituents, such as chlorine or bromine, the target product is also obtained in excellent yields (**3ea**–**3fa**). The nitro-substituted aminophenol compound shows an impressive tolerance in this reaction and the desired products are produced with an ideal yield of 65% (**3ga**). Despite a moderate yield, an aromatic pyridine substrate also gives the target product (**3ha**). In particular, replacing the aminophenol group with *o*-thiosamine leads to the formation of 2-alkylbenzothiazole (**3ia**). This greatly expands the synthetic scope of benzoheterocyclic compounds. We also tested several special substituents of 2-aminophenol, such as amino groups, sulfonamide groups, or ester groups. The experimental results found that under the action of Tf_2_O, the amino and amide groups form amidine, and the esters can consume anhydrides, which makes it difficult to continue the reaction. (**3ja**–**3la**).

### 2.2. Proposed Mechanism

**Scheme 5:**
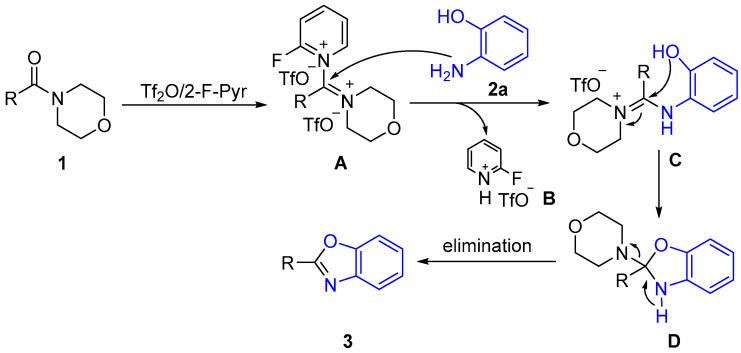
Proposed Mechanism.

Based on the experimental results and previous studies [26,45], we proposed a possible reaction mechanism for the synthesis of 2-substituted benzoxazole (Scheme 5). First of all, the amide (**1**) reacts with Tf_2_O and 2-Fluoropyridine to form an intermediate amidinium salt **A**. Then, the amino nitrogen of the 2-aminophenol compound (**2a**) acts as a nucleophile and attacks the carbon of the amidinium to form the intermediate **C**. In this step, one molecule of **B** is released by the addition/elimination reaction. Next, an intramolecular cyclization reaction is carried out to produce the intermediate **D**. Finally, the target product **3** is obtained by elimination reaction.

## 3. Materials and Methods

### 3.1. Materials and General Methods

The reaction vessel uses a thick-walled pressure bottle (upper pressure limit: 6 bar; reactor volume: 15 mL or 350 mL). All reagents used in experiment were obtained from commercial sources and used without further purification. Chromatographic purification of products was accomplished using silica gel (200–300 mesh). All melting points were determined on a Yanaco MP-500 micro melting point apparatus (Yanaco, Kyoto, Japan) and were uncorrected. All spectra of ^1^H NMR (400 MHz) and ^13^C NMR (100 MHz) were recorded on a JEOL JNM-ECA 400 spectrometer (JEOL, Tokyo, Japan) in CDCl_3_. NMR spectrum data processing using Delta NMR processing and control software (5.3.1 Windows). TMS was used as an internal reference and J values are given in Hz. PE is petroleum ether (60–90 °C).

### 3.2. General Procedure for the Preparation of 2-Substituted Benzoxazoles 3

2-Fluoropyridine (1 mmol, 97 mg) was added to a solution of amide **1a** (0.55 mmol, 113 mg) in 1 mL DCM. The mixture was cooled to 0 °C and Tf_2_O (0.6 mmol, 170 mg) was added dropwise and stirred for 15 minutes. Then, 2-aminophenol **2a** (0.5 mmol, 54.5 mg) was added and the reaction was stirred for 1 hour at room temperature. The reaction was quenched with 0.5 mL Et_3_N. The solvent was evaporated and the residue was purified by chromatography (silica gel, PE: EtOAc = 20:1) to give the desired product 2-benzylbenzo[*d*]oxazole (**3aa**, 99 mg, 95%) as a yellowish oil.

The products **3ab**–**3ar** and **3ba**–**ia** were prepared by similar procedure.

### 3.3. Analytical Data of Compound ***3aa**–**3ar*** and ***3ba**–**ia***

*2-Benzylbenzo[d]oxazole* (**3aa**) [46]. The solvent was evaporated and the residue was purified by chromatography (silica gel, PE: EtOAc = 20:1) to give the desired product **3aa** (99 mg, 95%) as a yellowish oil. ^1^H NMR (400 MHz, CDCl_3_) δ 7.72–7.64 (m, 1H), 7.45–7.40 (m, 1H), 7.39–7.28 (m, 4H), 7.28–7.21 (m, 3H), 4.24 (s, 2H). ^13^C NMR (100 MHz, CDCl_3_) δ 165.1, 150.9, 141.3, 134.7, 128.9 (2C), 128.7 (2C), 127.2, 124.6, 124.1, 119.7, 110.3, 35.2.

*2-(4-Methylbenzyl)benzo[d]oxazole* (**3ab**) [46]. The solvent was evaporated and the residue was purified by chromatography (silica gel, PE:EtOAc = 20:1) to give the desired product **3ab** (100 mg, 90%) as a yellowish oil. ^1^H NMR (400 MHz, CDCl_3_) δ 7.70–7.65 (m, 1H), 7.47–7.41 (m, 1H), 7.31–7.23 (m, 4H), 7.17–7.11 (m, 2H), 4.22 (s, 2H), 2.32 (s, 3H). ^13^C NMR (100 MHz, CDCl_3_) δ 165.4, 151.0, 141.3, 136.9, 131.7, 129.5 (2C), 128.8 (2C), 124.6, 124.1, 119.8, 110.4, 34.8, 21.0.

*2-(3-Methylbenzyl)benzo[d]oxazole* (**3ac**) [46]. The solvent was evaporated and the residue was purified by chromatography (silica gel, PE:EtOAc = 20:1) to give the desired product **3ac** (89 mg, 80%) as a yellowish oil. ^1^H NMR (400 MHz, CDCl_3_) δ 7.72–7.65 (m, 1H), 7.47–7.42 (m, 1H), 7.31–7.14 (m, 5H), 7.10–7.05 (m, 1H), 4.22 (s, 2H), 2.33 (s, 3H).^13^C NMR (100 MHz, CDCl_3_) δ 165.3, 151.0, 141.3, 138.5, 134.6, 129.7, 128.7, 128.0, 126.0, 124.6, 124.1, 119.8, 110.4, 35.2, 21.3.

*2-(2-Methylbenzyl)benzo[d]oxazole* (**3ad**) [46]. The solvent was evaporated and the residue was purified by chromatography (silica gel, PE:EtOAc = 20:1) to give the desired product **3ad** (106 mg, 95%) as a yellow oil. ^1^H NMR (400 MHz, CDCl_3_) δ 7.70–7.64 (m, 1H), 7.45–7.39 (m, 1H), 7.31–7.22 (m, 3H), 7.22–7.14 (m, 3H), 4.24 (s, 2H), 2.38 (s, 3H). ^13^C NMR (100 MHz, CDCl_3_) δ 165.0, 150.9, 141.3, 136.7, 133.2, 130.5, 129.9, 127.5, 126.3, 124.5, 124.0, 119.7, 110.3, 33.0, 19.6.

*2-(4-Methoxybenzyl)benzo[d]oxazole* (**3ae**) [46]. The solvent was evaporated and the residue was purified by chromatography (silica gel, PE:EtOAc = 20:1) to give the desired product **3ae** (111 mg, 93%) as a yellowish oil. ^1^H NMR (400 MHz, CDCl_3_) δ 7.70–7.65 (m, 1H), 7.47–7.42 (m, 1H), 7.32–7.23 (m, 4H), 6.91–6.84 (m, 2H), 4.20 (s, 2H), 3.77 (s, 3H). ^13^C NMR (100 MHz, CDCl_3_) δ 165.5, 158.8, 151.0, 141.3, 130.0 (2C), 126.7, 124.6, 124.1, 119.7, 114.2 (2C), 110.4, 55.2, 34.4.

*2-(4-Fluorobenzyl)benzo[d]oxazole* (**3af**) [47]. The solvent was evaporated and the residue was purified by chromatography (silica gel, PE:EtOAc = 20:1) to give the desired product **3af** (99 mg, 87%) as a yellowish oil. ^1^H NMR (400 MHz, CDCl_3_) δ 7.71–7.65 (m, 1H), 7.48–7.42 (m, 1H), 7.37–7.24 (m, 4H), 7.06–6.98 (m, 2H), 4.23 (s, 2H). ^13^C NMR (100 MHz, CDCl_3_) δ 164.9, 162.1 (d, *J* = 244.1 Hz), 151.0, 141.3, 130.5 (2C, d, *J* = 7.6 Hz), 130.4 (d, *J* = 3.8 Hz), 124.8, 124.2, 119.8, 115.7 (2C, d, *J* = 21.9 Hz), 110.4, 34.4.

*2-(4-Chlorobenzyl)benzo[d]oxazole* (**3ag**) [25]. The solvent was evaporated and the residue was purified by chromatography (silica gel, PE:EtOAc = 20:1) to give the desired product **3ag** (114 mg, 94%) as a white oil. ^1^H NMR (400 MHz, CDCl_3_) δ 7.71–7.65 (m, 1H), 7.48–7.42 (m, 1H), 7.33–7.24 (m, 6H), 4.22 (s, 2H). ^13^C NMR (100 MHz, CDCl_3_) δ 164.6, 151.0, 141.2, 133.3, 133.1, 130.3 (2C), 128.9 (2C), 124.8, 124.2, 119.8, 110.4, 34.5.

*2-(4-Bromobenzyl)benzo[d]oxazole* (**3ah**) [29]. The solvent was evaporated and the residue was purified by chromatography (silica gel, PE:EtOAc = 20:1) to give the desired product **3ah** (133 mg, 93%) as a white solid, mp 95−98 °C. ^1^H NMR (400 MHz, CDCl_3_) δ 7.71–7.65 (m, 1H), 7.48–7.42 (m, 3H), 7.32–7.21 (m, 4H), 4.20 (s, 2H). ^13^C NMR (100 MHz, CDCl_3_) δ 164.4, 151.0, 141.2, 133.6, 131.9 (2C), 130.7 (2C), 124.8, 124.2, 121.3, 119.8, 110.4, 34.6.

*2-Isopropylbenzo[d]oxazole* (**3ai**) [48]. The solvent was evaporated and the residue was purified by chromatography (silica gel, PE:EtOAc = 30:1) to give the desired product **3ai** (55 mg, 68%, stirred in DCE at 90 °C for 1 h) as a brown oil. ^1^H NMR (400 MHz, CDCl_3_) δ 7.71–7.66 (m, 1H), 7.51 7.45 (m, 1H), 7.32–7.25 (m, 2H), 3.25 (heptuple, *J* = 6.9 Hz, 1H), 1.46 (d, *J* = 6.9 Hz, 6H). ^13^C NMR (100 MHz, CDCl_3_) δ 171.3, 150.7, 141.2, 124.4, 124.0, 120.0, 110.2, 28.9, 20.3 (2C).

*2-(2-Cyclohexylethyl)benzo[d]oxazole* (**3aj**) [26]. The solvent was evaporated and the residue was purified by chromatography (silica gel, PE:EtOAc = 30:1) to give the desired product **3aj** (93 mg, 81%, stirred in DCE at 90 °C for 1 h) as a brown oil. ^1^H NMR (400 MHz, CDCl_3_) δ 7.69–7.63 (m, 1H), 7.50–7.44 (m, 1H), 7.32–7.24 (m, 2H), 2.98–2.89 (m, 2H), 1.82–1.61 (m, 7H), 1.39–1.10 (m, 4H), 1.05–0.88 (m, 2H). ^13^C NMR (100 MHz, CDCl_3_) δ 167.6, 150.7, 141.4, 124.3, 123.9, 119.4, 110.2, 37.1, 34.1, 32.9 (2C), 26.5, 26.1(3C).

*2-(5-Bromopentyl)benzo[d]oxazole* (**3ak**) [49]. The solvent was evaporated and the residue was purified by chromatography (silica gel, PE:EtOAc = 30:1) to give the desired product **3ak** (63 mg, 47%, stirred in DCE at 90 °C for 1 h) as a white solid, mp 81−83 °C. ^1^H NMR (400 MHz, CDCl_3_) δ 7.70–7.64 (m, 1H), 7.51–7.45 (m, 1H), 7.33–7.26 (m, 2H), 3.42 (t, *J* = 6.6 Hz, 2H), 2.95 (t, *J* = 7.6 Hz, 2H), 1.98–1.88 (m, 4H), 1.64–1.54 (m, 2H). ^13^C NMR (100 MHz, CDCl_3_) δ 166.7, 150.7, 141.3, 124. 5, 124.1, 119.5, 110.2, 33.3, 32.3, 28.4, 27.6, 25.8.

*2-(But-3-en-1-yl)benzo[d]oxazole* (**3al**) [50]. The solvent was evaporated and the residue was purified by chromatography (silica gel, PE:EtOAc = 30:1) to give the desired product **3al** (36 mg, 42%, stirred in DCE at 90 °C for 1 h) as a colorless oil. ^1^H NMR (400 MHz, CDCl_3_) δ 7.70–7.64 (m, 1H), 7.51–7. 45 (m, 1H), 7.32–7.25 (m, 2H), 6.00–5.82 (m, 1H), 5.17–5.08 (m, 1H), 5.08–5.00 (m, 1H), 3.03 (t, *J* = 7.6 Hz, 2H), 2.71–2.59 (m, 2H). ^13^C NMR (100 MHz, CDCl_3_) δ 166.4, 150.8, 141.3, 136.2, 124.4, 124.0, 119.5, 116.0, 110.2, 30.5, 28.1.

*2-Phenylbenzo[d]oxazole* (**3ar**) [26]. The solvent was evaporated and the residue was purified by chromatography (silica gel, PE:EtOAc = 15:1) to give the desired product **3ar** (85 mg, 87%) as a white solid, mp 95−97 °C (lit. 97–98 °C). ^1^H NMR (400 MHz, CDCl_3_) δ 8.27–8.24 (m, 2H), 7.79–7.76 (m, 1H), 7.59–7.49 (m, 4H), 7.37–7.31 (m, 2H). ^13^C NMR (100 MHz, CDCl_3_) δ 162.9, 150.7, 142.1, 131.5, 128.9 (2C), 127.6 (2C), 127.1, 125.1, 124.5, 119.9, 110.5.

*2-Benzyl-4-methylbenzo[d]oxazole* (**3ba**) [33]. The solvent was evaporated and the residue was purified by chromatography (silica gel, PE:EtOAc = 20:1) to give the desired product **3ba** (104 mg, 93%) as a colorless oil. ^1^H NMR (400 MHz, CDCl_3_) δ 7.41–7.29 (m, 4H), 7.29–7.22 (m, 2H), 7.20–7.12 (m, 1H), 7.11–7.05 (m, 1H), 4.27 (s, 2H), 2.61 (s, 3H).^13^C NMR (100 MHz, CDCl_3_) δ 164.2, 150.8, 140.5, 135.0, 130.1, 128.9 (2C), 128.7 (2C), 127.2, 124.7, 124.3, 107.7, 35.3, 16.5.

*2-Benzyl-5-methylbenzo[d]oxazole* (**3ca**) [33]. The solvent was evaporated and the residue was purified by chromatography (silica gel, PE:EtOAc = 20:1) to give the desired product **3ca** (99 mg, 89%) as a yellowish oil. ^1^H NMR (400 MHz, CDCl_3_) δ 7.46 (s, 1H), 7.39–7.29 (m, 5H), 7.29–7.23 (m, 1H), 7.10–7.05 (m, 1H), 4.24 (s, 2H), 2.43 (s, 3H). ^13^C NMR (100 MHz, CDCl_3_) δ 165.2, 149.3, 141.5, 134.9, 133.9, 128.9 (2C), 128.7 (2C), 127.2, 125.7, 119.7, 109.8, 35.3, 21.4.

*2-Benzyl-5-(tert-butyl)benzo[d]oxazole* (**3da**) [46]. The solvent was evaporated and the residue was purified by chromatography (silica gel, PE:EtOAc = 20:1) to give the desired product **3da** (115 mg, 87%) as a yellowish oil. ^1^H NMR (400 MHz, CDCl_3_) δ 7.72–7.69 (m, 1H), 7.40–7.29 (m, 6H), 7.29–7.22 (m, 1H), 4.24 (s, 2H), 1.36 (s, 9H). ^13^C NMR (100 MHz, CDCl_3_) δ 165.3, 149.0, 147.7, 141.2, 134.9, 128.9 (2C), 128.8 (2C), 127.2, 122.3, 116.3, 109.5, 35.3, 34.8, 31.7 (3C).

*2-Benzyl-5-chlorobenzo[d]oxazole* (**3ea**) [25]. The solvent was evaporated and the residue was purified by chromatography (silica gel, PE:EtOAc = 20:1) to give the desired product **3ea** (116 mg, 95%) as a yellowish oil. ^1^H NMR (400 MHz, CDCl_3_) δ 7.66–7.65 (m, 1H), 7.39–7.31 (m, 5H), 7.31–7.23 (m, 2H), 4.25 (s, 2H). ^13^C NMR (100 MHz, CDCl_3_) δ 166.6, 149.6, 142.4, 134.3, 129.6, 129.0 (2C), 128.9 (2C), 127.4, 125.0, 119.8, 111.1, 35.2.

*2-Benzyl-5-bromobenzo[d]oxazole* (**3fa**) [33]. The solvent was evaporated and the residue was purified by chromatography (silica gel, PE:EtOAc = 20:1) to give the desired product **3fa** (137 mg, 95%) as a yellowish oil. ^1^H NMR (400 MHz, CDCl_3_) δ 7.82–7.80 (m, 1H), 7.42–7.23 (m, 7H), 4.25 (s, 2H). ^13^C NMR (100 MHz, CDCl_3_) δ 166.4, 150.0, 142.9, 134.3, 128.9 (2C), 128.8 (2C), 127.7, 127.4, 122.8, 116.9, 111.7, 35.2.

*2-Benzyl-5-nitrobenzo[d]oxazole* (**3ga**) [51]. The solvent was evaporated and the residue was purified by chromatography (silica gel, PE:EtOAc = 15:1) to give the desired product **3ga** (83 mg, 65%) as a yellow oil. ^1^H NMR (400 MHz, CDCl_3_) δ 8.58–8.56 (m, 1H), 8.29–8.25 (m, 1H), 7.57–7.55 (m, 1H), 7.42–7.27 (m, 5H), 4.32 (s, 2H). ^13^C NMR (100 MHz, CDCl_3_) δ 168.4, 154.5, 145.1, 141.7, 133.7, 129.0 (2C), 129.0 (2C), 127.7, 120.9, 116.2, 110.6, 35.2. 

*2-Benzyloxazolo[4,5-b]pyridine* (**3ha**) [52]. The solvent was evaporated and the residue was purified by chromatography (silica gel, PE:EtOAc = 8:1) to give the desired product **3ha** (73 mg, 69%) as a colorless solid, mp 93−95 °C (lit. 95–97 °C). ^1^H NMR (400 MHz, CDCl_3_) δ 8.55–8.50 (m, 1H), 7.77–7.69 (m, 1H), 7.43–7.38 (m, 2H), 7.38–7.32 (m, 2H), 7.31–7.26 (m, 1H), 7.26–7.20 (m, 1H), 4.33 (s, 2H). ^13^C NMR (100 MHz, CDCl_3_) δ 168.2, 155.7, 146.2, 143.1, 134.0, 129.0 (2C), 128.8 (2C), 127.4, 119.8, 118.0, 35.5. 

*2-Benzylbenzo[d]thiazole* (**3ia**) [53]. The solvent was evaporated and the residue was purified by chromatography (silica gel, PE:EtOAc = 30:1) to give the desired product **3ia** (72 mg, 64%) as a yellowish oil. ^1^H NMR (400 MHz, CDCl_3_) δ 7.99 (d, *J* = 8.0 Hz, 1H), 7.77 (d, *J* = 8.4 Hz, 1H), 7.47–7.40 (m, 1H), 7.38–7.25 (m, 6H), 4.43 (s, 2H). ^13^C NMR (100 MHz, CDCl_3_) δ 171.1, 153.2, 137.1, 135.6, 129.1 (2C), 128.8 (2C), 127.3, 125.9, 124.8, 122.7, 121.5, 40.6.

## 4. Conclusions

In conclusion, we have developed a new method for the synthesis of 2-substituted benzoxazoles from tertiary amides and 2-aminophenols in the presence of Tf_2_O and 2-Fluoropyridine. The 2-substituted benzoxazoles have been prepared by cascade reactions, including Tf_2_O-activated amides, nucleophilic addition, intramolecular cyclization, and elimination. The method was simple, mild, and effective for the synthesis of 2-substituted benzoxazoles and can be extended to the synthesis of 2-substituted benzothiazoles. Given its versatility and ease of use, we anticipate that this method will have broad applications in organic synthesis.

## Figures and Tables

**Table 1:** Testing the effect of different bases on the reaction ^a^.

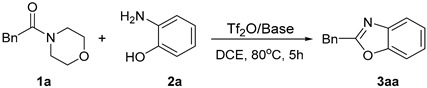			
Entry	Base	Tf_2_O/Base (by Equiv)	Yield of 3aa ^b^ (%)
1	/	1.2/0	41%
2	2-F-Pyr	1.2/1.5	87%
3	2-Cl-Pyr	1.2/1.5	85%
4	2,6-Lutidine	1.2/1.5	68%
5	DMAP	1.2/1.5	46%
6	Pyridine	1.2/1.5	65%
7	3-F-Pyr	1.2/1.5	80%
8	2-Pyridinesulfonic acid	1.2/1.5	30%
9	2-Nitropyridine	1.2/1.5	32%
10	CsF	1.2/1.5	31%
11	K_2_CO_3_	1.2/1.5	20%
12	2-F-Pyr	1.2/1.2	81%
13	2-F-Pyr	1.2/2.0	89%
14	2-F-Pyr	1.2/2.5	88%

^a^ Reaction conditions: Tf_2_O was added to a stirred solution of **1a** (0.6 mmol) and base in DCE (1 mL) at 0 °C. After 15 min, **2a** (0.5 mmol) was added and stirred at 80 °C for 5 h. ^b^ Isolated yields.

**Table 2:** Optimization of reaction conditions ^a^.

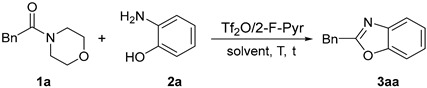					
Entry	1a/2a (by Equiv)	T (°C)	t (h)	Solvent	Yield of 3aa ^b^ (%)
1	1.2/1	80	5	DCM	93
2	1.2/1	80	5	CHCl_3_	89
3	1.2/1	80	5	CH_3_CN	63
4	1.2/1	80	5	Toluene	56
5	1.2/1	50	5	DCM	93
6	1.2/1	25	5	DCM	94
7	1.2/1	25	1	DCM	94
8	1.2/1	25	0.5	DCM	86
9	1/1.2	25	1	DCM	75
10	1/1	25	1	DCM	87
11	1.1/1	25	1	DCM	95

^a^ Reaction conditions: Tf_2_O was added to a stirred solution of **1a** (0.6 mmol) and base in DCE (1 mL) at 0 °C. After 15 min, **2a** (0.5 mmol) was added and stirred at 80 °C for 5 h. ^b^ Isolated yields.

## Data Availability

The data are contained within the article and Supplementary Materials.

## Supplementary Materials

The following supporting information can be downloaded at https://www.mdpi.com/article/10.3390/molecules30071510/s1: The structures of the substrates, ^1^H NMR and ^13^C NMR spectra of the products **3aa**–**3ar** and **3ba**–**3ia**.

## References

[1] Singh S., Veeraswamy G., Bhattarai D., Goo J.-I., Lee K., Choi Y. (2015). Recent Advances in the Development of Pharmacologically Active Compounds that Contain a Benzoxazole Scaffold. Asian J. Org. Chem..

[2] Arulmurugan S., Kavitha H.P., Vennila J.P. (2021). Review on the Synthetic Methods of Biologically Potent Benzoxazole Derivatives. MINI-Rev. Org. Chem..

[3] Wong X.K., Yeong K.Y. (2021). A Patent Review on the Current Developments of Benzoxazoles in Drug Discovery. ChemMedChem.

[4] Di Martino S., De Rosa M. (2024). The Benzoxazole Heterocycle: A Comprehensive Review of the Most Recent Medicinal Chemistry Developments of Antiproliferative, Brain-Penetrant, and Anti-inflammatory Agents. Top. Curr. Chem..

[5] Arakawa K., Inamasu M., Matsumoto M., Okumura K., Yasuda K., Akatsuka H., Kawanami S., Watanabe A., Homma K., Saiga Y. (1997). Novel Benzoxazole 2,4-Thiazolidinediones as Potent Hypoglycemic Agents. Synthesis and Structure-Activity Relationships. Chem. Pharm. Bull..

[6] Tekiner-Gulbas B., Temiz-Arpaci O., Yildiz I., Altanlar N. (2007). Synthesis and in Vitro Antimicrobial Activity of New 2-[*p*-substituted-benzyl]-5-[substituted-carbonylamino]benzoxazoles. Eur. J. Med. Chem..

[7] Oksuzoglu E., Temiz-Arpaci O., Tekiner-Gulbas B., Eroglu H., Sen G., Alper S., Yildiz I., Diril N., Aki-Sener E., Yalcin I. (2008). A Study on the Genotoxic Activities of Some New Benzoxazoles. Med. Chem. Res..

[8] Jiang J., Tang X., Dou W., Zhang H., Liu W., Wang C., Zheng J. (2010). Synthesis and Characterization of the Ligand Based on Benzoxazole and its Transition Metal Complexes: DNA-binding and Antitumor Activity. J. Inorg. Biochem..

[9] Chancellor D.R., Davies K.E., De Moor O., Dorgan C.R., Johnson P.D., Lambert A.G., Lawrence D., Lecci C., Maillol C., Middleton P.J. (2011). Discovery of 2-Arylbenzoxazoles as Upregulators of Utrophin Production for the Treatment of Duchenne Muscular Dystrophy. Med. Chem..

[10] Sattar R., Mukhtar R., Atif M., Hasnain M., Irfan A. (2020). Synthetic Transformations and Biological Screening of Benzoxazole Derivatives: A Review. J. Heterocycl. Chem..

[11] Demmer C.S., Bunch L. (2015). Benzoxazoles and Oxazolopyridines in Medicinal Chemistry Studies. Eur. J. Med. Chem..

[12] Rubner R. (2004). Innovation via Photosensitive Polyimide and Poly(benzoxazole) Precursors—A Review by Inventor. J. Sci. Technol..

[13] Kuroyanagi J.I., Kanai K., Sugimoto Y., Horiuchi T., Achiwa I., Takeshita H., Kawakami K. (2010). 1,3-Benzoxazole-4-Carbonitrile as a Novel Antifungal Scaffold of β-1,6-glucan Synthesis Inhibitors. Bioorg. Med. Chem..

[14] Estiarte M.A., Johnson R.J., Kaub C.J., Gowlugari S., O’Mahony D.J.R., Nguyen M.T., Emerling D.E., Kelly M.G., Kincaid J., Vincent F. (2012). 2-Amino-5-arylbenzoxazole Derivatives as Potent Inhibitors of Fatty Acid Amide Hydrolase (FAAH). MedChemComm.

[15] Huang L., Zhang W., Zhang X., Yin L., Chen B., Song J. (2015). Synthesis and Pharmacological Evaluation of Piperidine (piperazine)-substituted Benzoxazole Derivatives as Multi-target Antipsychotics. Bioorg. Med. Chem. Lett..

[16] Sałaga M., Sobczak M., Fichna J. (2014). Inhibition of Fatty Acid Amide Hydrolase (FAAH) as a Novel Therapeutic Strategy in the Treatment of Pain and Inflammatory Diseases in the Gastrointestinal Tract. Eur. J. Pharm. Sci..

[17] Ugale V.G., Bari S.B. (2014). Quinazolines: New horizons in anticonvulsant therapy. Eur. J. Med. Chem..

[18] Holmes G.A., Rice K., Snyder C.R. (2006). Ballistic Fibers: A Review of the Thermal, Ultraviolet and Hydrolytic Stability of the Benzoxazole Ring Structure. J. Mater. Sci..

[19] Peng J., Liu Y., Yang J., Chen Z., Wang K., Li A. (2025). Multifunctional Elastic Benzoxazole Derivative Crystals for Advanced Optoelectronic Applications. Sci. China Mater..

[20] Rajasekhar S., Maiti B., Chanda K. (2017). A Decade Update on Benzoxazoles, a Privileged Scaffold in Synthetic Organic Chemistry. Synlett.

[21] Basak S., Dutta S., Maiti D. (2021). Accessing C2-Functionalized 1,3-(Benz)azoles through Transition Metal-Catalyzed C–H Activation. Chem.-Eur. J..

[22] El Alami A., El Maraghi A., Sdassi H. (2024). Review of Synthesis Process of Benzoxazole and Benzothiazole Derivatives. Syn. Commun..

[23] Mukai T., Hirano K., Satoh T., Miura M. (2010). Palladium-Catalyzed Direct Benzylation of Azoles with Benzyl Carbonates. Org. Lett..

[24] Zhao X., Wu G., Zhang Y., Wang J. (2011). Copper-Catalyzed Direct Benzylation or Allylation of 1,3-Azoles with N-Tosylhydrazones. J. Am. Chem. Soc..

[25] Xie P., Huang H., Xie Y., Guo S., Xia C. (2012). Palladium-Catalyzed Selective C-H Benzylation towards Functionalized Azoles with a Quaternary Carbon Center. Adv. Synth. Catal..

[26] Niu Z.J., Li L.H., Liu X.Y., Liang Y.M. (2019). Transition-Metal-Free Alkylation/Arylation of Benzoxazole via Tf_2_O-Activated-Amide. Adv. Synth. Catal..

[27] Kumar R., Selvam C., Kaur G., Chakraborti A.K. (2005). Microwave-Assisted Direct Synthesis of 2-Substituted Benzoxazoles from Carboxylic Acids under Catalyst and Solvent-Free Conditions. Synlett.

[28] Mayo M.S., Yu X., Zhou X., Feng X., Yamamoto Y., Bao M. (2014). Synthesis of Benzoxazoles from 2-Aminophenols and β-Diketones Using a Combined Catalyst of Brønsted Acid and Copper Iodide. J. Org. Chem..

[29] Nguyen T.B., Retailleau P. (2017). Elemental Sulfur-Promoted Oxidative Rearranging Coupling between o-Aminophenols and Ketones: A Synthesis of 2-Alkyl benzoxazoles under Mild Conditions. Org. Lett..

[30] Jiang J., Li G., Zhang F., Deng G.-J. (2018). Aniline ortho C−H Sulfuration/Cyclization with Elemental Sulfur for Efficient Synthesis of 2-Substituted Benzothiazoles under Metal-Free Conditions. Adv. Synth. Catal..

[31] Li Z., Dong J., Wang J., Yang D.-Y., Weng Z. (2019). Elemental Sulfur-promoted One-pot Synthesis of 2-(2,2,2-trifluoroethyl)benzoxazoles and their Derivatives. Chem. Commun..

[32] Zhang J., Hu L., Liu Y., Zhang Y., Chen X., Luo Y., Peng Y., Han S., Pan B. (2021). Elemental Sulfur-Promoted Benzoxazole/Benzothiazole Formation Using a C=C Double Bond as a One-Carbon Donator. J. Org. Chem..

[33] Chau T.K., Ho N.T., Ho T.H., Nguyen A.T., Nguyen K.D., Phan N.T.S., Le H.V., Nguyen T.T. (2024). Synthesis of 2-benzyl Benzoxazoles and Benzothiazoles via Elemental Sulfur Promoted Cyclization of Styrenes with 2-nitrophenols and N,N-dialkyl-3-nitroanilines. Org. Biomol. Chem..

[34] Kaiser D., Bauer A., Lemmerer M., Maulide N. (2018). Amide Activation: An Emerging Tool for Chemoselective Synthesis. Chem. Soc. Rev..

[35] Chaudhari M.B., Gnanaprakasam B. (2019). Recent Advances in the Metal-Catalyzed Activation of Amide Bonds. Chem.-Asian J..

[36] Feng M., Zhang H., Maulide N. (2022). Challenges and Breakthroughs in Selective Amide Activation. Angew. Chem. Int. Ed..

[37] Kumar V., Dhawan S., Bala R., Mohite S.B., Singh P., Karpoormath R. (2022). Cu-Catalysed Transamidation of Unactivated Aliphatic Amides. Org. Biomol. Chem..

[38] Kumar V., Dhawan S., Bala R., Girase P.S., Singh P., Karpoormath R. (2022). Metal-Free Direct Annulation of 2-Aminophenols and 2-Aminothiophenols with Unactivated Amides through Transamidation: Access to Polysubstituted Benzoxazole and Benzothiazole Derivatives. Tetrahedron.

[39] Kaiser D., Teskey C.J., Adler P., Maulide N. (2017). Chemoselective Intermolecular Cross-Enolate-Type Coupling of Amides. J. Am. Chem. Soc..

[40] Fan T., Wang A., Li J.Q., Ye J.L., Zheng X., Huang P.Q. (2018). Versatile One-Pot Synthesis of Polysubstituted Cyclopent-2-enimines from α,β-Unsaturated Amides: Imino-Nazarov Reaction. Angew. Chem. Int. Ed..

[41] Li J., Berger M., Zawodny W., Simaan M., Maulide N. (2019). A Chemoselective α-Oxytriflation Enables the Direct Asymmetric Arylation of Amides. Chem.

[42] Weng Y., Min L., Zeng X., Shan L., Wang X., Hu Y. (2020). General Synthesis of α-Alkyl Ynones from Morpholine Amides and 1-Copper(I) Alkynes Promoted by Triflic Anhydride. Org. Lett..

[43] Shan L., Li H., Zheng W., Wang X., Wang X., Hu Y. (2022). Tandem Synthesis of 2-Azaspiro [4.5]deca-1,6,9-trien-8-ones Based on Tf_2_O-Promoted Activation of N-(2-Propyn-1-yl) Amides. J. Org. Chem..

[44] Tang Z., Yao Z., Yu Y., Huang J., Ma X., Zhao X., Chang Z., Zhao D. (2024). Photoredox-Catalyzed [3+2] annulation of Aromatic Amides with Olefins via Iminium Intermediates. Angew. Chem. Int. Ed..

[45] Shan L., Li H., Zheng W., Wang X., Wang X., Hu Y. (2022). Mechanistic Insights into Tf_2_O-Promoted Electrophilic Activation of 2-Propynamides and a New Synthesis of 2,4-Disubstituted Quinolines. Org. Lett..

[46] Ji Y.Y., Zhang Y., Hu Y.Y., Shao L.X. (2015). N-heterocyclic Carbene-Pd(II)-1-methylimidazole Complex Catalyzed C–H Bond Benzylation of (Benzo) oxazoles with Benzyl Chlorides. Tetrahedron.

[47] Shang R., Yang Z.W., Wang Y., Zhang S.L., Liu L. (2010). Palladium-Catalyzed Decarboxylative Couplings of 2-(2-Azaaryl) Acetates with Aryl Halides and Triflates. J. Am. Chem. Soc..

[48] Evindar G., Batey R.A. (2006). Parallel Synthesis of a Library of Benzoxazoles and Benzothiazoles using Ligand-accelerated Copper-Catalyzed Cyclizations of ortho-Halobenzanilides. J. Org. Chem..

[49] Yi J., Lu X., Sun Y.Y., Xiao B., Liu L. (2013). Nickel-Catalyzed Sonogashira Reactions of Non-Activated Secondary Alkyl bromides and Iodides. Angew. Chem. Int. Ed..

[50] Bastug G., Eviolitte C., Marko I.E. (2012). Functionalized Orthoesters as Powerful Building Blocks for the Efficient Preparation of Heteroaromatic Bicycles. Org. Lett..

[51] Wynne G.M., Wren S.P., Johnson P.D., Price D., De Moor O., Nugent G., Tinsley J.M., Storer R., Mulvaney A., Pye R.J. (2007). Preparation of Benzimidazoles, Benzoxazoles, Benzothiazoles, Indoles and their Analogs for the Treatment of Muscular Dystrophy and Cachexia. Appl. WO.

[52] Shen T.Y., Clark R.L., Pessolano A.A., Witzel B.E., Lanza T.J. (1977). Anti-Inflammatory Oxazole (4,5-B) Pyridines.

[53] Huang Y., Yan D., Wang X., Zhou P., Wu W., Jiang H. (2018). Controllable Assembly of the Benzothiazole Framework using a C≡C Triple Bond as a One-Carbon Synthon. Chem. Commun..

