# Supplementary material for: General Synthesis of 2-Substituted Benzoxazoles Based on Tf2O-Promoted Electrophilic Activation of Tertiary Amides
Source: Molecules. 2025 Mar 28;30(7):1510. doi: 10.3390/molecules30071510 (PMC11990891; doi:10.3390/molecules30071510)
Supplement: Supplementary file 1 [file molecules-30-01510-s001.zip › molecules-3534476-supplementary.pdf]

Supporting Information

General Synthesis of 2-Substituted Benzoxazoles Based on Tf_2O-Promoted Electrophilic Activation of Tertiary Amides

Hongchen Li [1,2,*], Xingyong Wang [1], Fujun Zhao [1], Lu Wang [1] and Songbao Fu [1,*]

[1] CNOOC Institute of Chemicals & Advanced Materials, Beijing 102209, China
[2] Department of Chemistry, Tsinghua University, Beijing 100084, China
* Correspondence: lihc19@tsinghua.org.cn (H.L.); fushb@cnooc.com.cn (S.F.)

List

1. The structures of the substrates **1a-1t** and **2a-2l**.......................................S2

2. ^{1}H NMR and ^{13}C NMR spectra of the products **3aa-3ar and 3ba-3ia**..............S3

1. The structures of the substrates

The structures of substrates 1a-1t

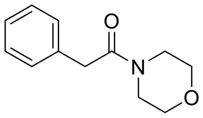
1a
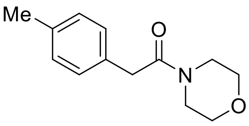
1b
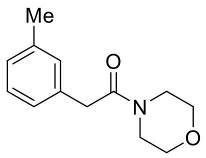
1c
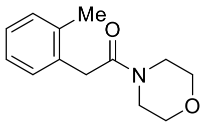
1d
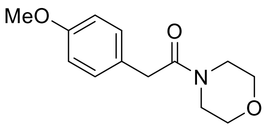
1e
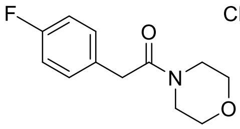
1f
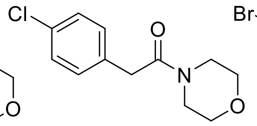
1g
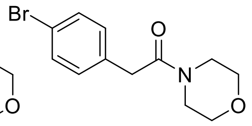
1h
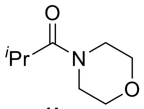
1i
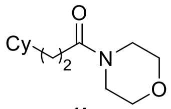
1j
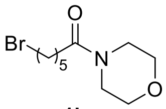
1k
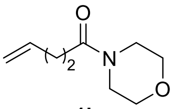
1l
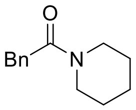
1m
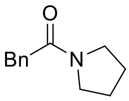
1n
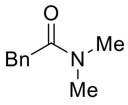
1o
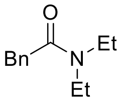
1p
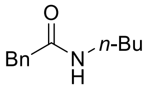
1q
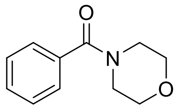
1r
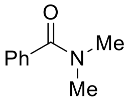
1s
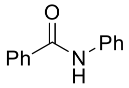
1t

The structures of substrates 2a-2l

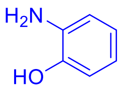
2a
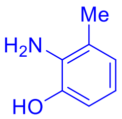
2b
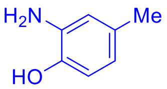
2c
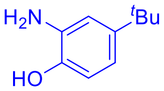
2d
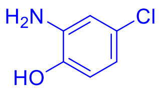
2e
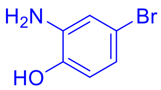
2f
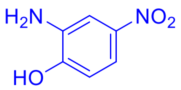
2g
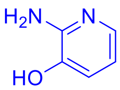
2h
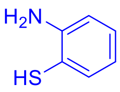
2i
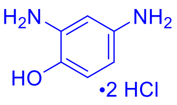
2j
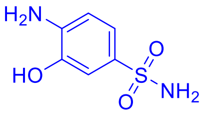
2k
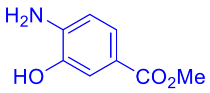
2l

2. ^{1}H NMR and ^{13}C NMR spectra of the products 3aa-3ar and 3ba-3ia.

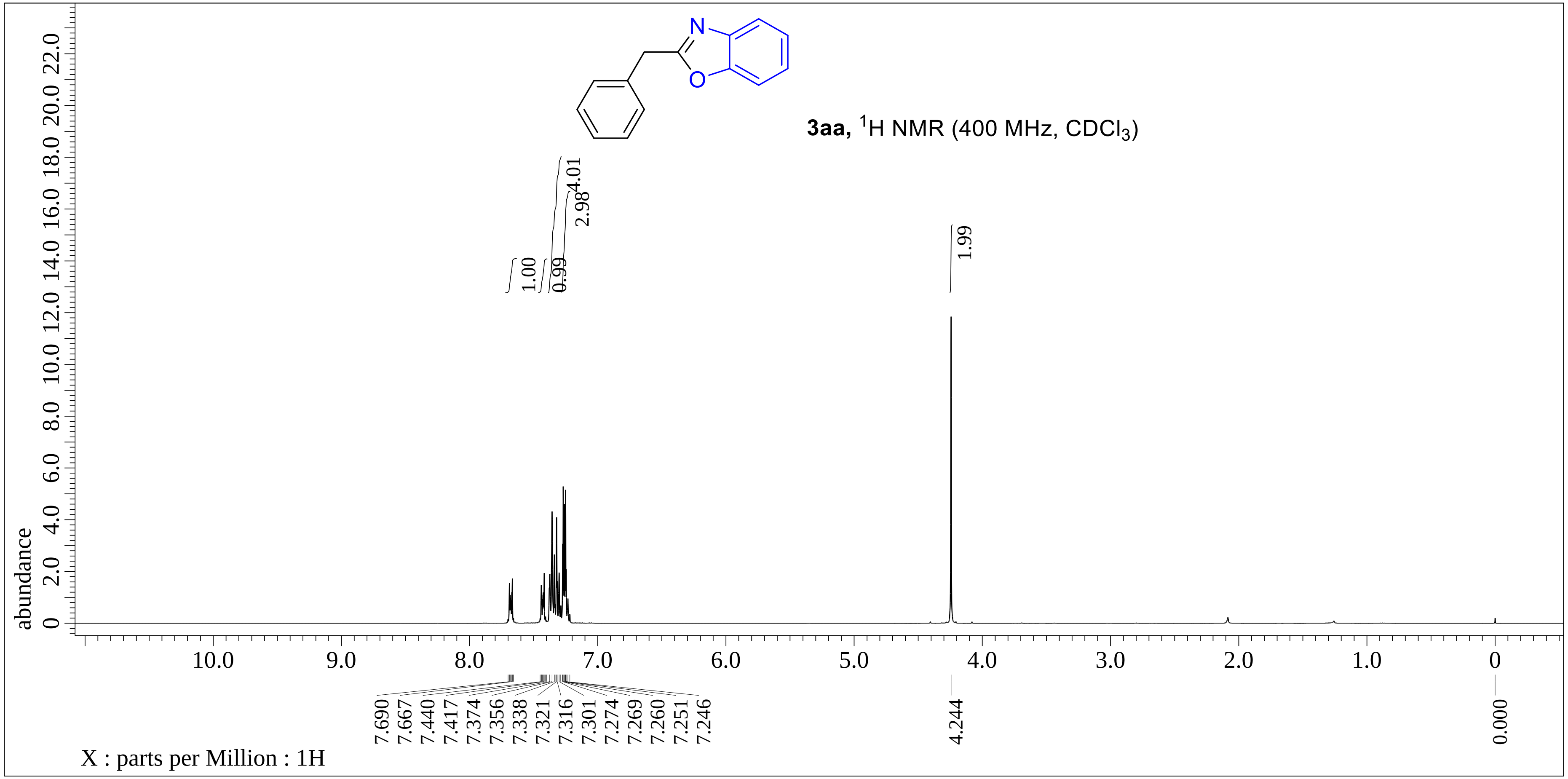

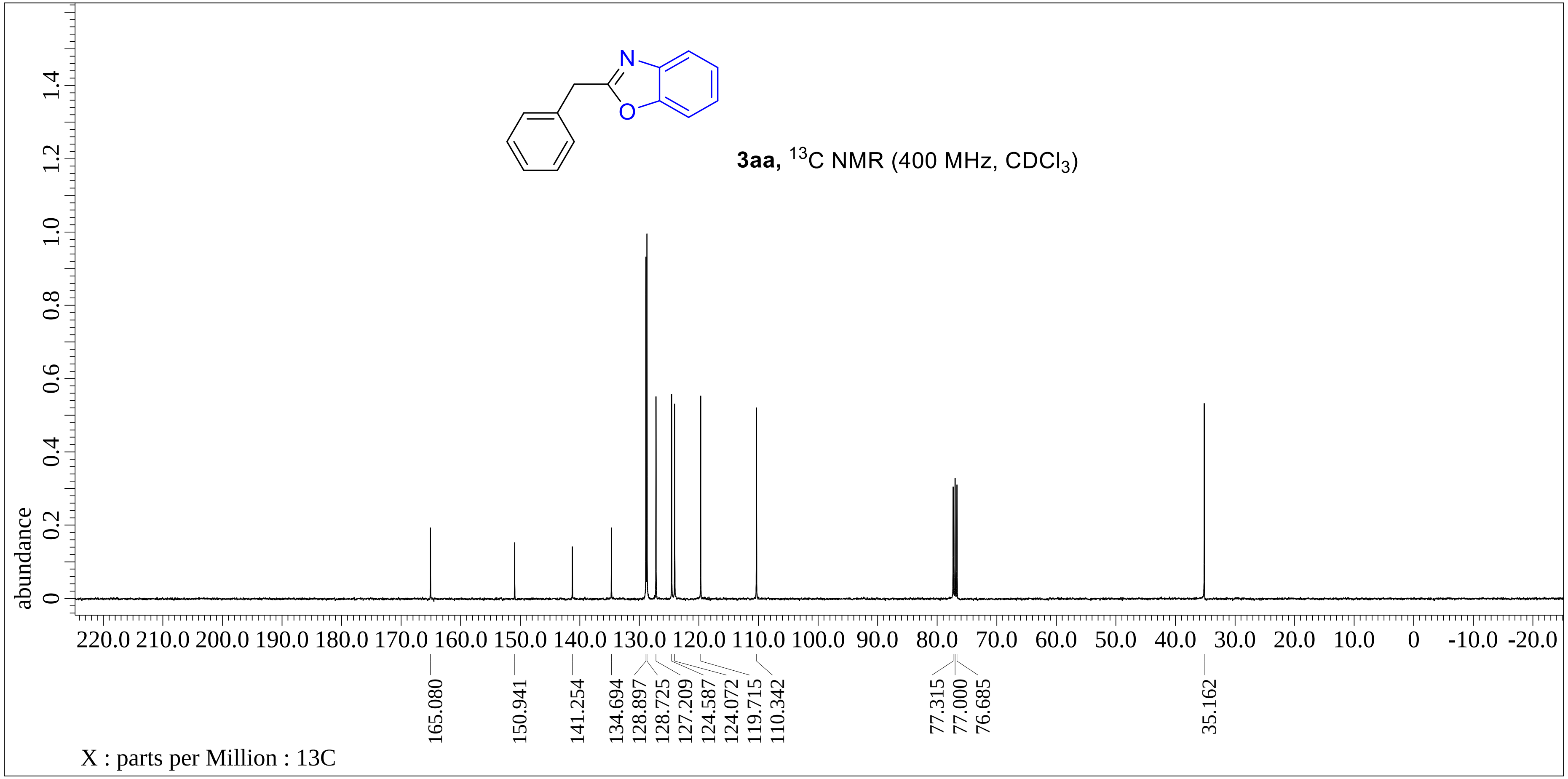
N
O
3aa, 13C NMR (400 MHz, CDCl3)
abundance
0
0.2
0.4
0.6
0.8
1.0
1.2
1.4
220.0 210.0 200.0 190.0 180.0 170.0 160.0 150.0 140.0 130.0 120.0 110.0 100.0 90.0 80.0 70.0 60.0 50.0 40.0 30.0 20.0 10.0 0 -10.0 -20.0
165.080
150.941
141.254
134.694
128.897
128.725
127.209
124.587
124.072
119.715
110.342
77.315
77.000
76.685
35.162
X : parts per Million : 13C

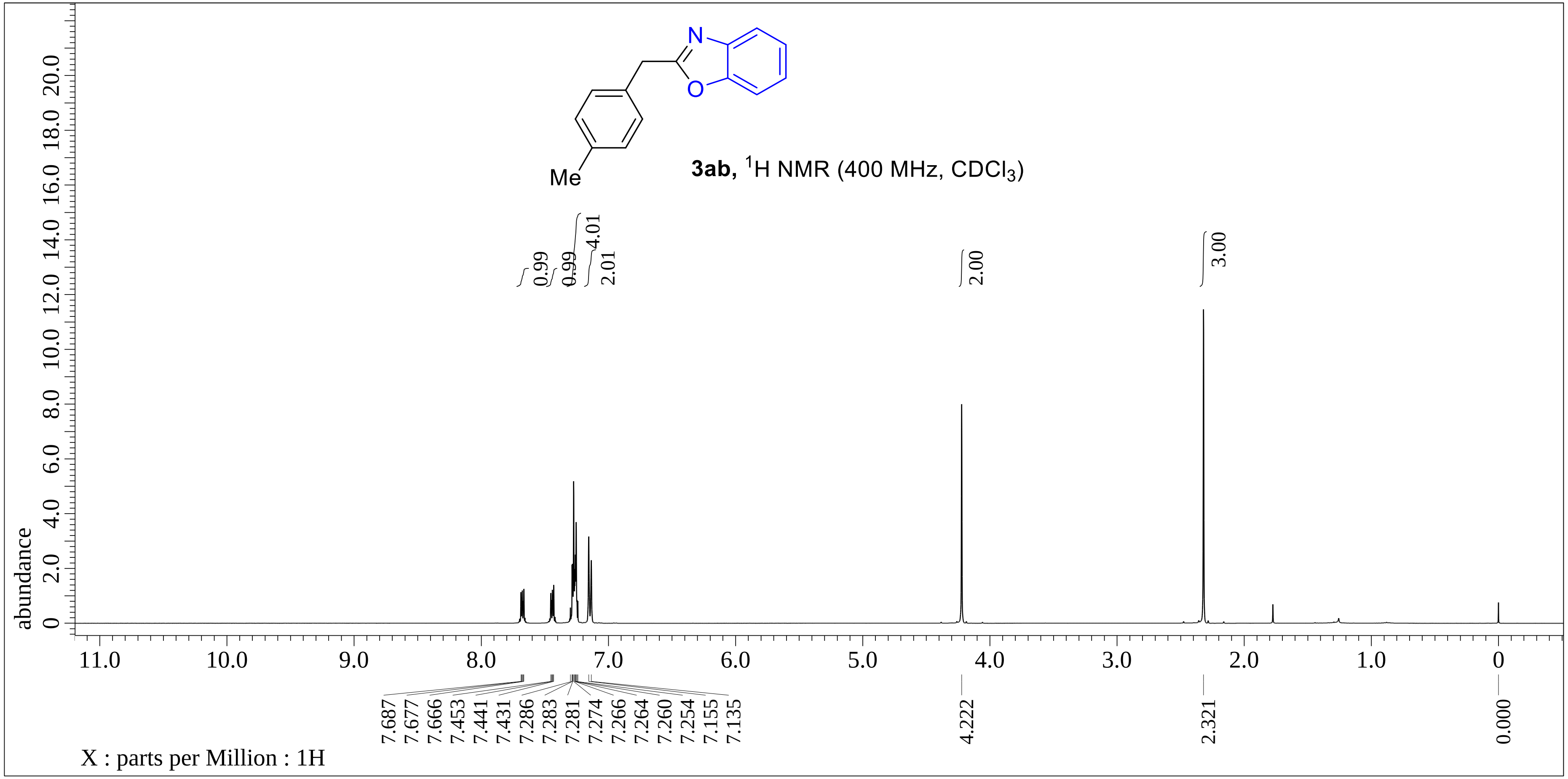

3ab, ^{1}H NMR (400 MHz, $CDCl_3$)
Me
N
O
0.99
0.99
4.01
2.01
2.00
3.00
7.687
7.677
7.666
7.453
7.441
7.431
7.286
7.283
7.281
7.274
7.266
7.264
7.260
7.254
7.155
7.135
4.222
2.321
0.000
abundance
X : parts per Million : 1H

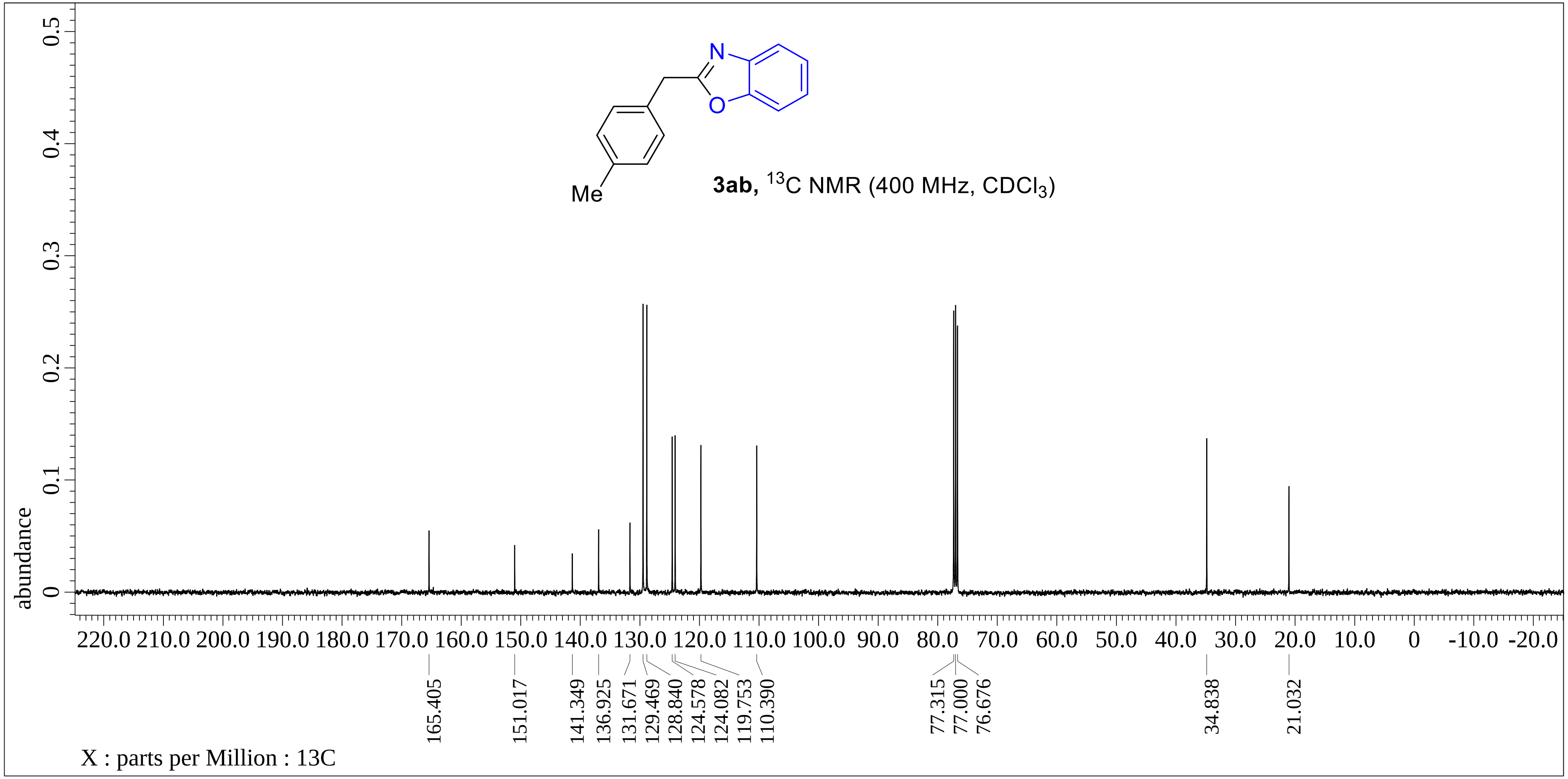

N
O
Me
3ab, ^{13}C NMR (400 MHz, $CDCl_3$)
abundance
0.5
0.4
0.3
0.2
0.1
0
220.0 210.0 200.0 190.0 180.0 170.0 160.0 150.0 140.0 130.0 120.0 110.0 100.0 90.0 80.0 70.0 60.0 50.0 40.0 30.0 20.0 10.0 0 -10.0 -20.0
165.405
151.017
141.349
136.925
131.671
129.469
128.840
124.578
124.082
119.753
110.390
77.315
77.000
76.676
34.838
21.032
X : parts per Million : 13C

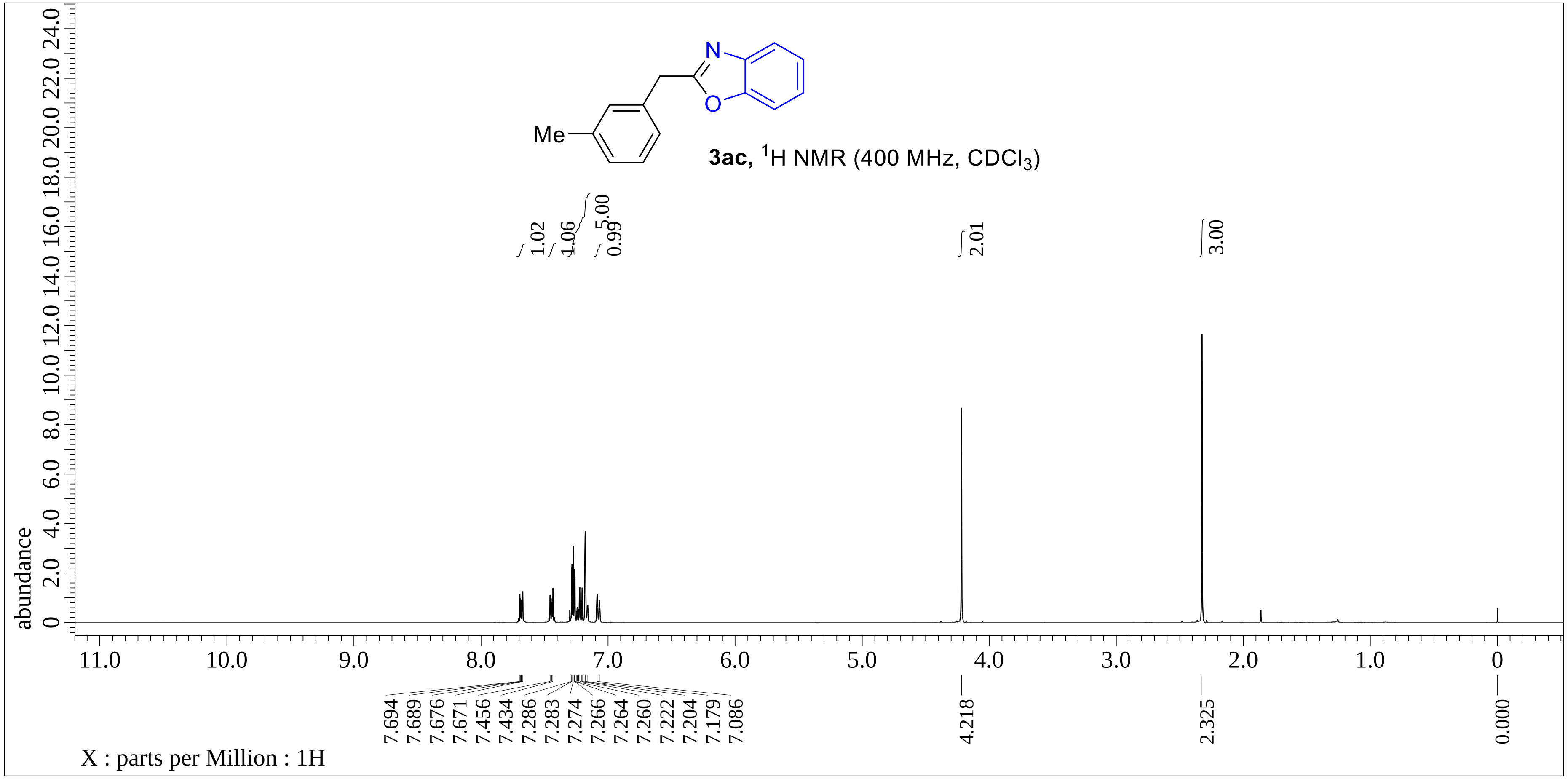

Me
N
O
3ac, ^{1}H NMR (400 MHz, $CDCl_3$)
1.02
1.06
5.00
0.99
2.01
3.00
abundance
0
2.0
4.0
6.0
8.0
10.0
12.0
14.0
16.0
18.0
20.0
22.0
24.0
11.0
10.0
9.0
8.0
7.0
6.0
5.0
4.0
3.0
2.0
1.0
0
7.694
7.689
7.676
7.671
7.456
7.434
7.286
7.283
7.274
7.266
7.264
7.260
7.222
7.204
7.179
7.086
4.218
2.325
0.000
X : parts per Million : 1H

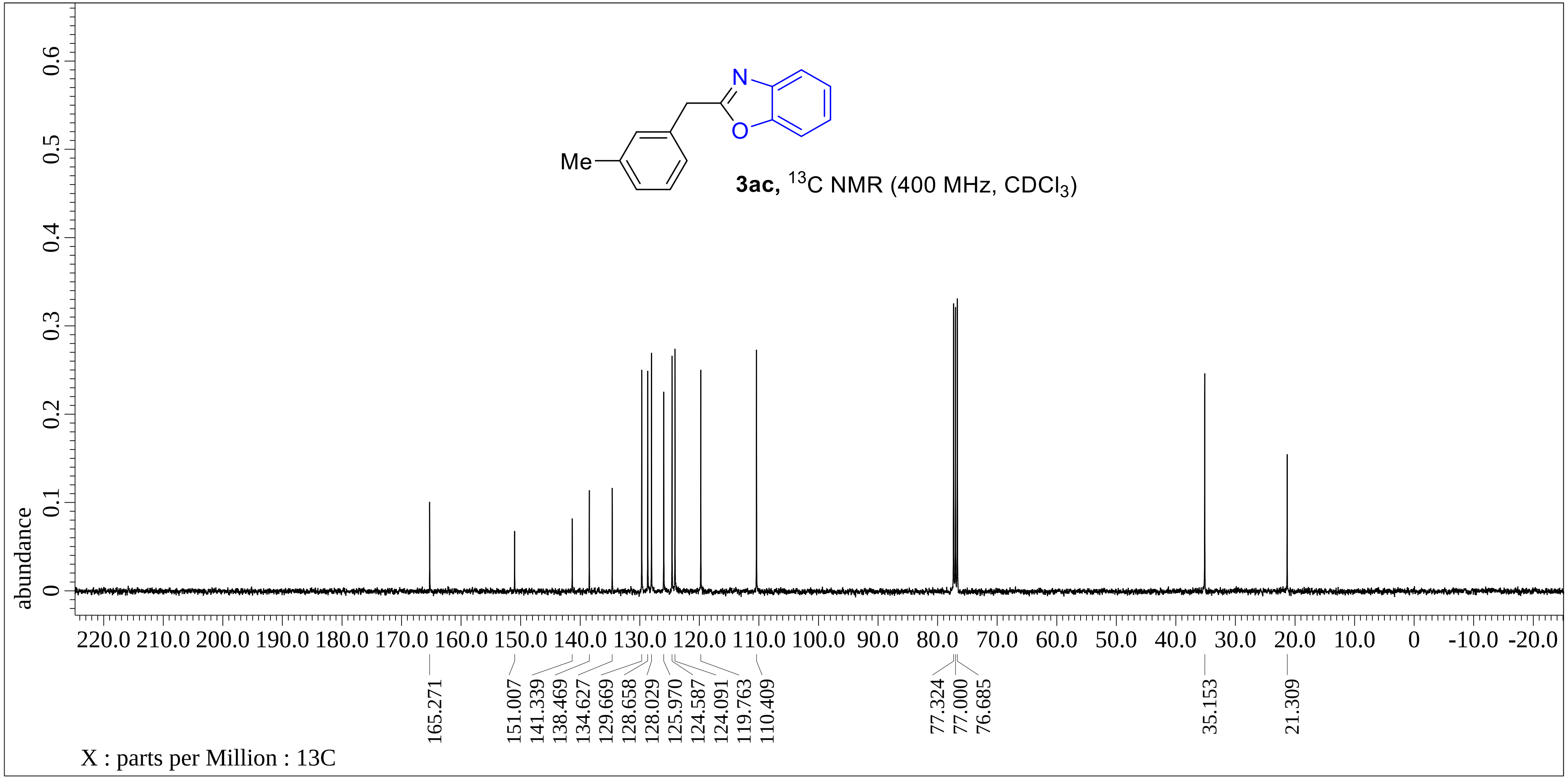

N
O
Me
3ac, ^{13}C NMR (400 MHz, $CDCl_3$)
abundance
0
0.1
0.2
0.3
0.4
0.5
0.6
220.0 210.0 200.0 190.0 180.0 170.0 160.0 150.0 140.0 130.0 120.0 110.0 100.0 90.0 80.0 70.0 60.0 50.0 40.0 30.0 20.0 10.0 0 -10.0 -20.0
165.271
151.007
141.339
138.469
134.627
129.669
128.658
128.029
125.970
124.587
124.091
119.763
110.409
77.324
77.000
76.685
35.153
21.309
X : parts per Million : 13C

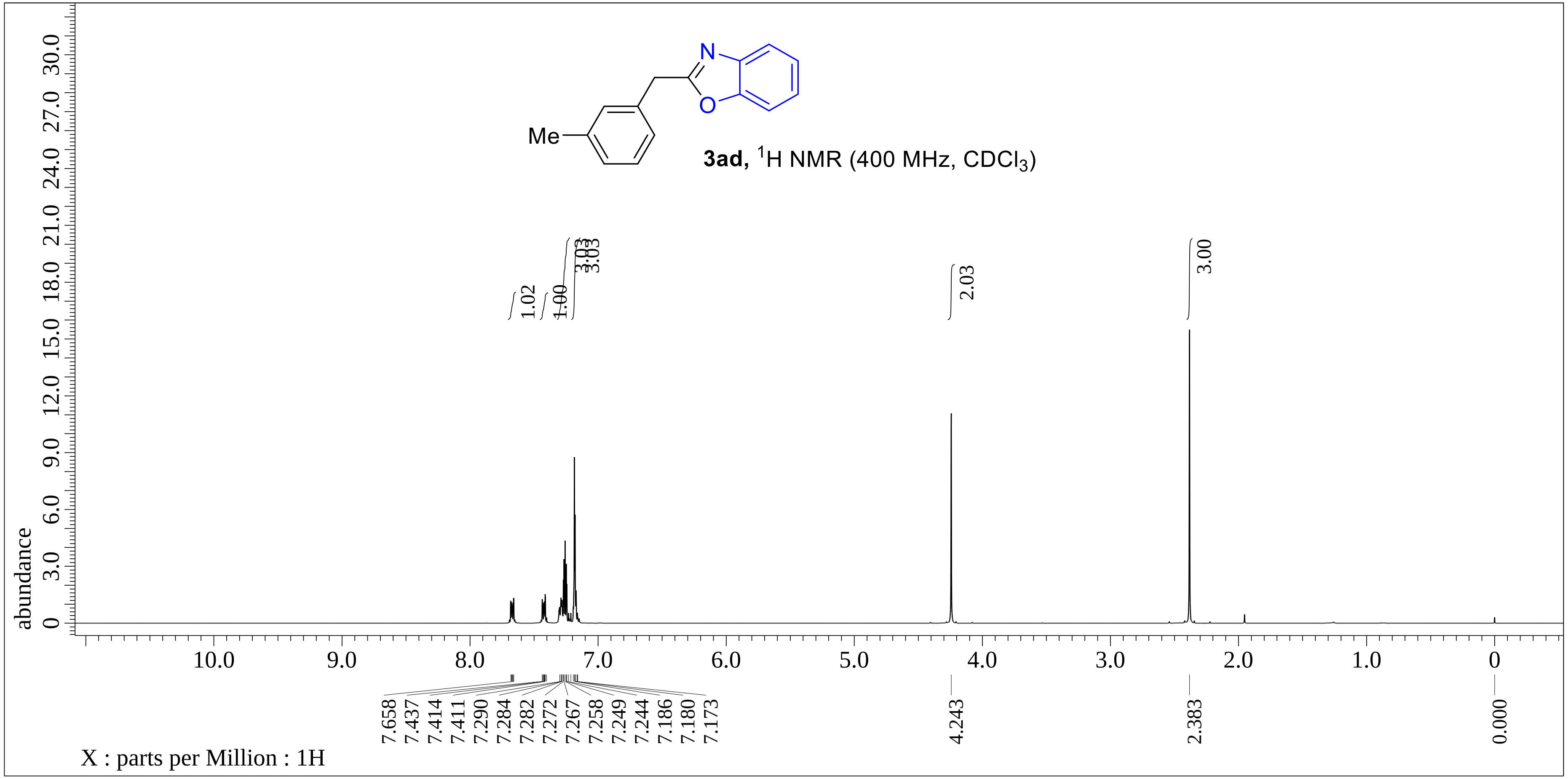

Me
N
O
3ad, ^{1}H NMR (400 MHz, $CDCl_3$)
abundance
X : parts per Million : 1H

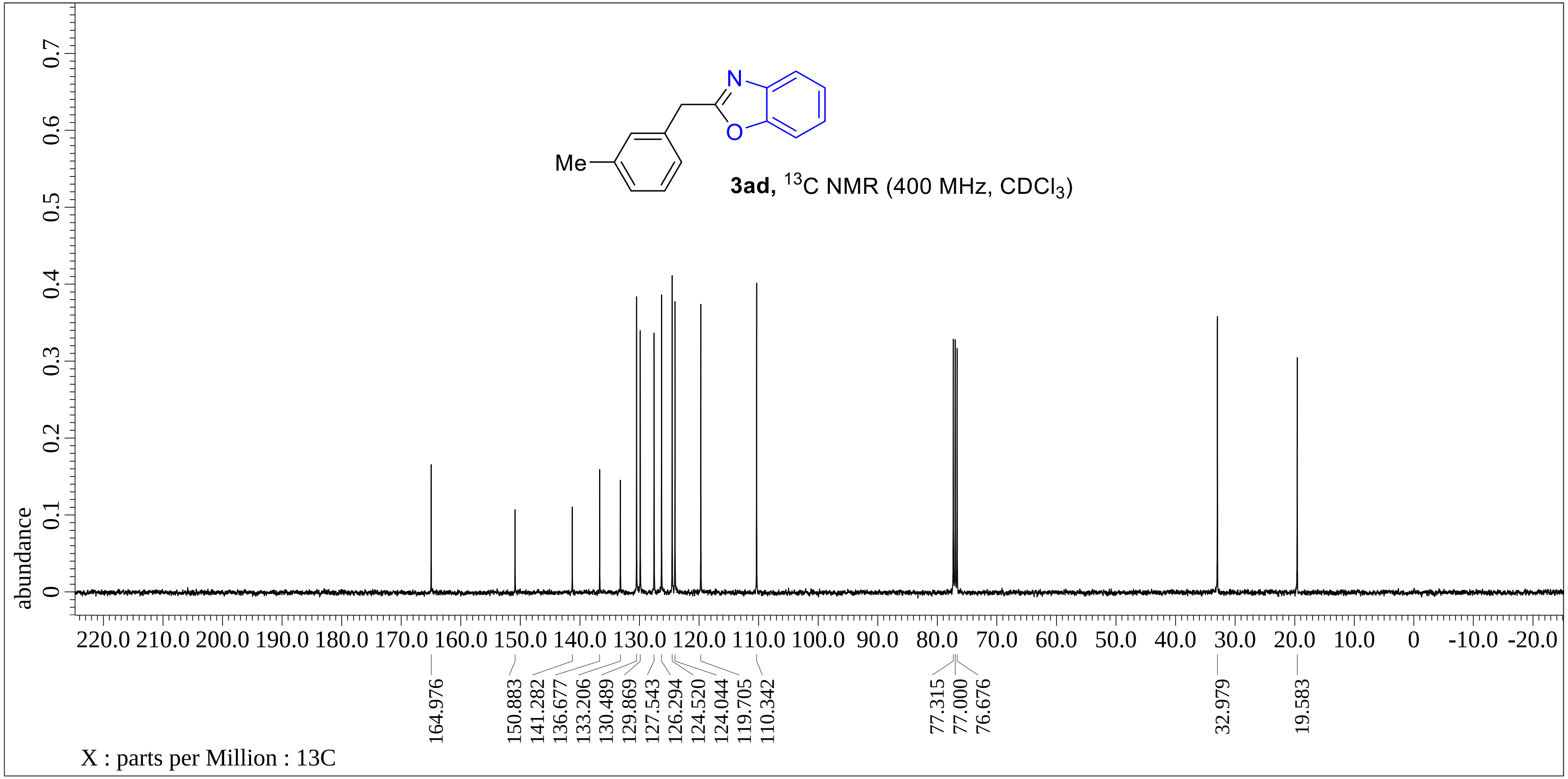
Me
N
O
3ad, 13C NMR (400 MHz, CDCl3)
abundance
0 0.1 0.2 0.3 0.4 0.5 0.6 0.7
220.0 210.0 200.0 190.0 180.0 170.0 160.0 150.0 140.0 130.0 120.0 110.0 100.0 90.0 80.0 70.0 60.0 50.0 40.0 30.0 20.0 10.0 0 -10.0 -20.0
164.976
150.883
141.282
136.677
133.206
130.489
129.869
127.543
126.294
124.520
124.044
119.705
110.342
77.315
77.000
76.676
32.979
19.583
X : parts per Million : 13C

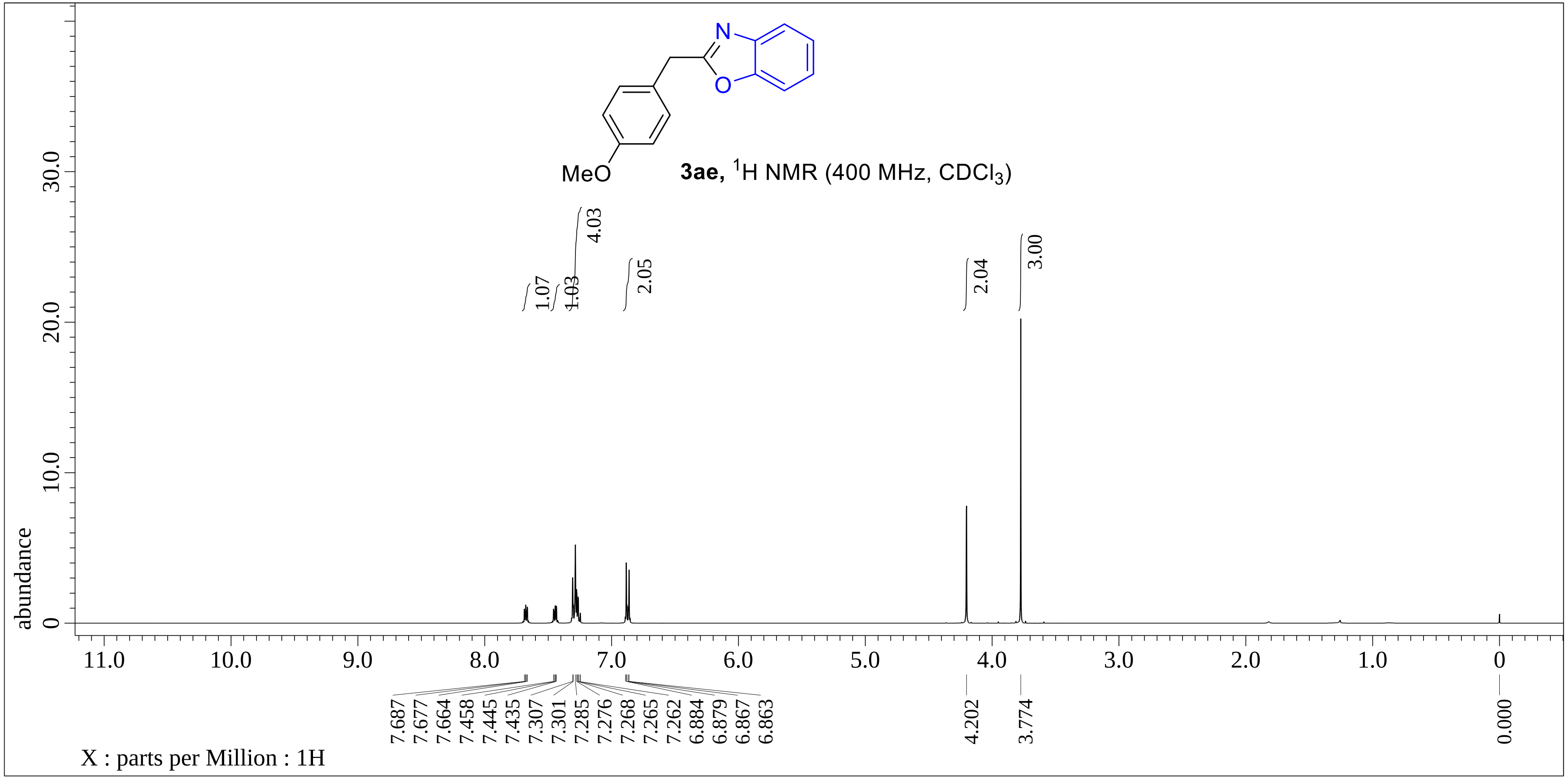
N
O
MeO
3ae, 1H NMR (400 MHz, CDCl3)
1.07
1.03
4.03
2.05
2.04
3.00
abundance
0
10.0
20.0
30.0
11.0
10.0
9.0
8.0
7.0
6.0
5.0
4.0
3.0
2.0
1.0
0
7.687
7.677
7.664
7.458
7.445
7.435
7.307
7.301
7.285
7.276
7.268
7.265
7.262
6.884
6.879
6.867
6.863
4.202
3.774
0.000
X : parts per Million : 1H

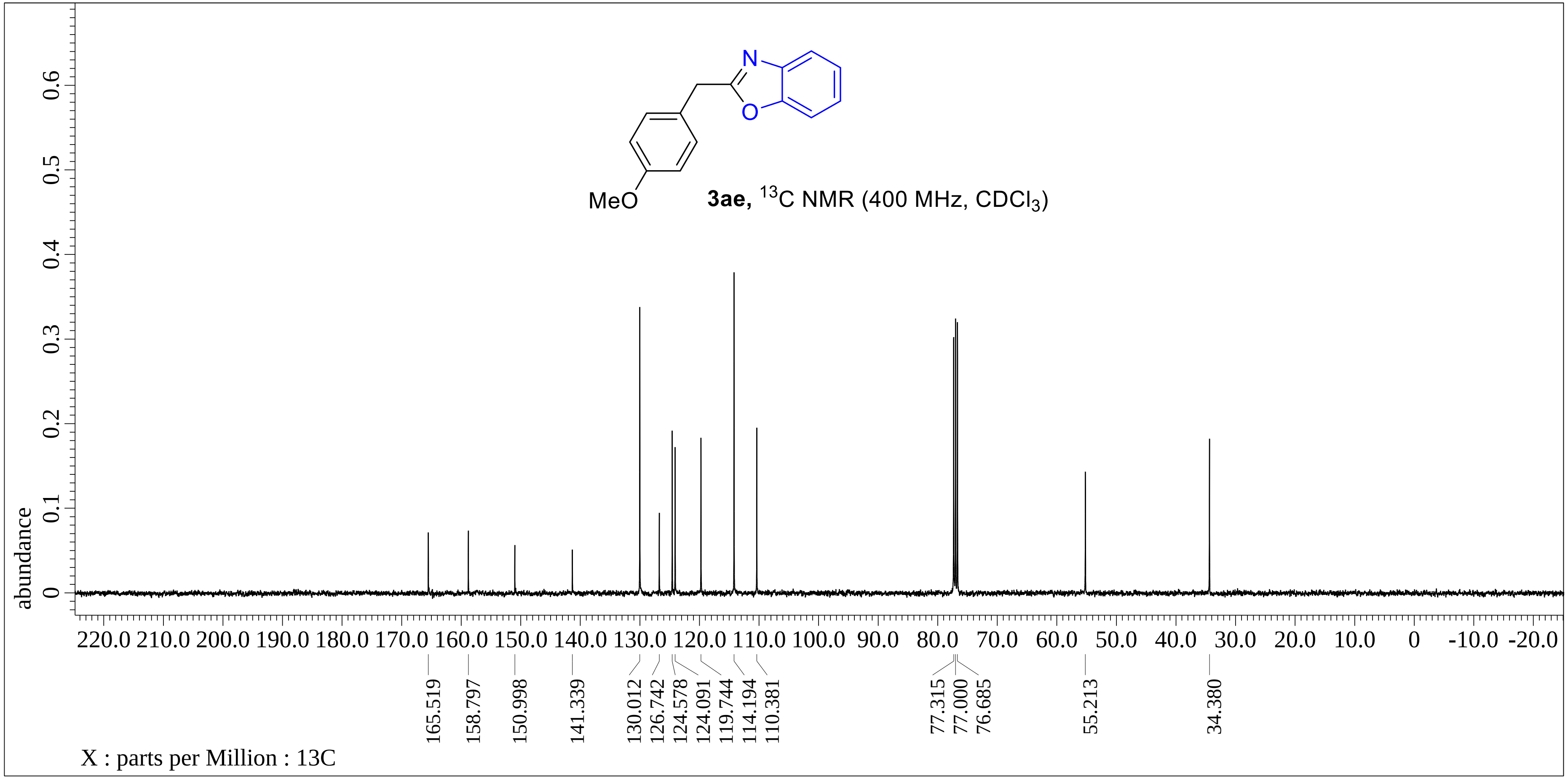

N
O
MeO
3ae, ^{13}C NMR (400 MHz, $CDCl_3$)
abundance
0 0.1 0.2 0.3 0.4 0.5 0.6
220.0 210.0 200.0 190.0 180.0 170.0 160.0 150.0 140.0 130.0 120.0 110.0 100.0 90.0 80.0 70.0 60.0 50.0 40.0 30.0 20.0 10.0 0 -10.0 -20.0
165.519
158.797
150.998
141.339
130.012
126.742
124.578
124.091
119.744
114.194
110.381
77.315
77.000
76.685
55.213
34.380
X : parts per Million : 13C

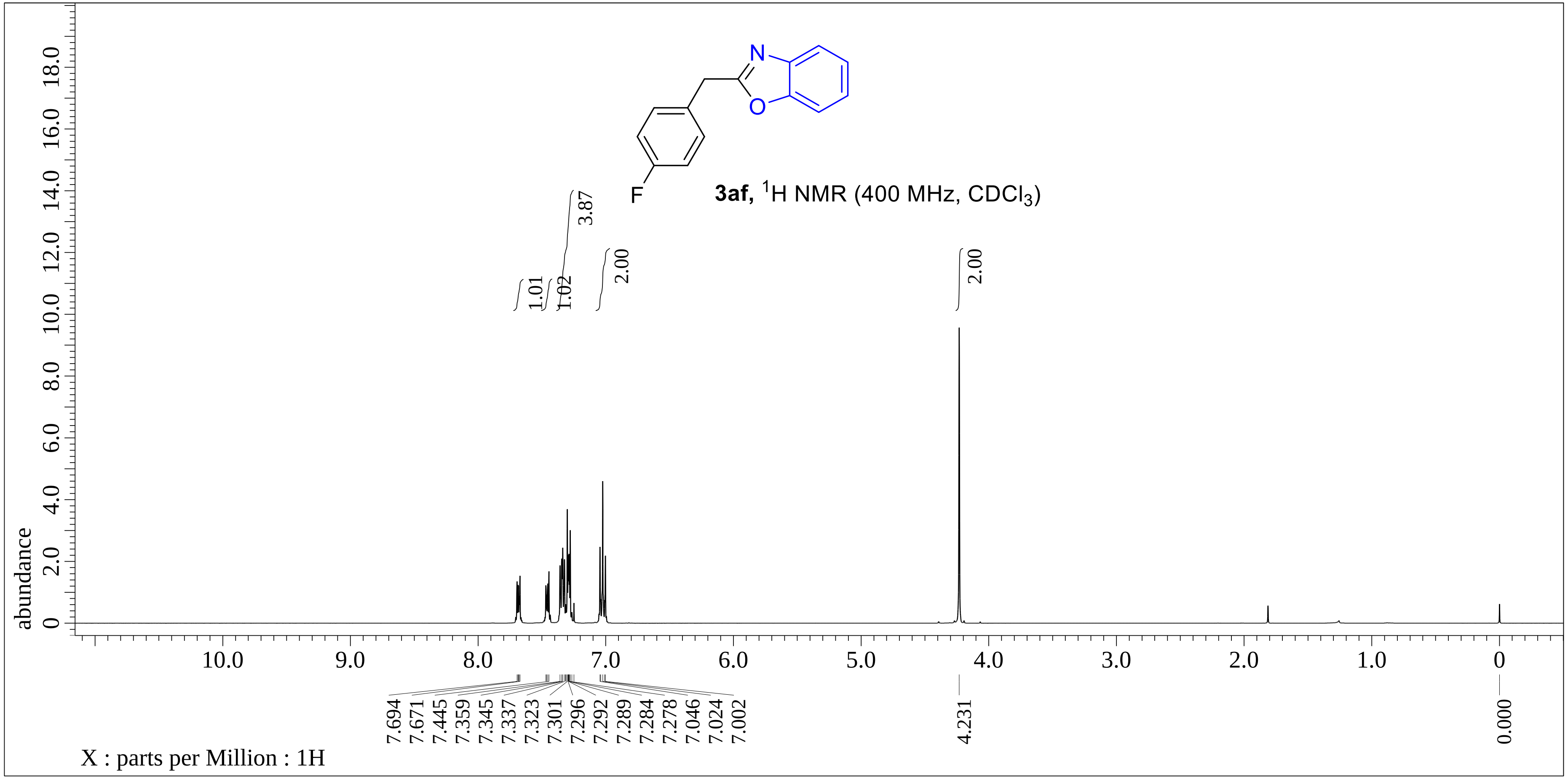

N
O
F
3af, 1H NMR (400 MHz, CDCl3)
abundance
X : parts per Million : 1H
1.01
1.02
3.87
2.00
2.00
7.694
7.671
7.445
7.359
7.345
7.337
7.323
7.301
7.296
7.292
7.289
7.284
7.278
7.046
7.024
7.002
4.231
0.000

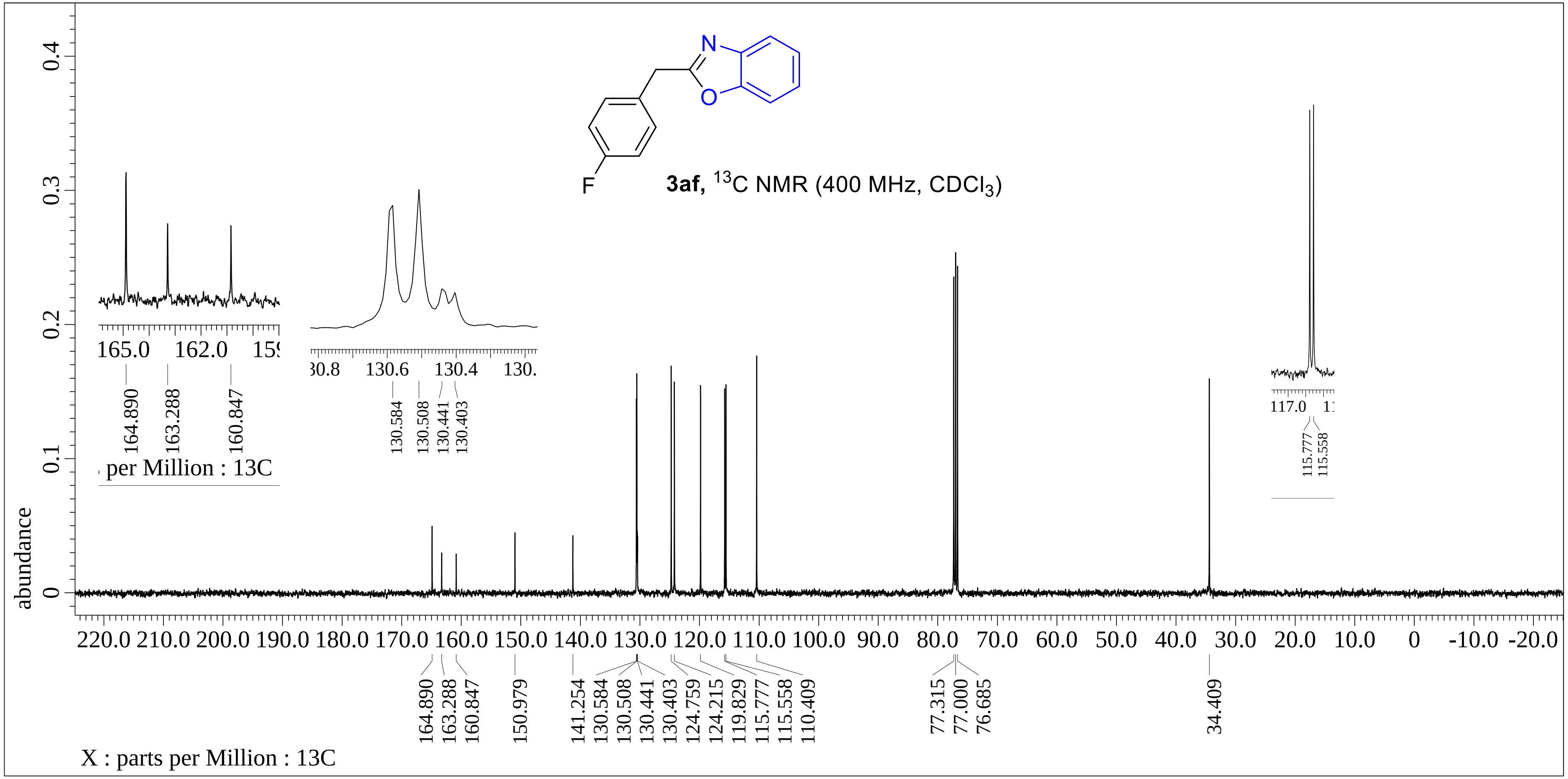
3af, ^{13}C NMR (400 MHz, CDCl$_3$)
N
O
F
abundance
0.4
0.3
0.2
0.1
0
164.890
163.288
160.847
150.979
141.254
130.584
130.508
130.441
130.403
124.759
124.215
119.829
115.777
115.558
110.409
77.315
77.000
76.685
34.409
X : parts per Million : 13C

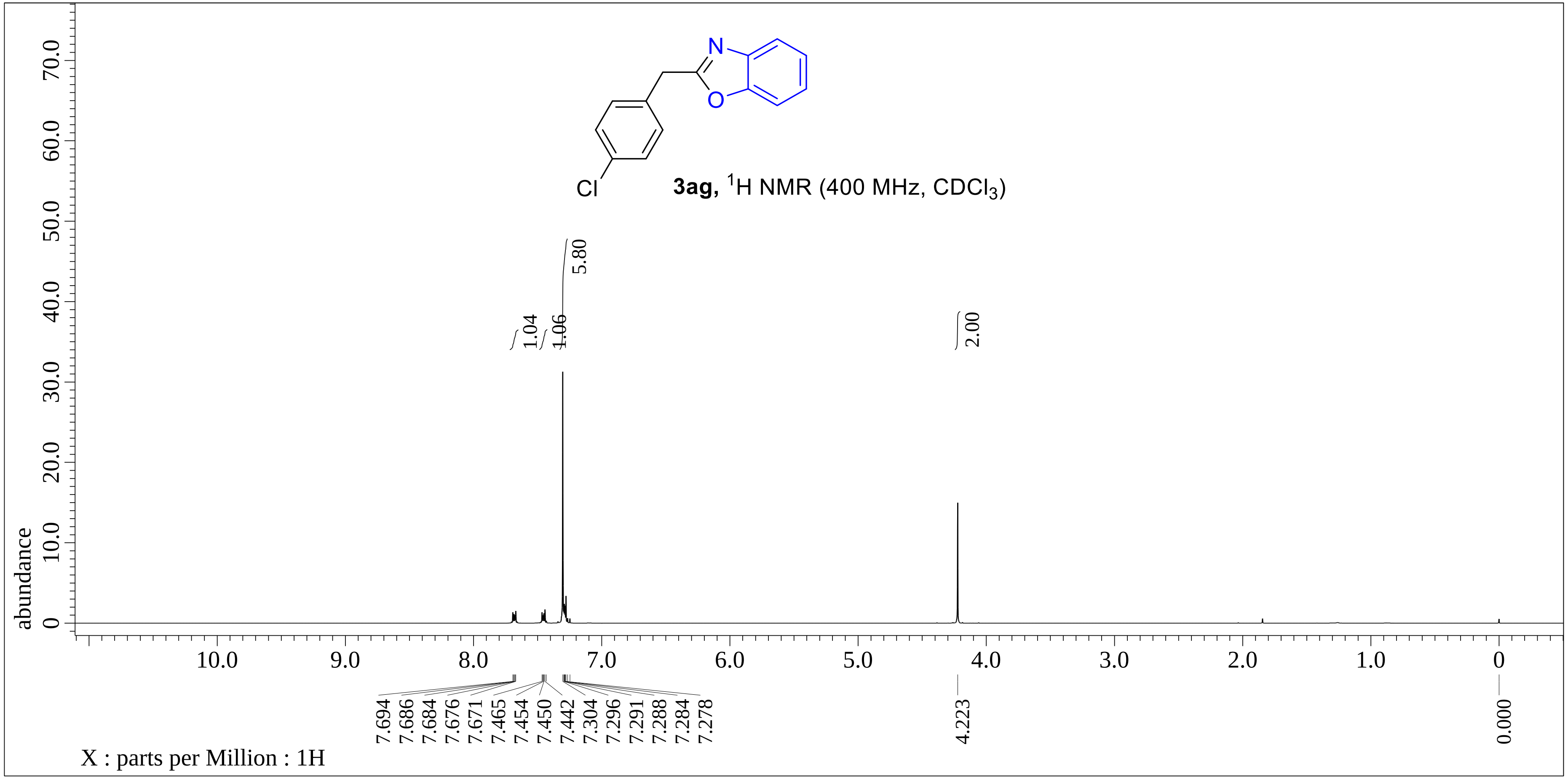

N
O
Cl
3ag, ¹H NMR (400 MHz, CDCl₃)
5.80
1.04
1.06
2.00
abundance
X : parts per Million : 1H
7.694
7.686
7.684
7.676
7.671
7.465
7.454
7.450
7.442
7.304
7.296
7.291
7.288
7.284
7.278
4.223
0.000

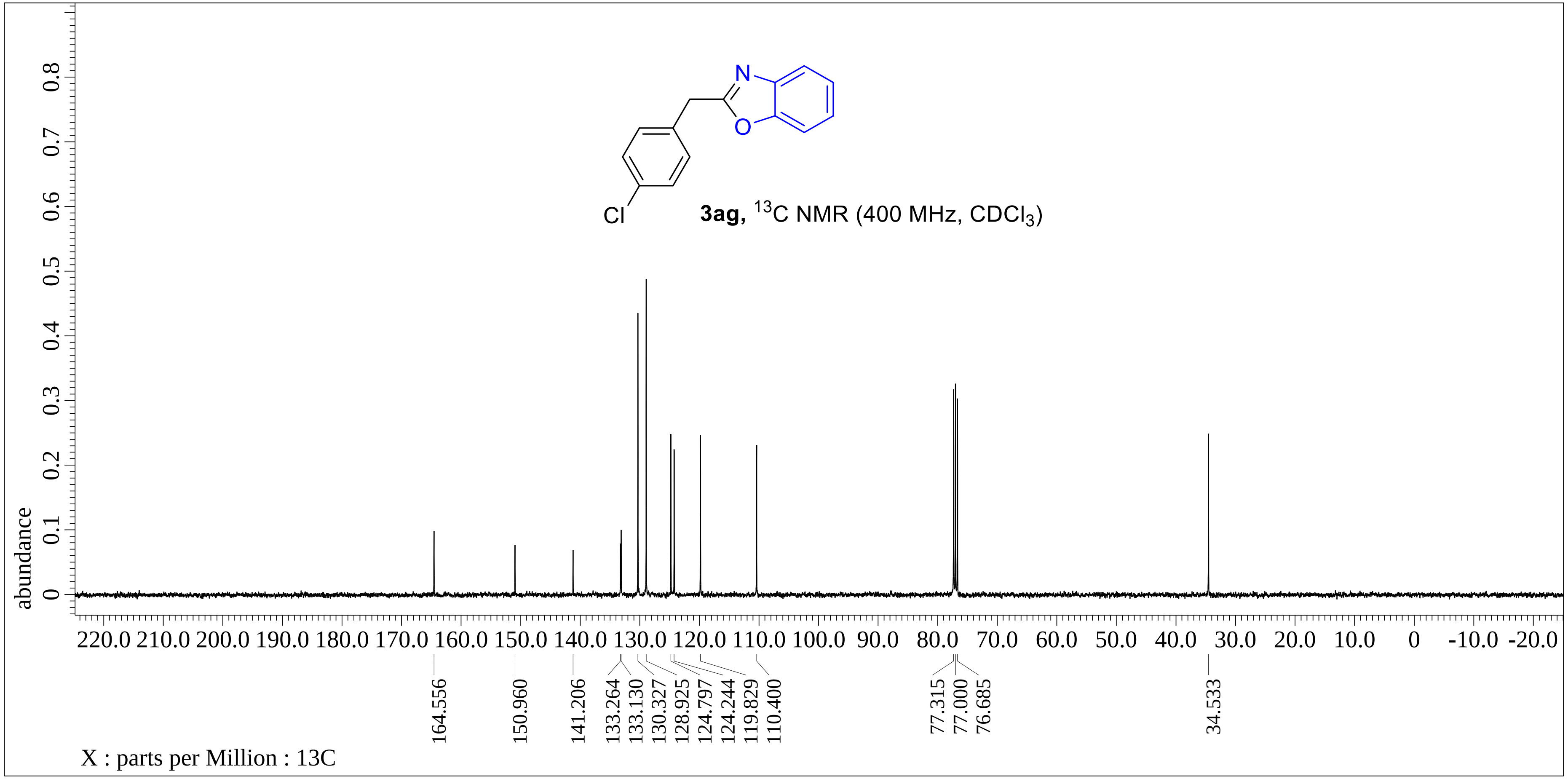

3ag, ^{13}C NMR (400 MHz, $CDCl_3$)
N
O
Cl
abundance
0 0.1 0.2 0.3 0.4 0.5 0.6 0.7 0.8
220.0 210.0 200.0 190.0 180.0 170.0 160.0 150.0 140.0 130.0 120.0 110.0 100.0 90.0 80.0 70.0 60.0 50.0 40.0 30.0 20.0 10.0 0 -10.0 -20.0
164.556
150.960
141.206
133.264
133.130
130.327
128.925
124.797
124.244
119.829
110.400
77.315
77.000
76.685
34.533
X : parts per Million : 13C

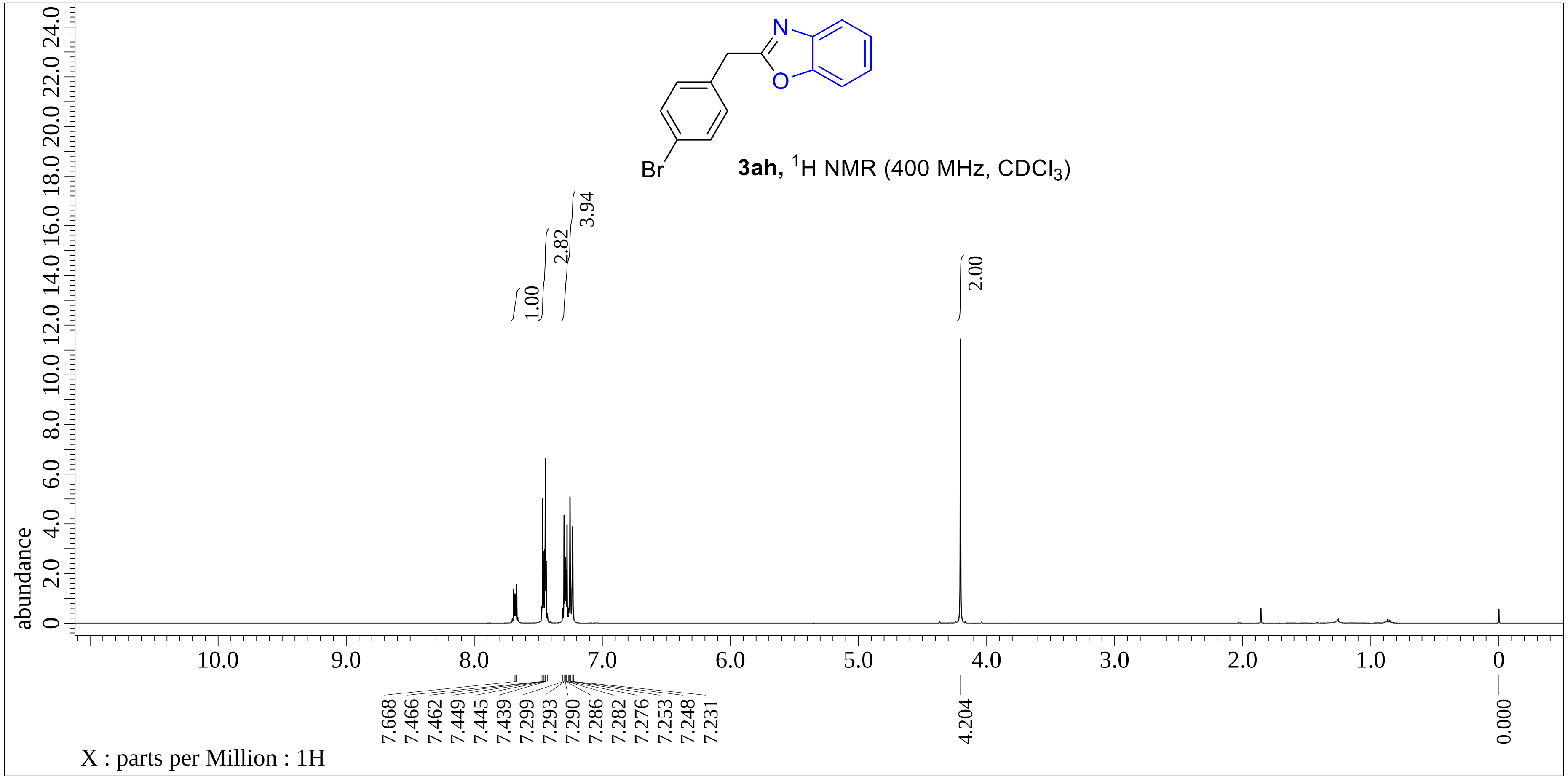

N
O
Br
3ah, ¹H NMR (400 MHz, CDCl₃)
1.00
2.82
3.94
2.00
abundance
0
2.0
4.0
6.0
8.0
10.0
12.0
14.0
16.0
18.0
20.0
22.0
24.0
10.0
9.0
8.0
7.0
6.0
5.0
4.0
3.0
2.0
1.0
0
7.668
7.466
7.462
7.449
7.445
7.439
7.299
7.293
7.290
7.286
7.282
7.276
7.253
7.248
7.231
4.204
0.000
X : parts per Million : 1H

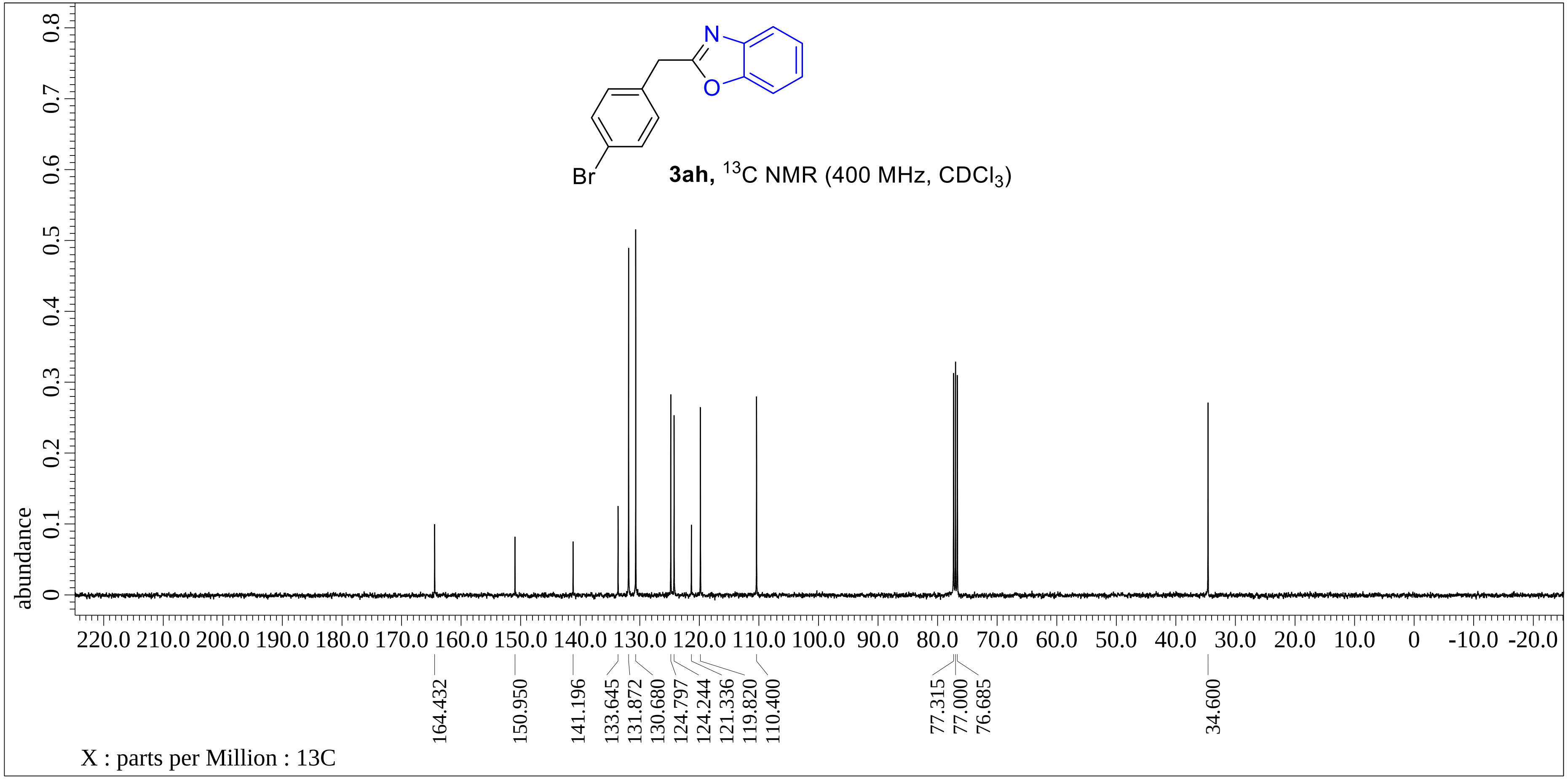

N
O
Br
3ah, ^{13}C NMR (400 MHz, $CDCl_3$)
abundance
0
0.1
0.2
0.3
0.4
0.5
0.6
0.7
0.8
220.0 210.0 200.0 190.0 180.0 170.0 160.0 150.0 140.0 130.0 120.0 110.0 100.0 90.0 80.0 70.0 60.0 50.0 40.0 30.0 20.0 10.0 0 -10.0 -20.0
164.432
150.950
141.196
133.645
131.872
130.680
124.797
124.244
121.336
119.820
110.400
77.315
77.000
76.685
34.600
X : parts per Million : 13C

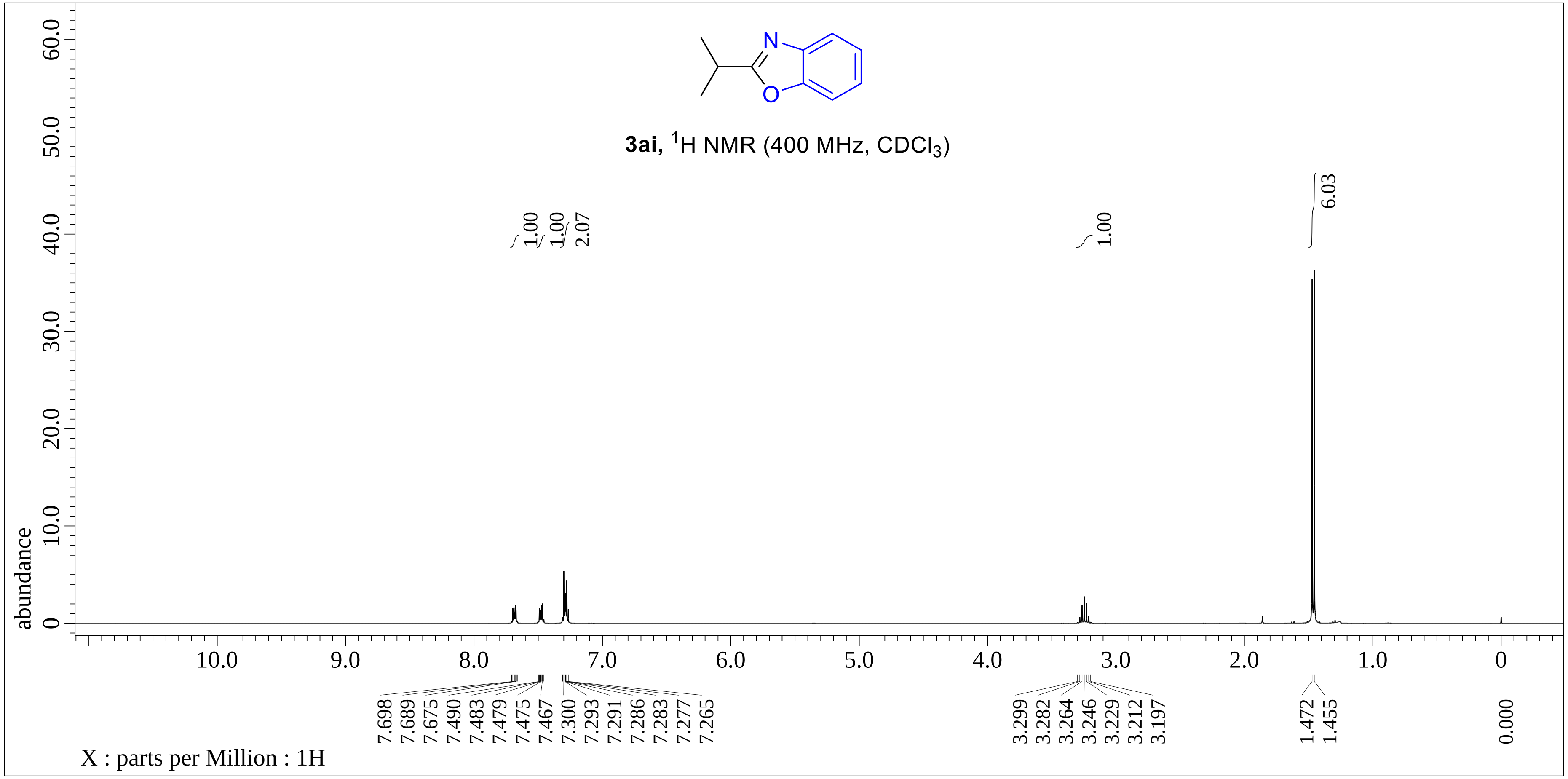
N
O
3ai, ^{1}H NMR (400 MHz, $CDCl_3$)
abundance
60.0
50.0
40.0
30.0
20.0
10.0
0
1.00
1.00
2.07
1.00
6.03
10.0
9.0
8.0
7.0
6.0
5.0
4.0
3.0
2.0
1.0
0
7.698
7.689
7.675
7.490
7.483
7.479
7.475
7.467
7.300
7.293
7.291
7.286
7.283
7.277
7.265
3.299
3.282
3.264
3.246
3.229
3.212
3.197
1.472
1.455
0.000
X : parts per Million : 1H

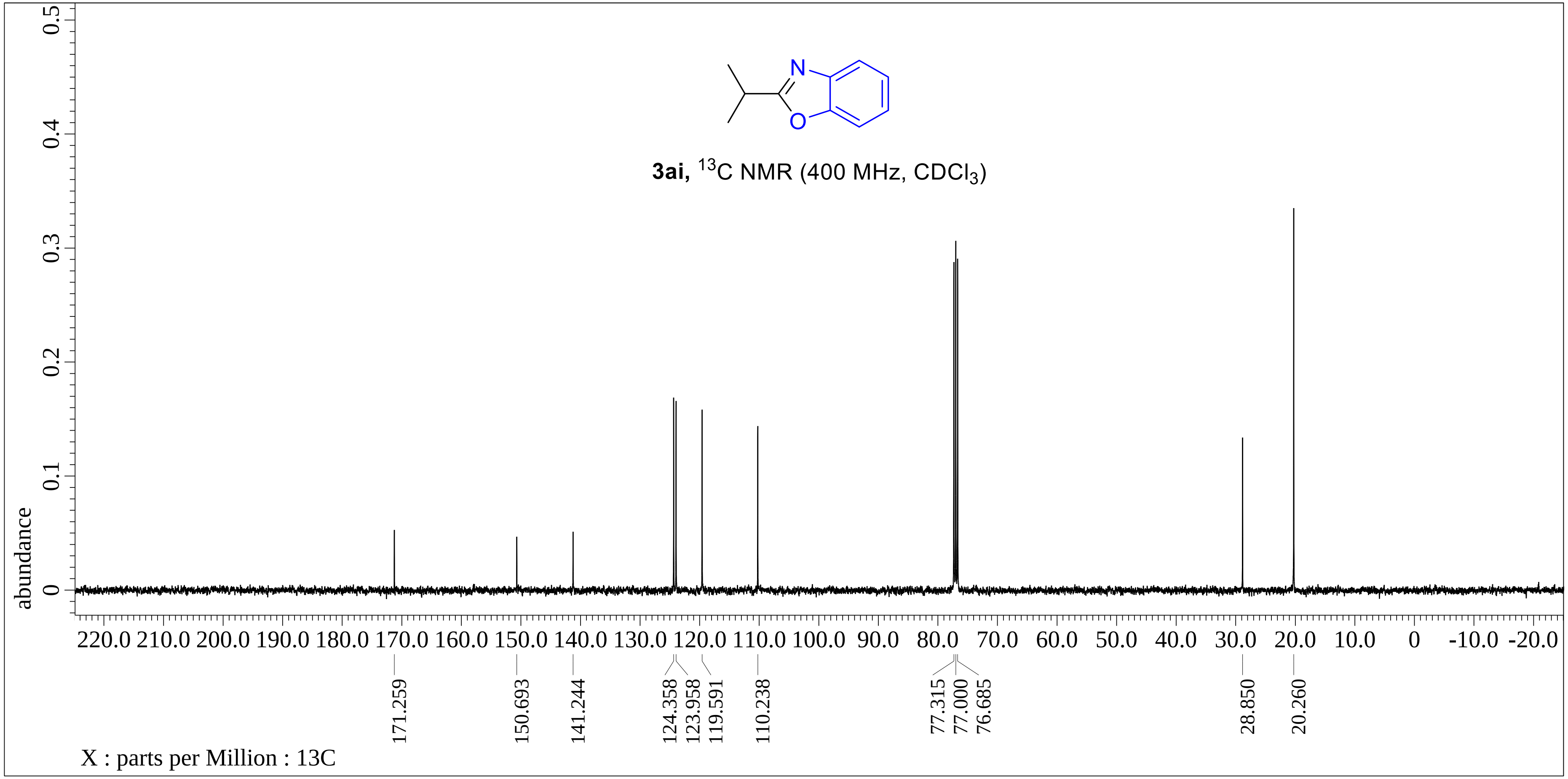
N
O
3ai, 13C NMR (400 MHz, CDCl3)
abundance
0.5
0.4
0.3
0.2
0.1
0
220.0 210.0 200.0 190.0 180.0 170.0 160.0 150.0 140.0 130.0 120.0 110.0 100.0 90.0 80.0 70.0 60.0 50.0 40.0 30.0 20.0 10.0 0 -10.0 -20.0
171.259
150.693
141.244
124.358
123.958
119.591
110.238
77.315
77.000
76.685
28.850
20.260
X : parts per Million : 13C

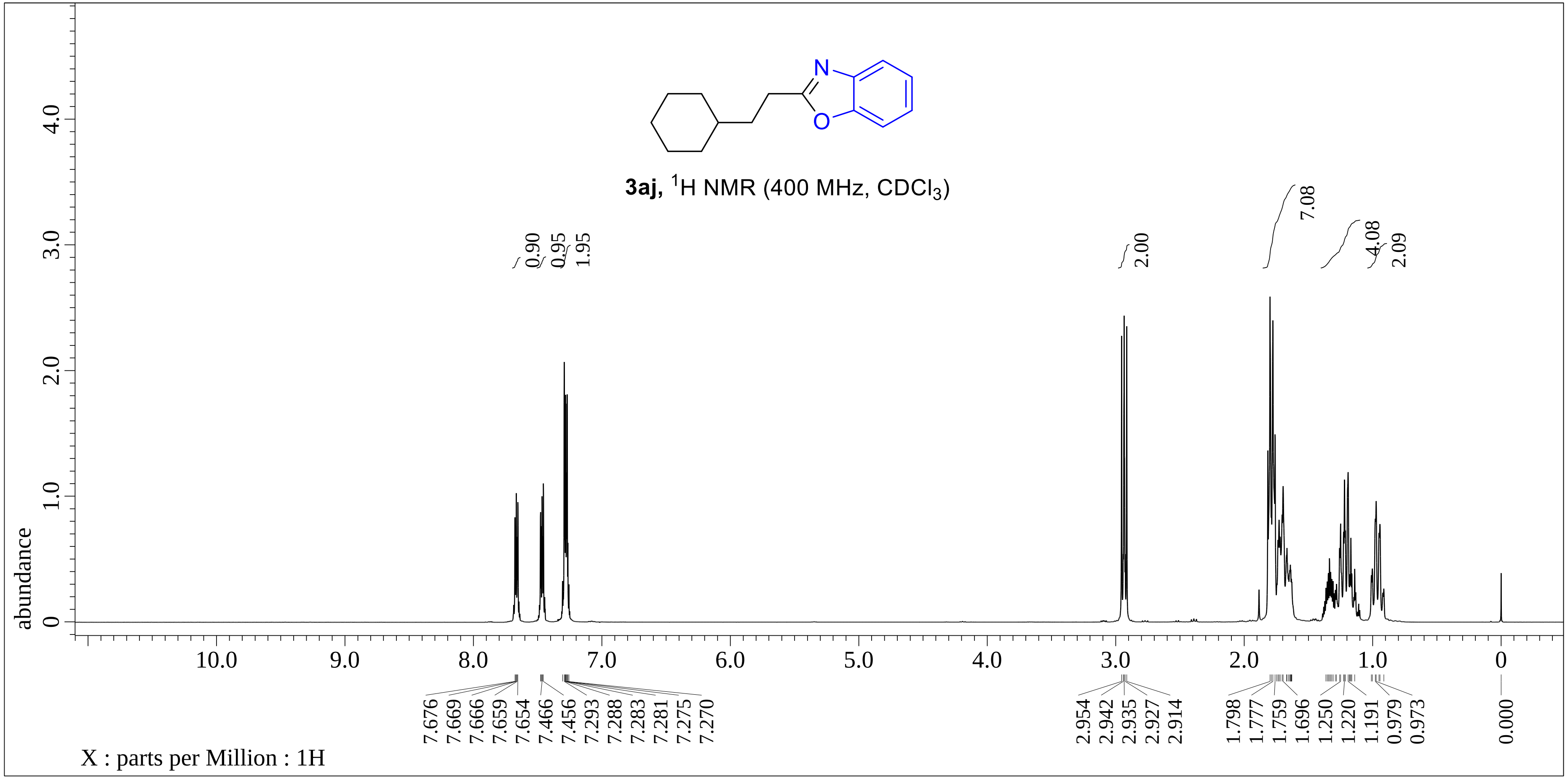
3aj, 1H NMR (400 MHz, CDCl3)
abundance
X : parts per Million : 1H
0.90
0.95
1.95
2.00
7.08
4.08
2.09
7.676
7.669
7.666
7.659
7.654
7.466
7.456
7.293
7.288
7.283
7.281
7.275
7.270
2.954
2.942
2.935
2.927
2.914
1.798
1.777
1.759
1.696
1.250
1.220
1.191
0.979
0.973
0.000

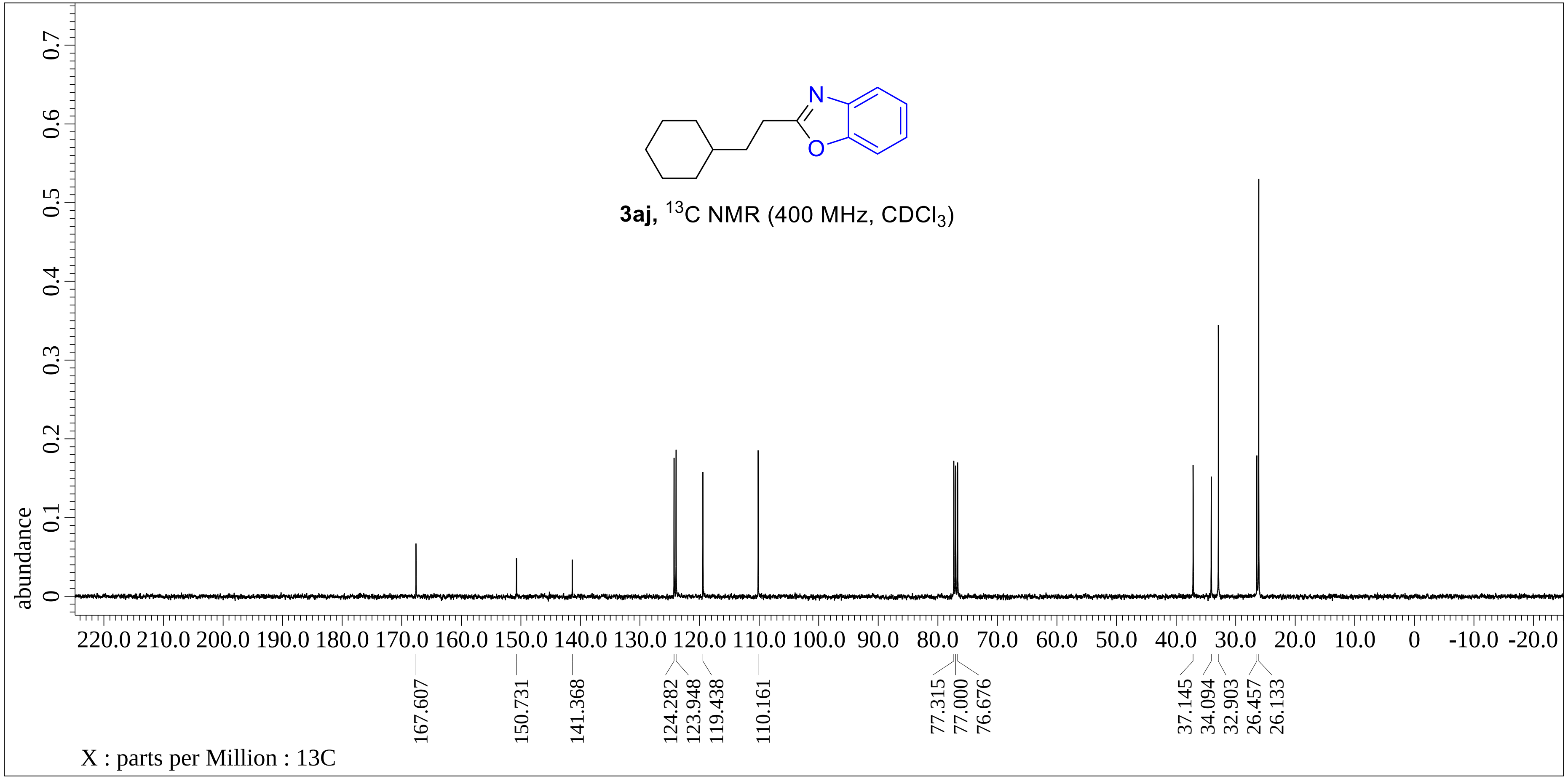
3aj, 13C NMR (400 MHz, CDCl3)
N
O
abundance
0
0.1
0.2
0.3
0.4
0.5
0.6
0.7
220.0 210.0 200.0 190.0 180.0 170.0 160.0 150.0 140.0 130.0 120.0 110.0 100.0 90.0 80.0 70.0 60.0 50.0 40.0 30.0 20.0 10.0 0 -10.0 -20.0
167.607
150.731
141.368
124.282
123.948
119.438
110.161
77.315
77.000
76.676
37.145
34.094
32.903
26.457
26.133
X : parts per Million : 13C

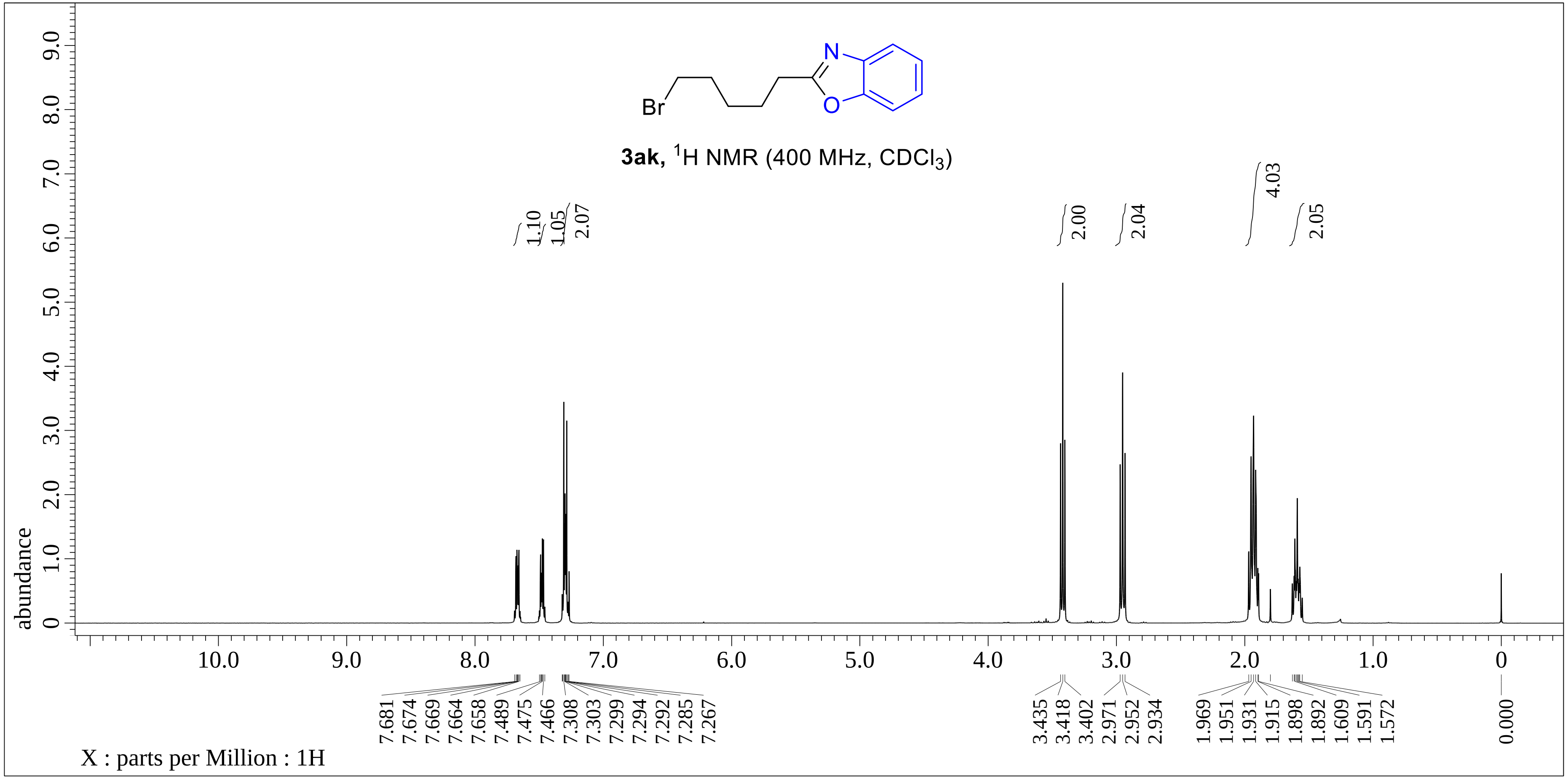
Br
N
O
3ak, ^{1}H NMR (400 MHz, $CDCl_3$)
1.10
1.05
2.07
2.00
2.04
4.03
2.05
abundance
X : parts per Million : 1H
7.681
7.674
7.669
7.664
7.658
7.489
7.475
7.466
7.308
7.303
7.299
7.294
7.292
7.285
7.267
3.435
3.418
3.402
2.971
2.952
2.934
1.969
1.951
1.931
1.915
1.898
1.892
1.609
1.591
1.572
0.000

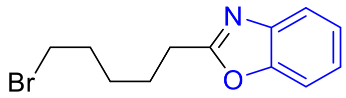

3ak, ^{13}C NMR (400 MHz, $CDCl_3$)

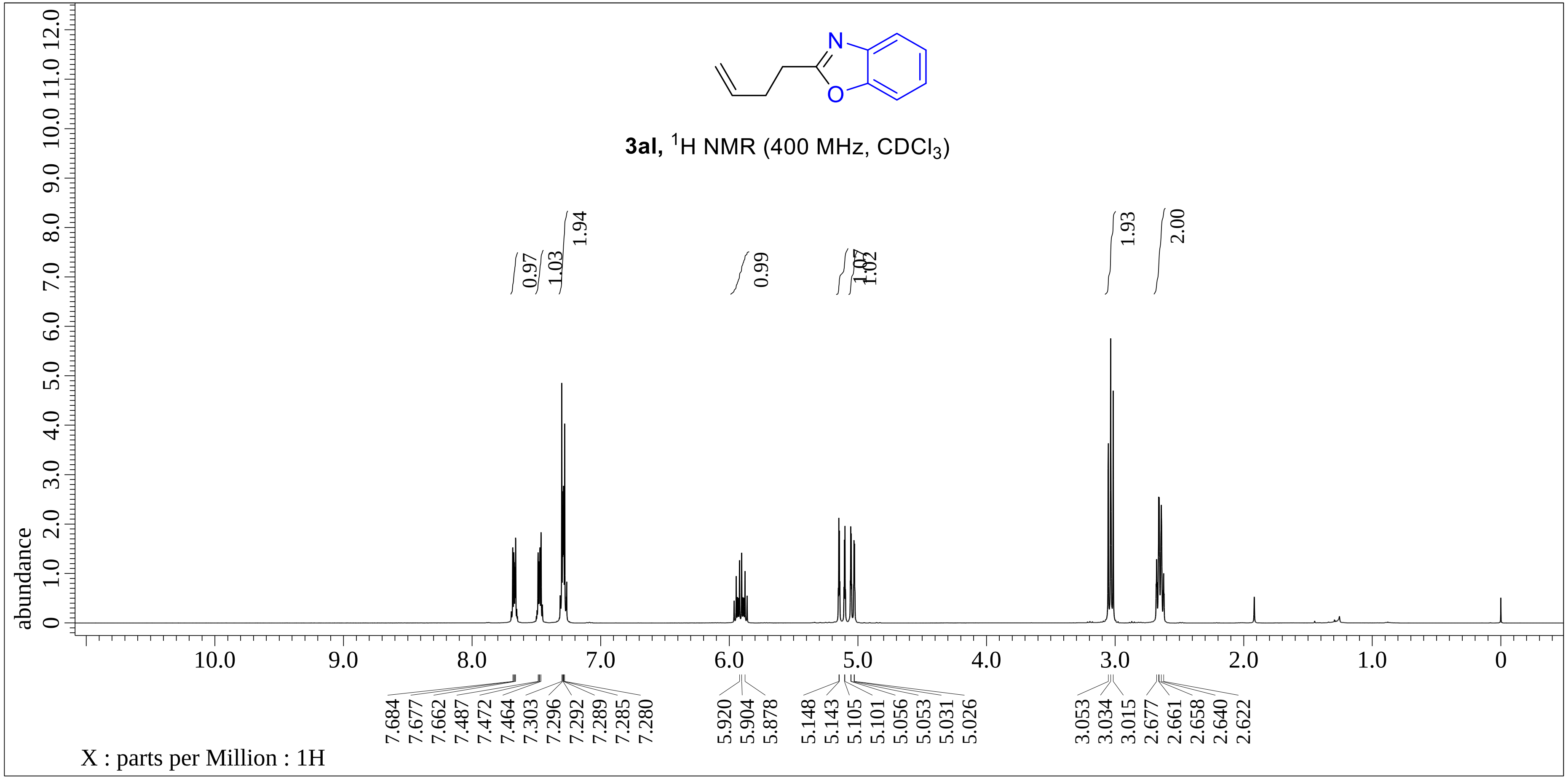

N
O
3al, ^{1}H NMR (400 MHz, $CDCl_3$)
0.97
1.03
1.94
0.99
1.07
1.02
1.93
2.00
abundance
10.0
9.0
8.0
7.0
6.0
5.0
4.0
3.0
2.0
1.0
0
7.684
7.677
7.662
7.487
7.472
7.464
7.303
7.296
7.292
7.289
7.285
7.280
5.920
5.904
5.878
5.148
5.143
5.105
5.101
5.056
5.053
5.031
5.026
3.053
3.034
3.015
2.677
2.661
2.658
2.640
2.622
X : parts per Million : 1H

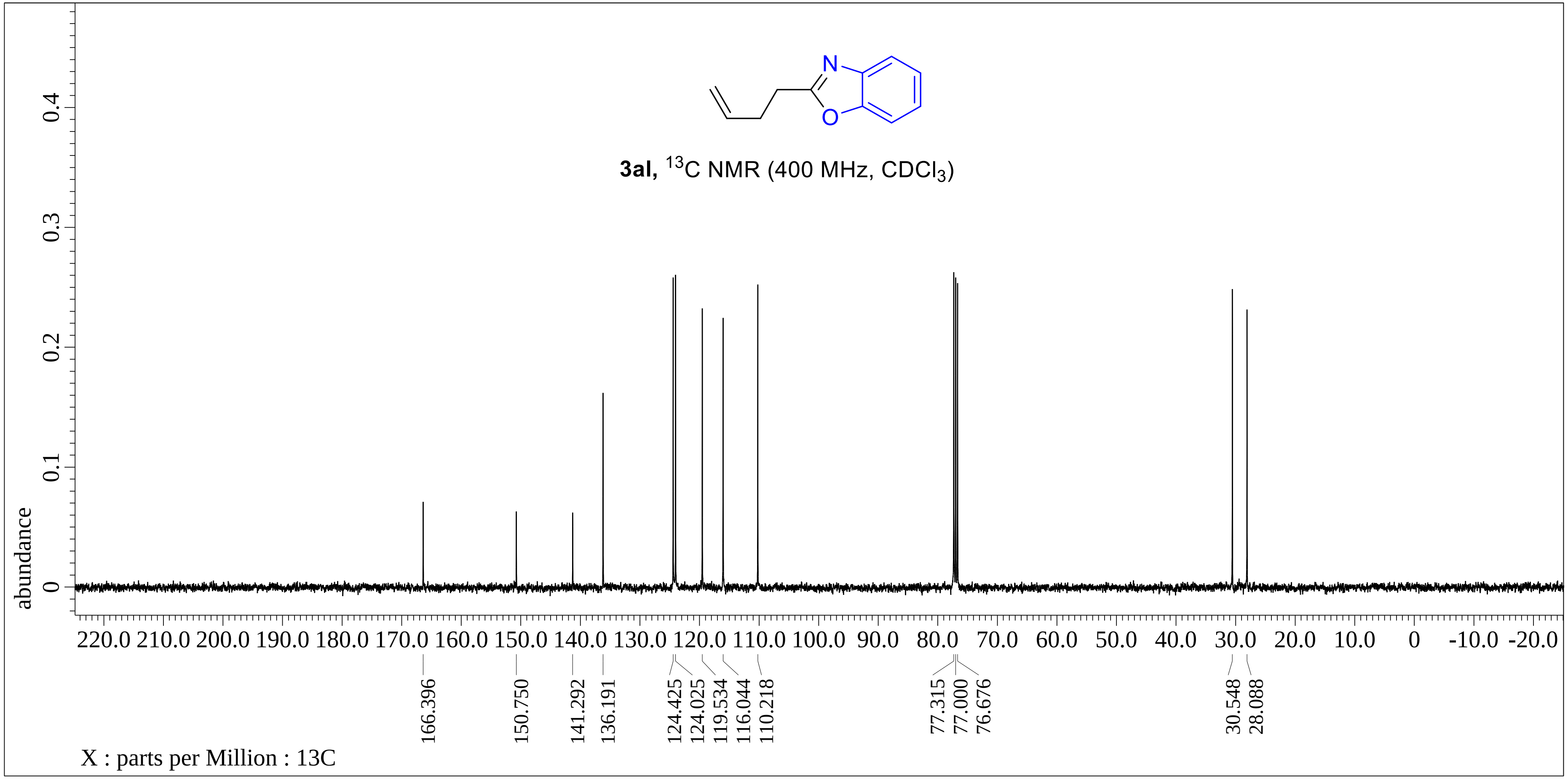
N
O
3al, ^{13}C NMR (400 MHz, $CDCl_3$)
abundance
0.4
0.3
0.2
0.1
0
220.0 210.0 200.0 190.0 180.0 170.0 160.0 150.0 140.0 130.0 120.0 110.0 100.0 90.0 80.0 70.0 60.0 50.0 40.0 30.0 20.0 10.0 0 -10.0 -20.0
166.396
150.750
141.292
136.191
124.425
124.025
119.534
116.044
110.218
77.315
77.000
76.676
30.548
28.088
X : parts per Million : 13C

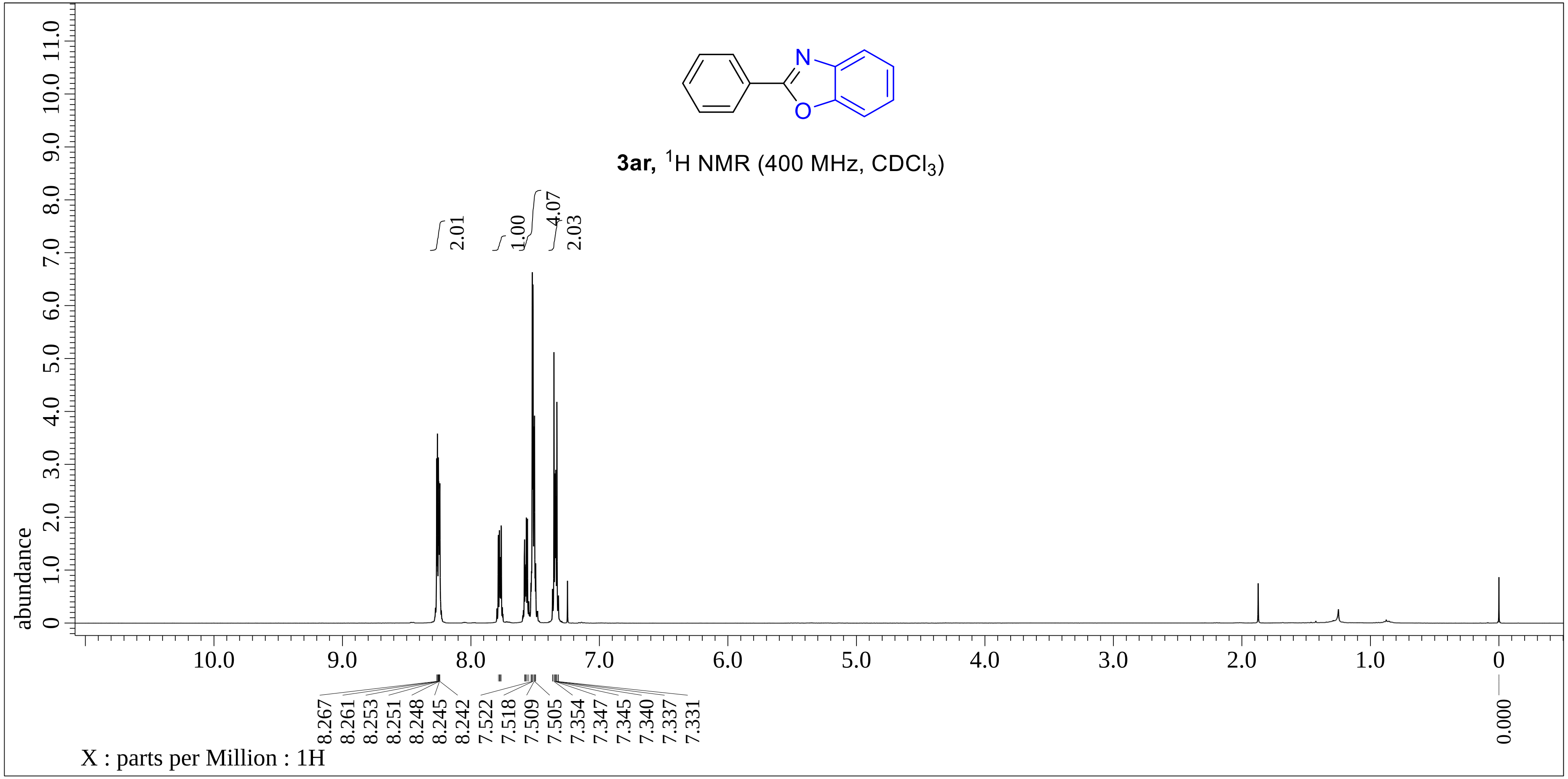

3ar, 1H NMR (400 MHz, CDCl3)
N
O
2.01
1.00
4.07
2.03
abundance
X : parts per Million : 1H
8.267
8.261
8.253
8.251
8.248
8.245
8.242
7.522
7.518
7.509
7.505
7.354
7.347
7.345
7.340
7.337
7.331
0.000

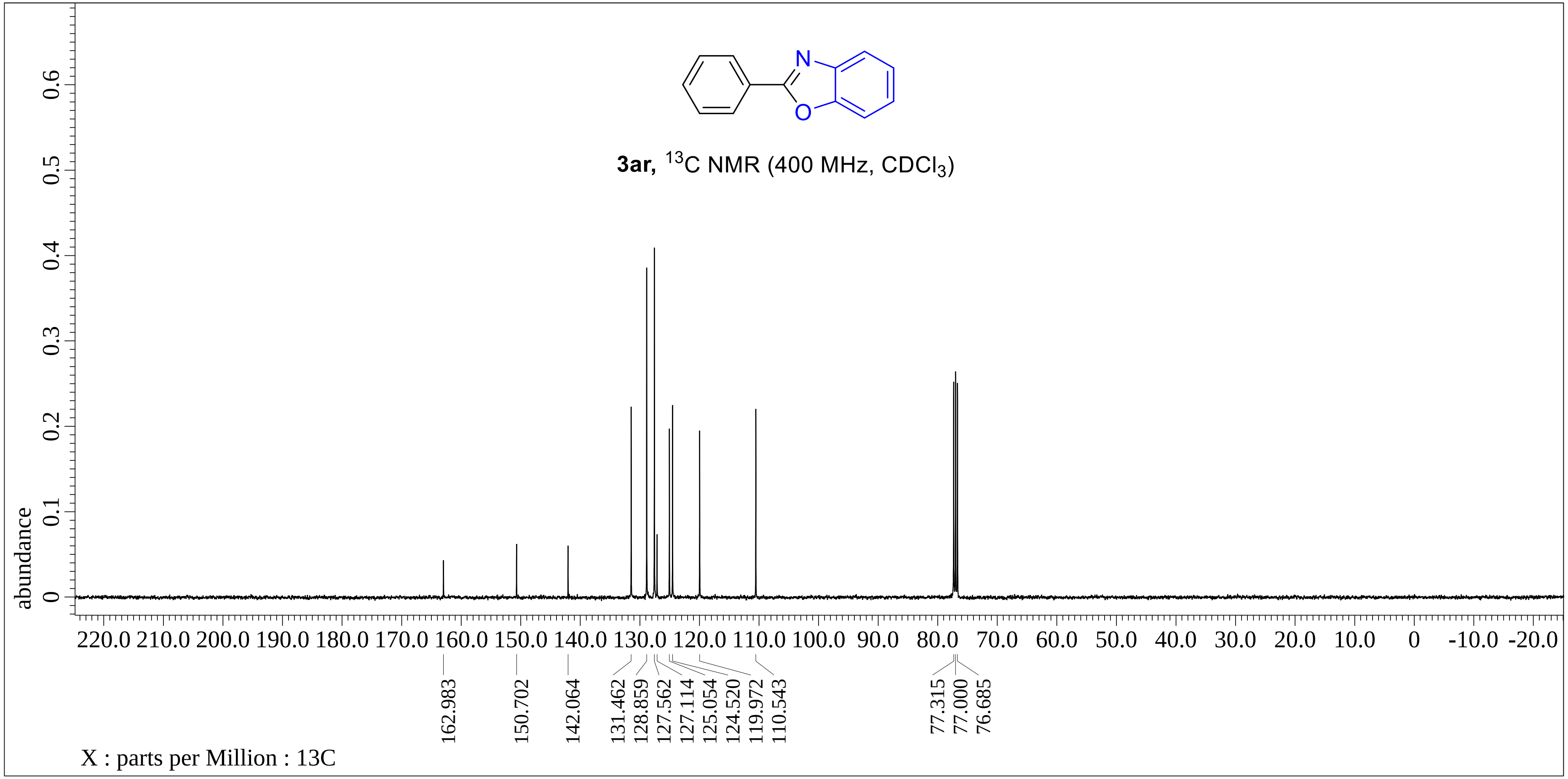

3ar, ^{13}C NMR (400 MHz, $CDCl_3$)
N
O
abundance
0.6
0.5
0.4
0.3
0.2
0.1
0
220.0 210.0 200.0 190.0 180.0 170.0 160.0 150.0 140.0 130.0 120.0 110.0 100.0 90.0 80.0 70.0 60.0 50.0 40.0 30.0 20.0 10.0 0 -10.0 -20.0
162.983
150.702
142.064
131.462
128.859
127.562
127.114
125.054
124.520
119.972
110.543
77.315
77.000
76.685
X : parts per Million : 13C

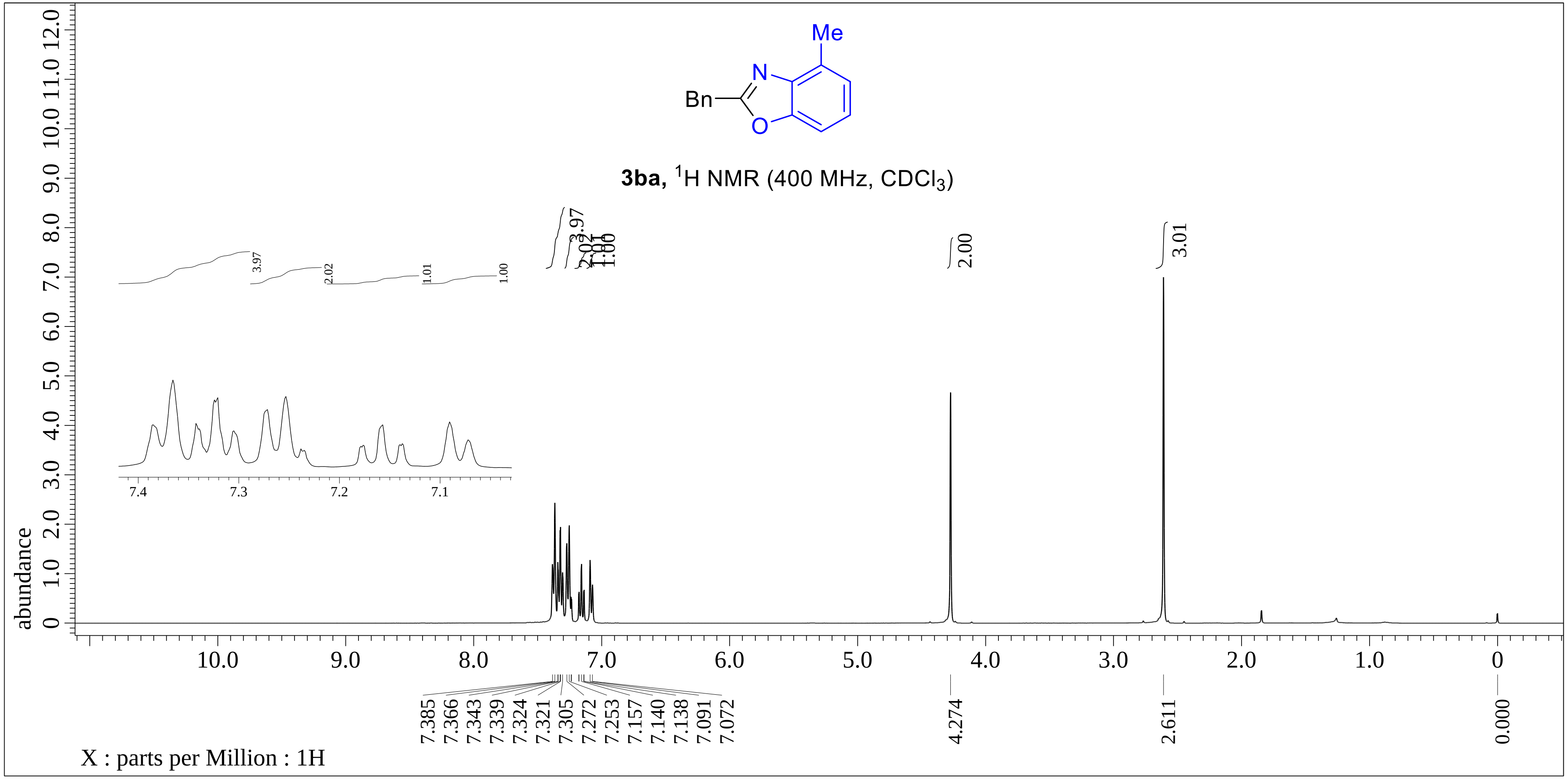
Me
N
Bn
O
3ba, 1H NMR (400 MHz, CDCl3)
abundance
X : parts per Million : 1H
7.385
7.366
7.343
7.339
7.324
7.321
7.305
7.272
7.253
7.157
7.140
7.138
7.091
7.072
4.274
2.611
0.000
3.97
2.02
1.01
1.00
2.00
3.01

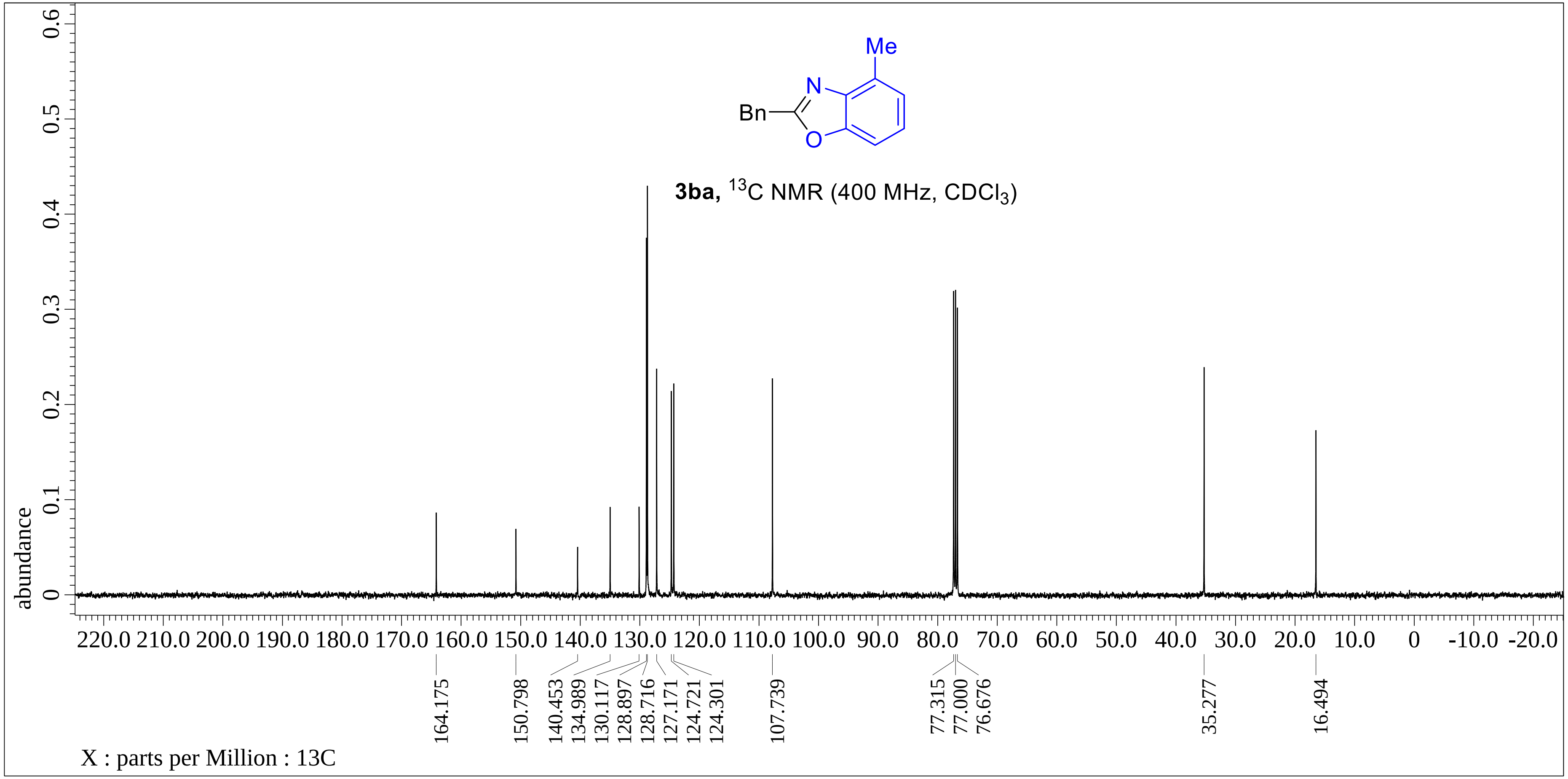
Me
N
Bn
O
3ba, ^{13}C NMR (400 MHz, $CDCl_3$)
abundance
0.6
0.5
0.4
0.3
0.2
0.1
0
220.0 210.0 200.0 190.0 180.0 170.0 160.0 150.0 140.0 130.0 120.0 110.0 100.0 90.0 80.0 70.0 60.0 50.0 40.0 30.0 20.0 10.0 0 -10.0 -20.0
164.175
150.798
140.453
134.989
130.117
128.897
128.716
127.171
124.721
124.301
107.739
77.315
77.000
76.676
35.277
16.494
X : parts per Million : 13C

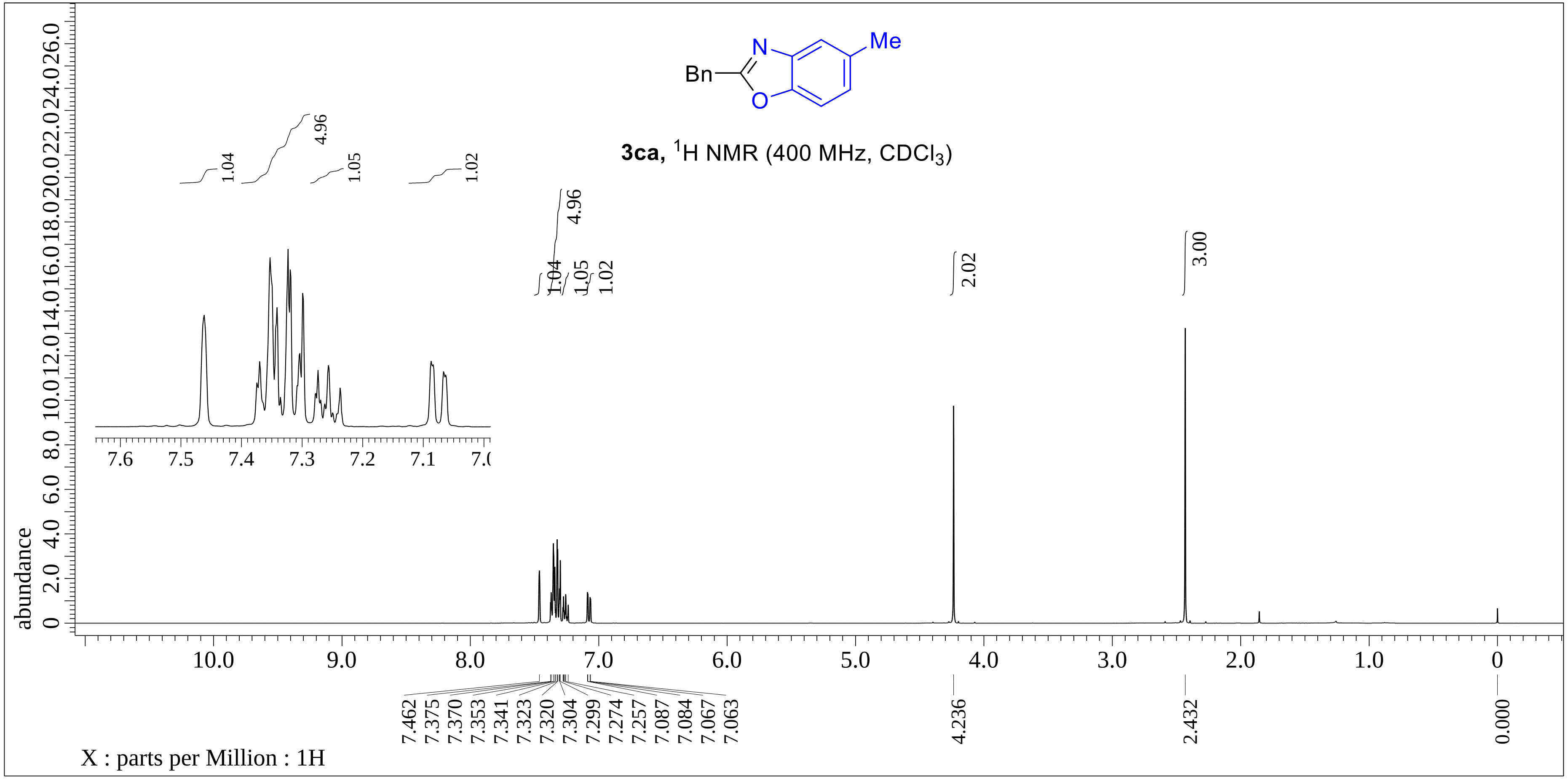

Bn
N
O
Me
3ca, 1H NMR (400 MHz, CDCl3)
abundance
X : parts per Million : 1H
7.462
7.375
7.370
7.353
7.341
7.323
7.320
7.304
7.299
7.274
7.257
7.087
7.084
7.067
7.063
4.236
2.432
0.000
1.04
4.96
1.05
1.02
2.02
3.00

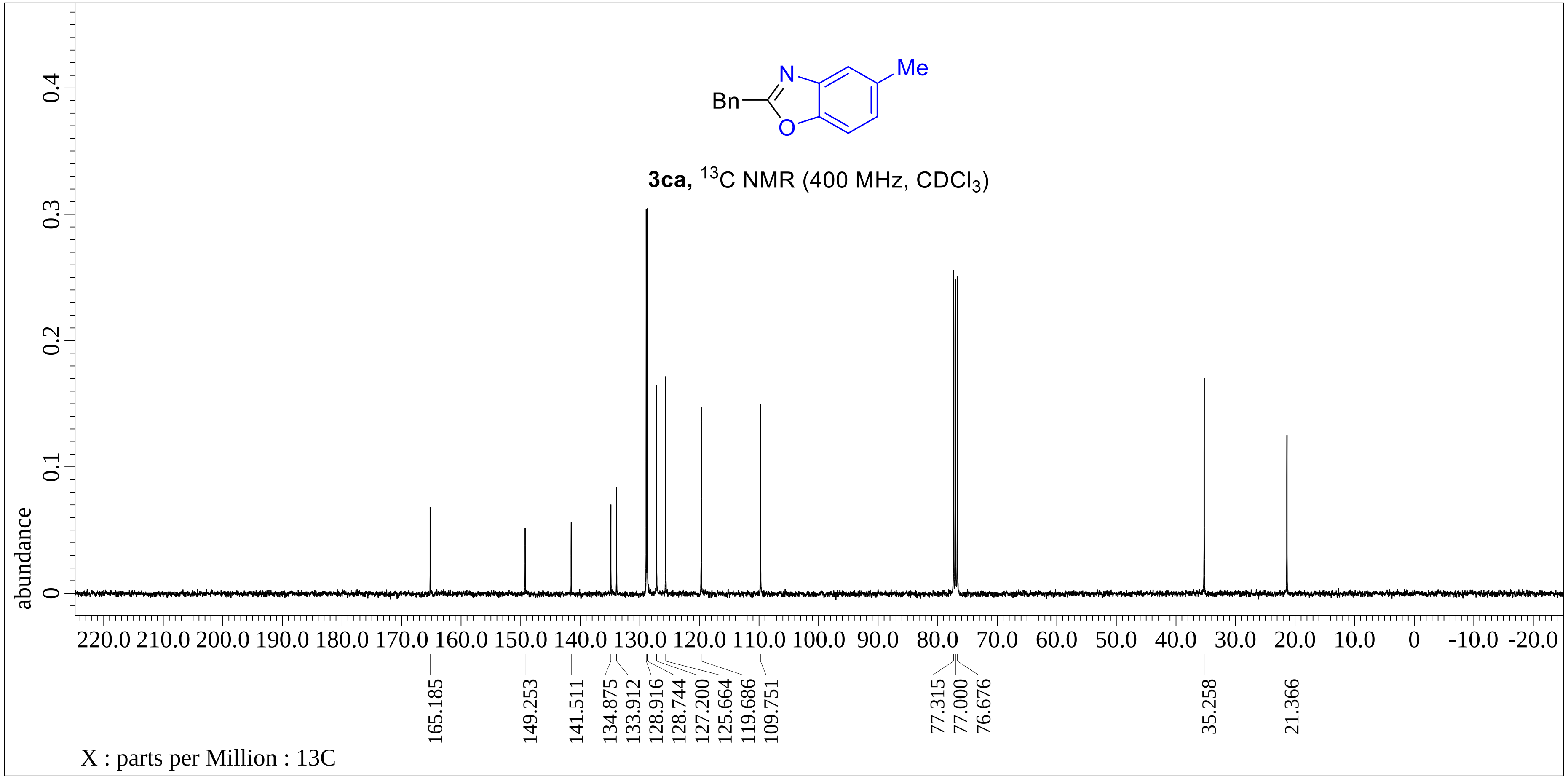
Bn
N
O
Me
3ca, 13C NMR (400 MHz, CDCl3)
abundance
0.4
0.3
0.2
0.1
0
220.0 210.0 200.0 190.0 180.0 170.0 160.0 150.0 140.0 130.0 120.0 110.0 100.0 90.0 80.0 70.0 60.0 50.0 40.0 30.0 20.0 10.0 0 -10.0 -20.0
165.185
149.253
141.511
134.875
133.912
128.916
128.744
127.200
125.664
119.686
109.751
77.315
77.000
76.676
35.258
21.366
X : parts per Million : 13C

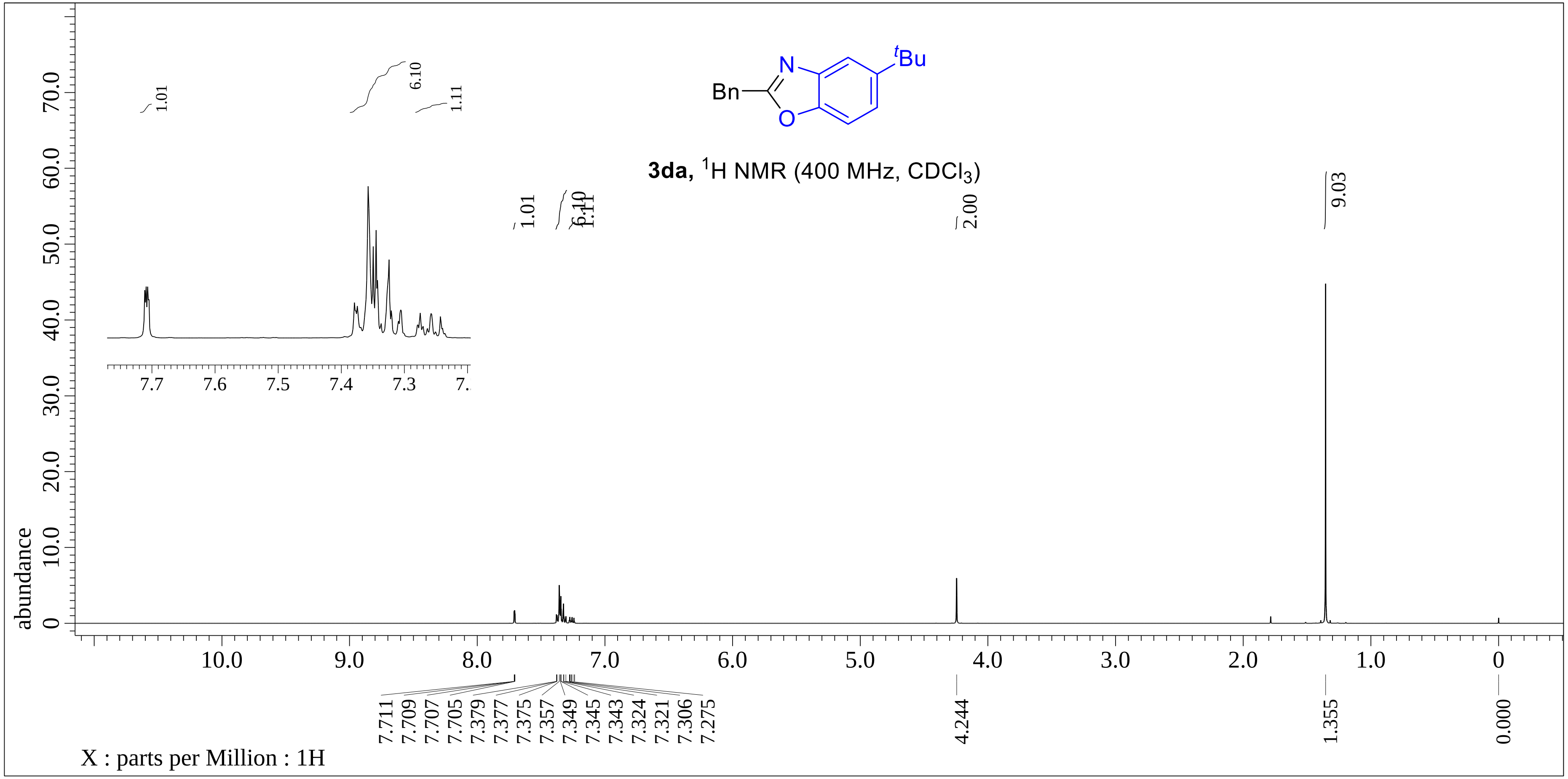
Bn
N
O
tBu
3da, 1H NMR (400 MHz, CDCl3)
abundance
X : parts per Million : 1H
1.01
6.10
1.11
2.00
9.03
7.711
7.709
7.707
7.705
7.379
7.377
7.375
7.357
7.349
7.345
7.343
7.324
7.321
7.306
7.275
4.244
1.355
0.000

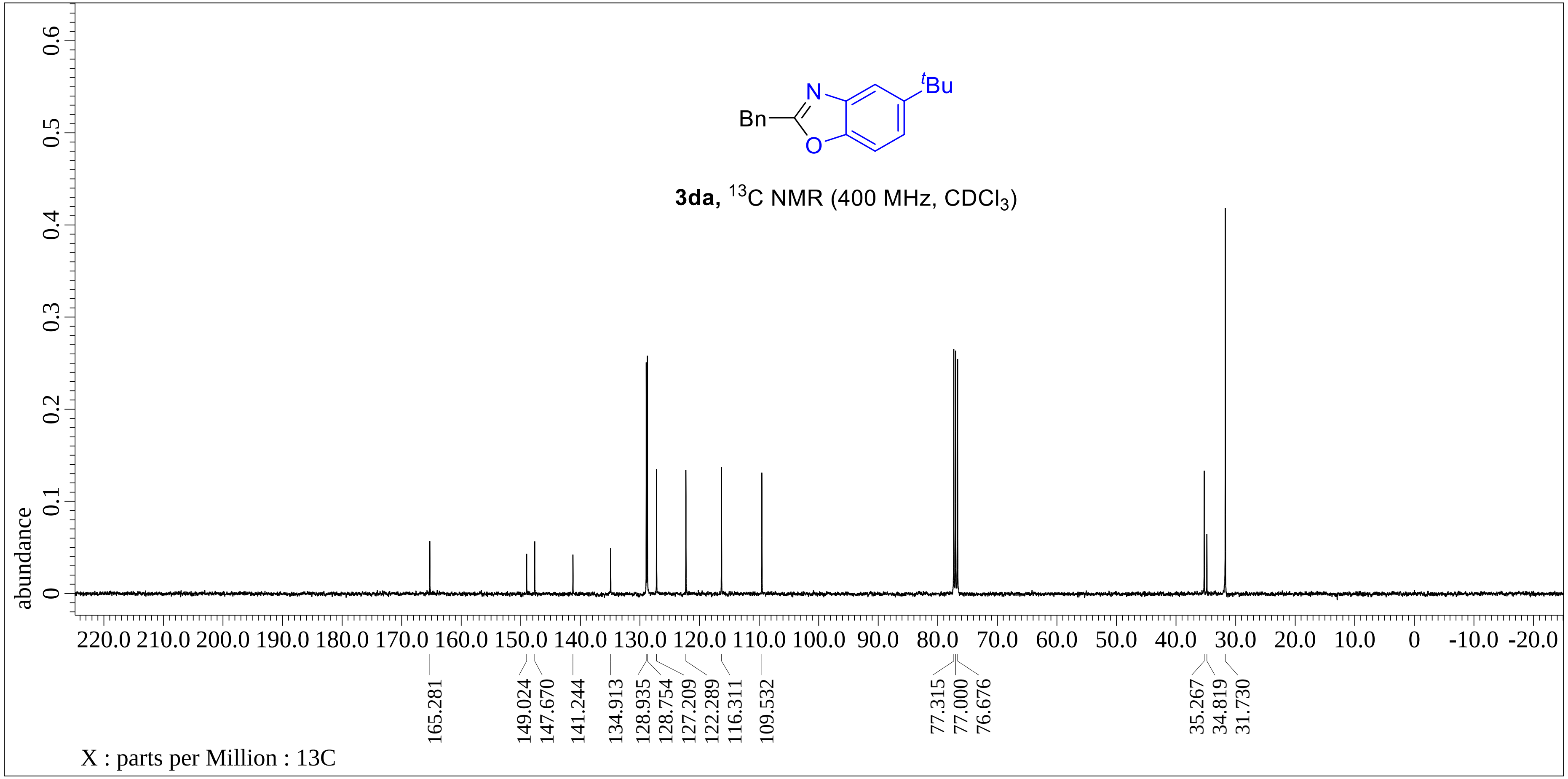

Bn
N
O
tBu
3da, 13C NMR (400 MHz, CDCl3)
abundance
0
0.1
0.2
0.3
0.4
0.5
0.6
220.0 210.0 200.0 190.0 180.0 170.0 160.0 150.0 140.0 130.0 120.0 110.0 100.0 90.0 80.0 70.0 60.0 50.0 40.0 30.0 20.0 10.0 0 -10.0 -20.0
165.281
149.024
147.670
141.244
134.913
128.935
128.754
127.209
122.289
116.311
109.532
77.315
77.000
76.676
35.267
34.819
31.730
X : parts per Million : 13C

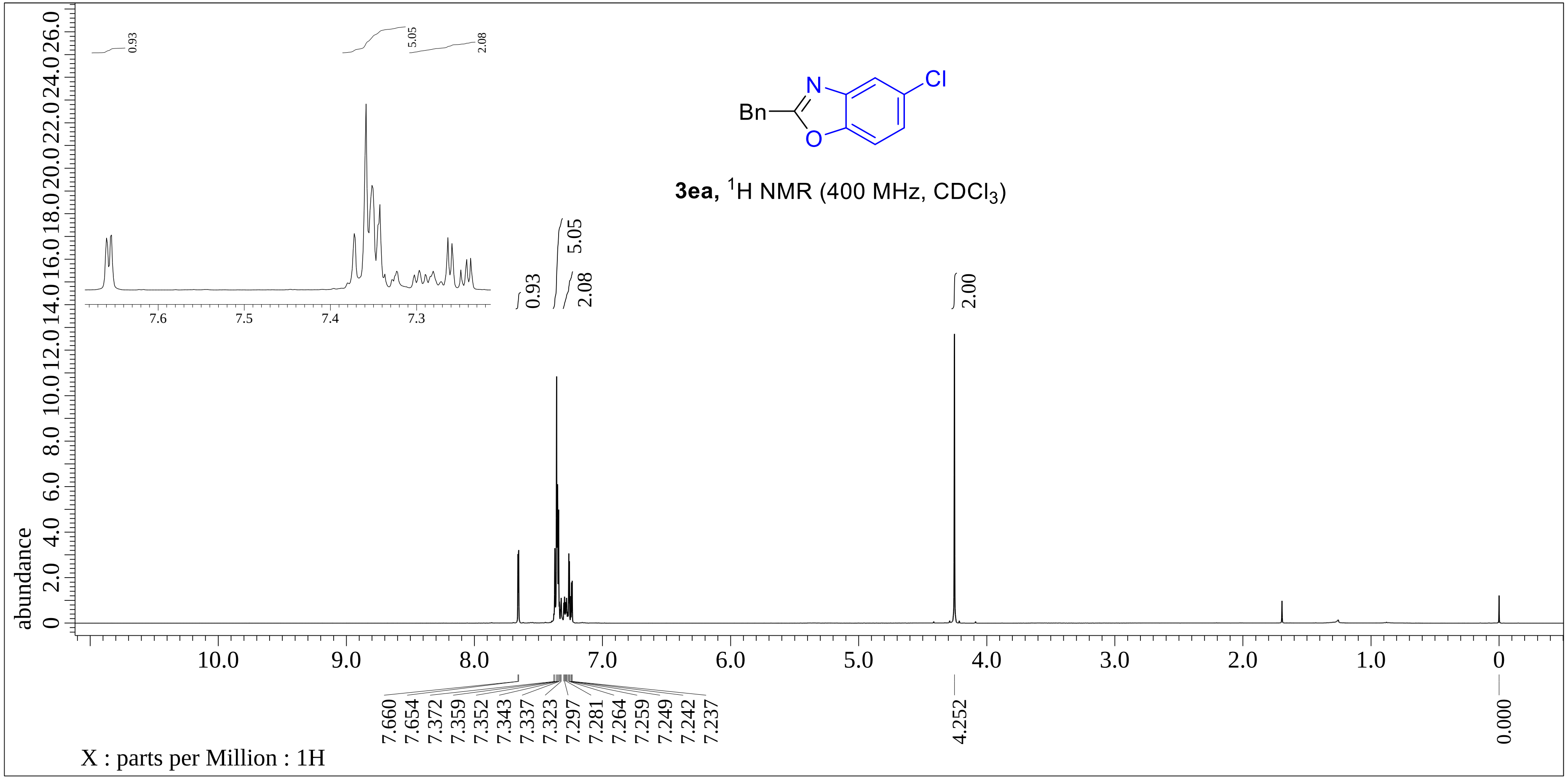

Bn
N
O
Cl
3ea, 1H NMR (400 MHz, CDCl3)
abundance
X : parts per Million : 1H
0.93
5.05
2.08
2.00
7.660
7.654
7.372
7.359
7.352
7.343
7.337
7.323
7.297
7.281
7.264
7.259
7.249
7.242
7.237
4.252
0.000

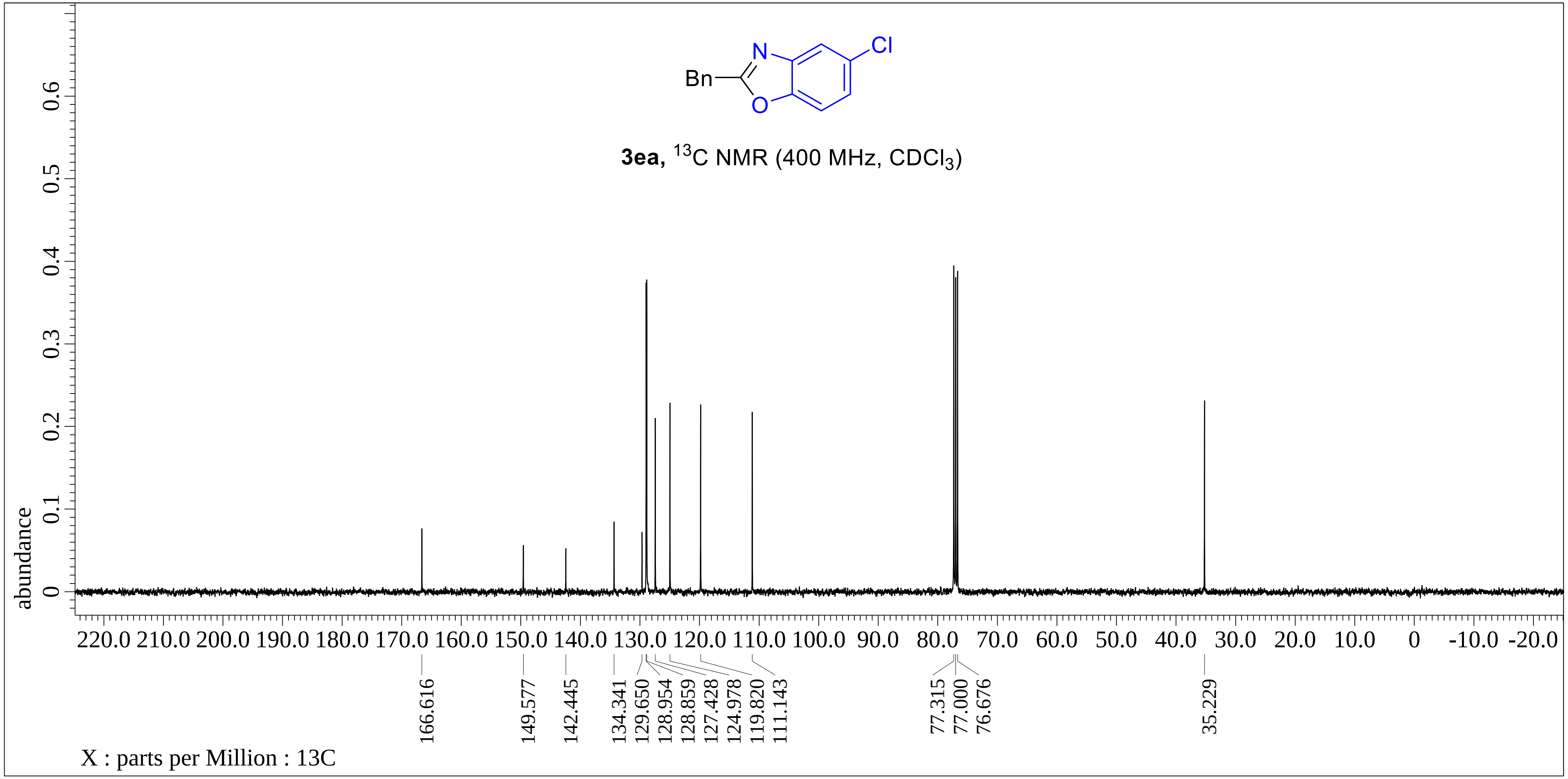

Cl
N
Bn
O
3ea, 13C NMR (400 MHz, CDCl3)
abundance
0
0.1
0.2
0.3
0.4
0.5
0.6
220.0 210.0 200.0 190.0 180.0 170.0 160.0 150.0 140.0 130.0 120.0 110.0 100.0 90.0 80.0 70.0 60.0 50.0 40.0 30.0 20.0 10.0 0 -10.0 -20.0
166.616
149.577
142.445
134.341
129.650
128.954
128.859
127.428
124.978
119.820
111.143
77.315
77.000
76.676
35.229
X : parts per Million : 13C

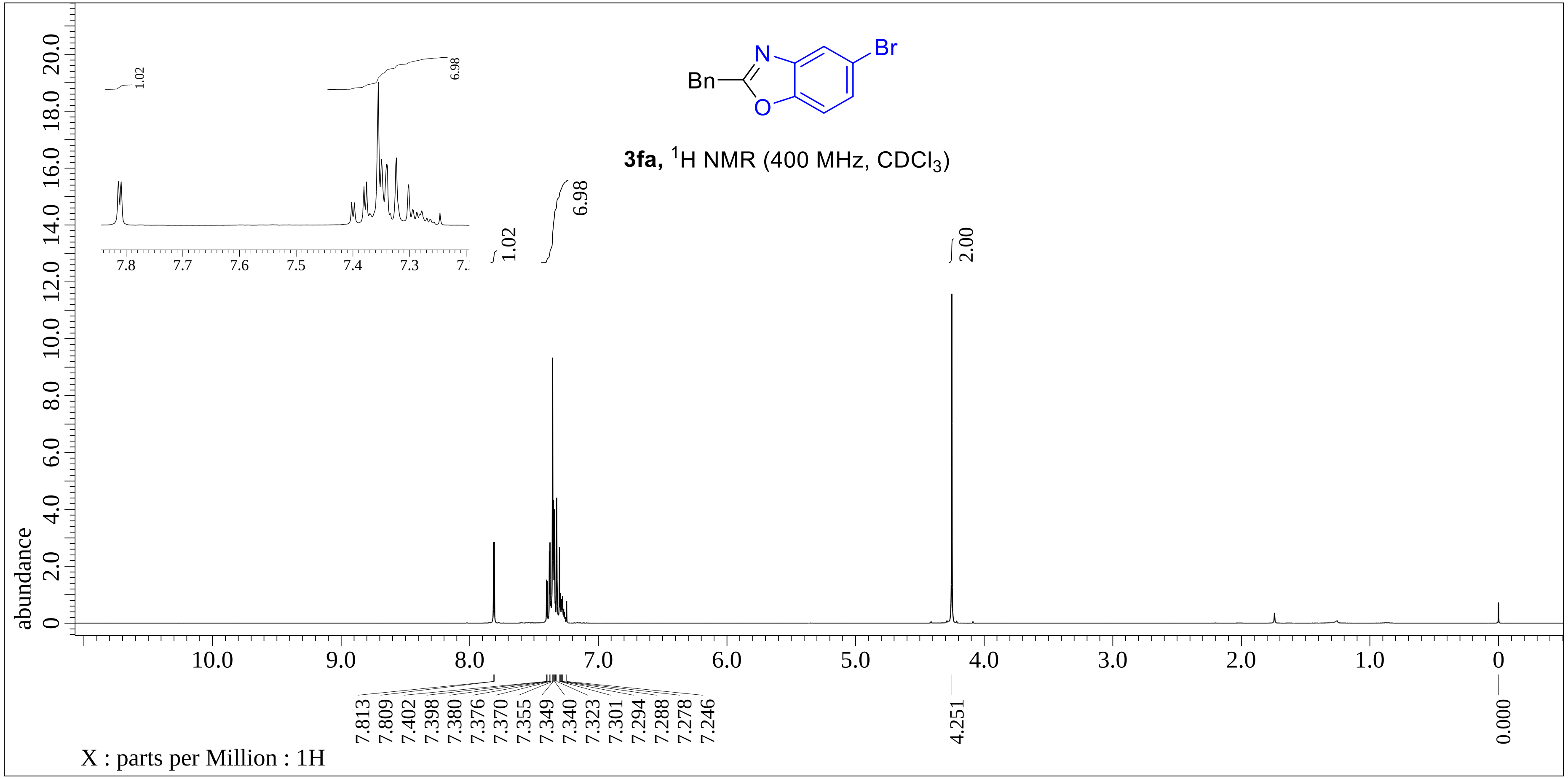

Bn
N
O
Br
3fa, 1H NMR (400 MHz, CDCl3)
1.02
6.98
2.00
7.813
7.809
7.402
7.398
7.380
7.376
7.370
7.355
7.349
7.340
7.323
7.301
7.294
7.288
7.278
7.246
4.251
0.000
abundance
X : parts per Million : 1H

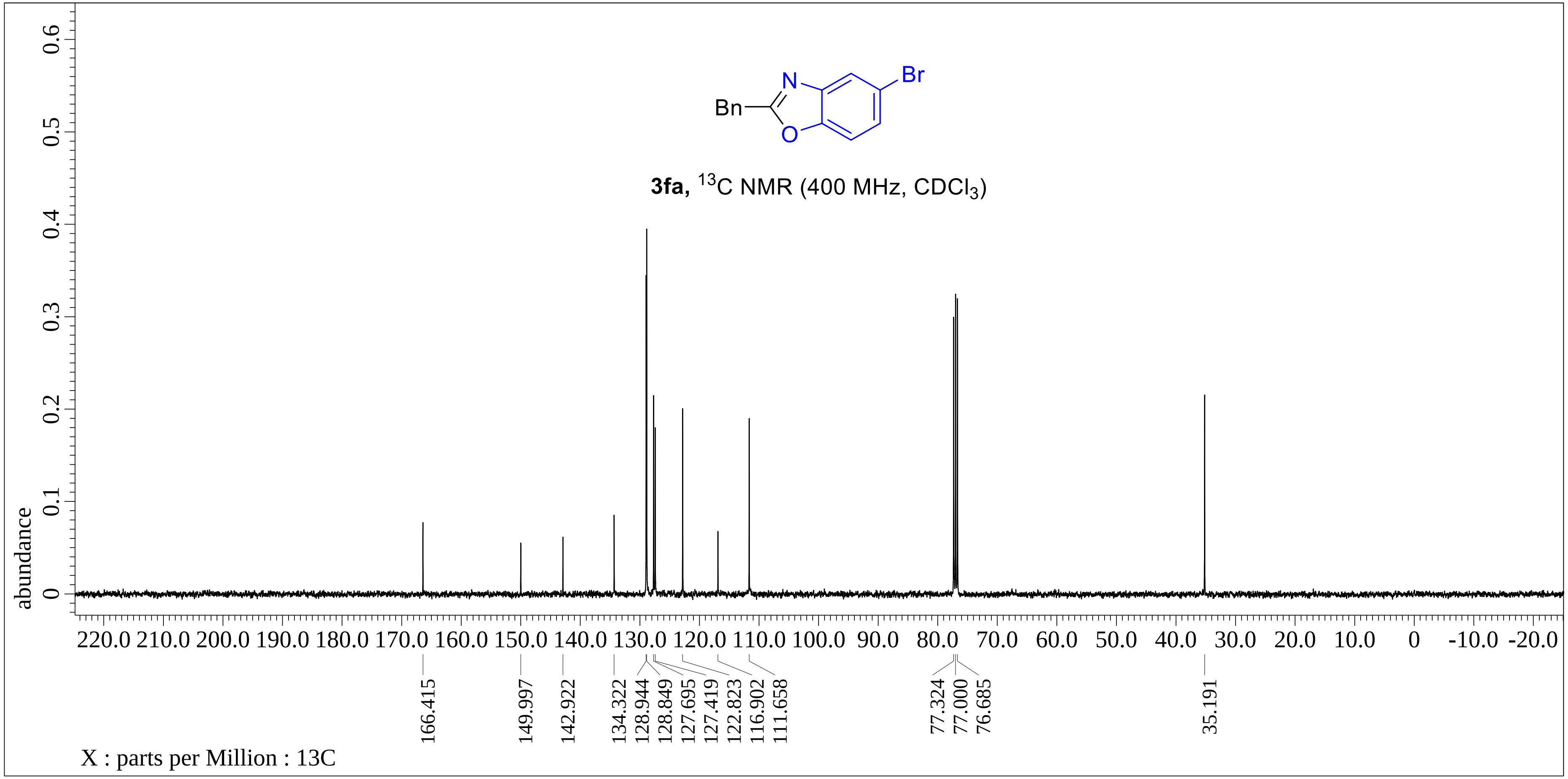

Bn
N
O
Br
3fa, ¹³C NMR (400 MHz, CDCl3)
abundance
0
0.1
0.2
0.3
0.4
0.5
0.6
220.0 210.0 200.0 190.0 180.0 170.0 160.0 150.0 140.0 130.0 120.0 110.0 100.0 90.0 80.0 70.0 60.0 50.0 40.0 30.0 20.0 10.0 0 -10.0 -20.0
166.415
149.997
142.922
134.322
128.944
128.849
127.695
127.419
122.823
116.902
111.658
77.324
77.000
76.685
35.191
X : parts per Million : 13C

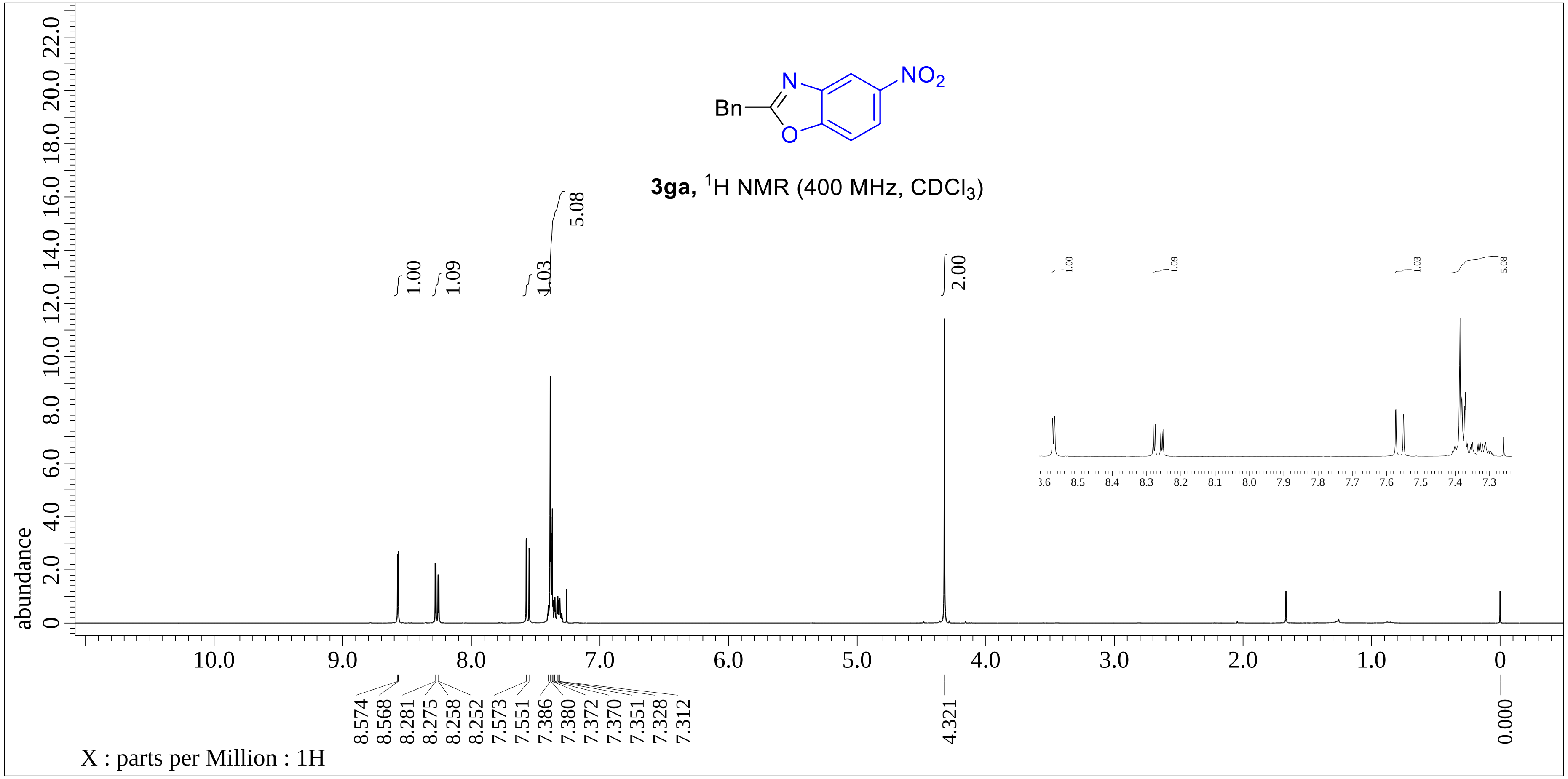

Bn
N
O
NO2
3ga, 1H NMR (400 MHz, CDCl3)
abundance
X : parts per Million : 1H
1.00
1.09
1.03
5.08
2.00
8.574
8.568
8.281
8.275
8.258
8.252
7.573
7.551
7.386
7.380
7.372
7.370
7.351
7.328
7.312
4.321
0.000

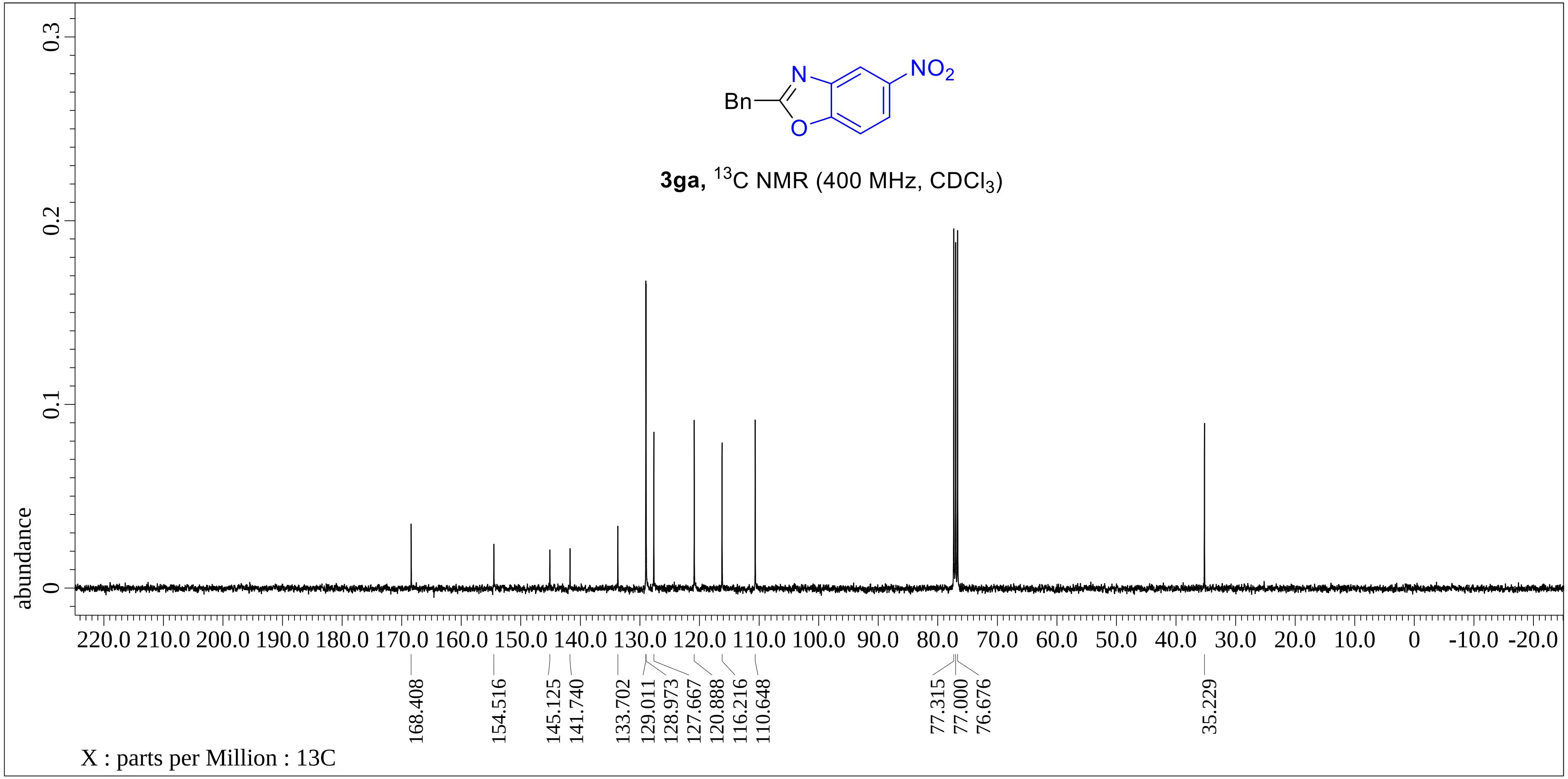

Bn
N
O
NO2
3ga, 13C NMR (400 MHz, CDCl3)
abundance
0.3
0.2
0.1
0
220.0 210.0 200.0 190.0 180.0 170.0 160.0 150.0 140.0 130.0 120.0 110.0 100.0 90.0 80.0 70.0 60.0 50.0 40.0 30.0 20.0 10.0 0 -10.0 -20.0
168.408
154.516
145.125
141.740
133.702
129.011
128.973
127.667
120.888
116.216
110.648
77.315
77.000
76.676
35.229
X : parts per Million : 13C

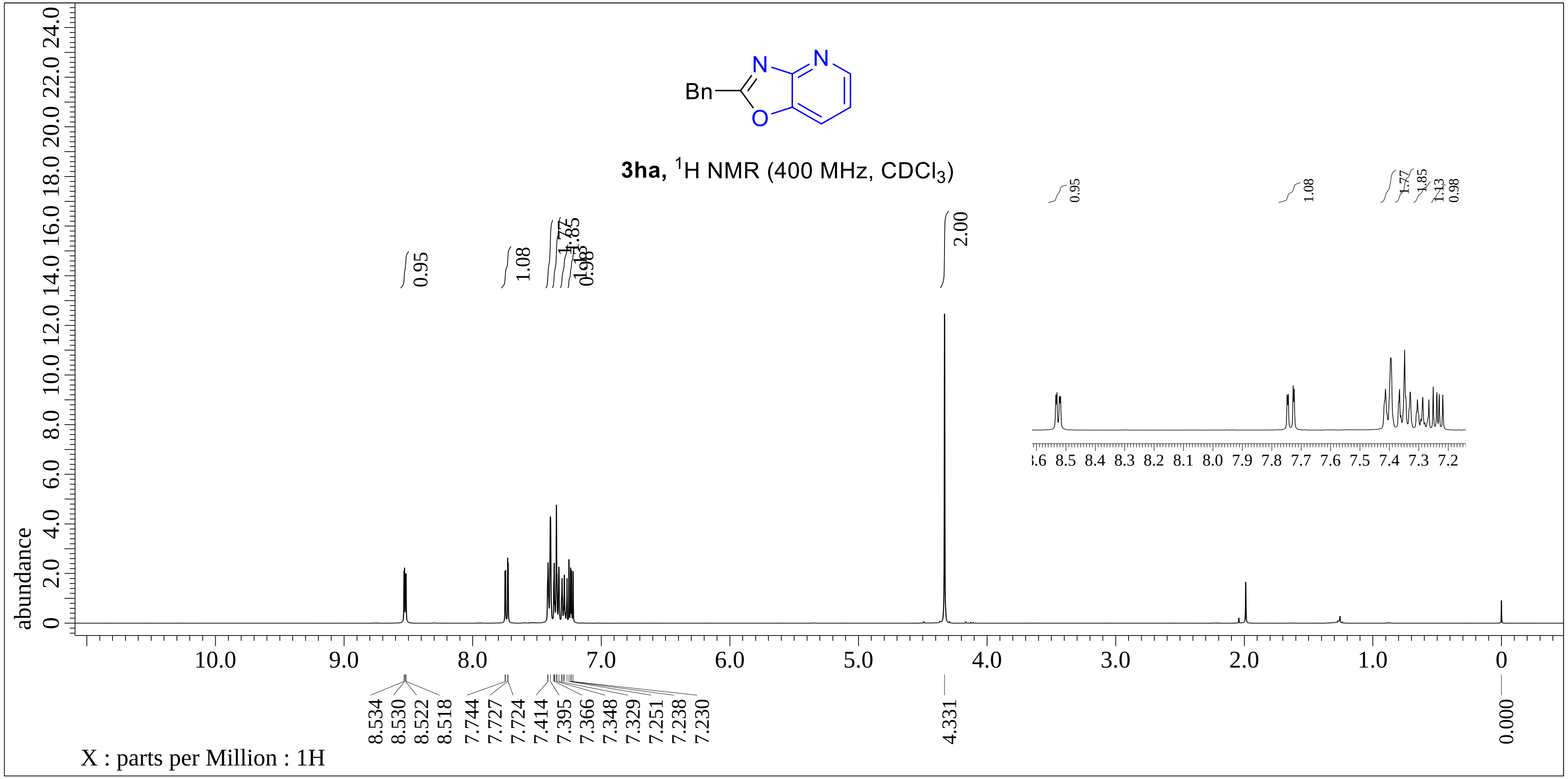

Bn
N
N
O
3ha, 1H NMR (400 MHz, CDCl3)
0.95
1.08
2.00
abundance
X : parts per Million : 1H
8.534
8.530
8.522
8.518
7.744
7.727
7.724
7.414
7.395
7.366
7.348
7.329
7.251
7.238
7.230
4.331
0.000

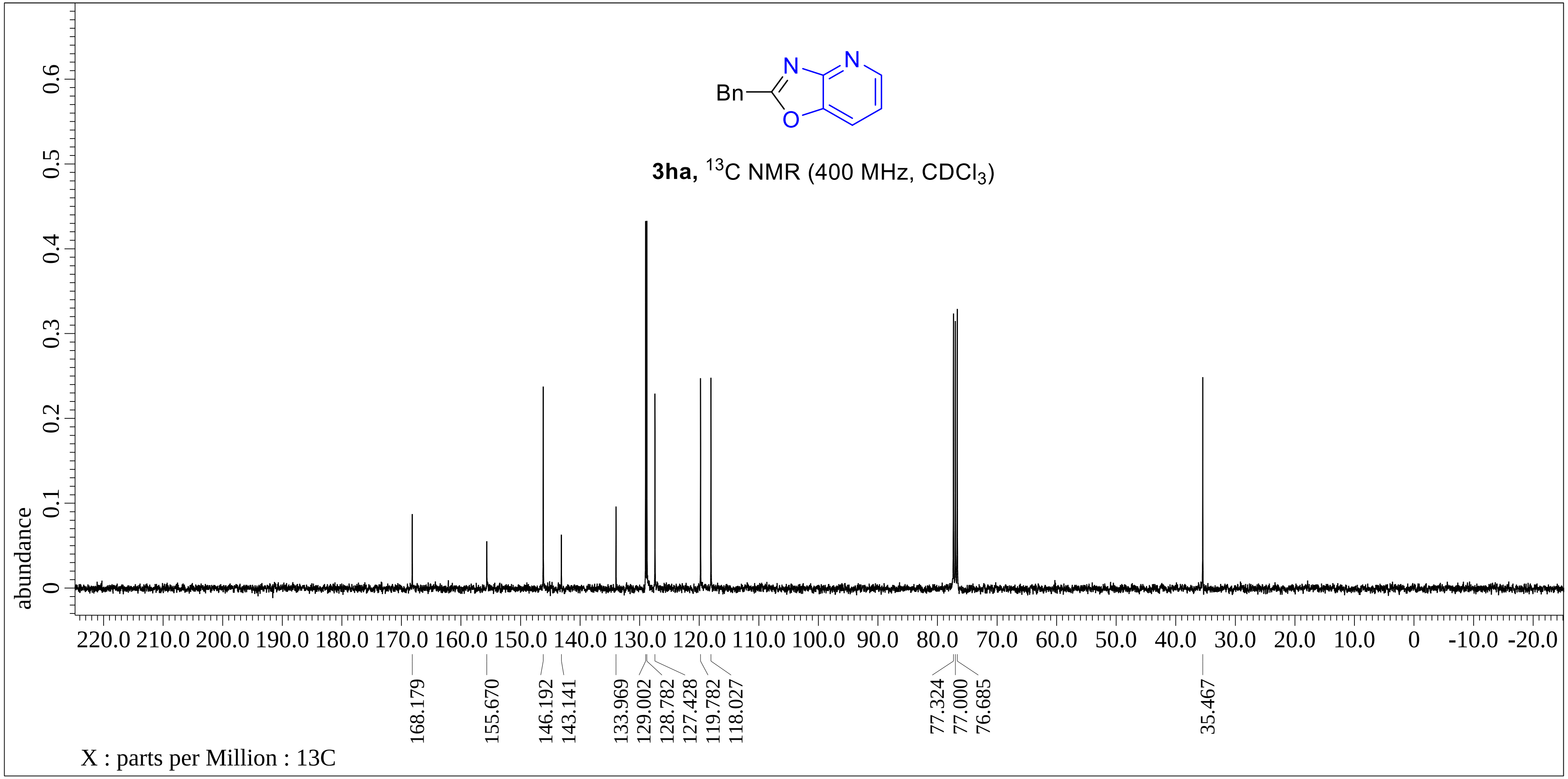

Bn
N
N
O
3ha, 13C NMR (400 MHz, CDCl3)
abundance
0.6
0.5
0.4
0.3
0.2
0.1
0
220.0 210.0 200.0 190.0 180.0 170.0 160.0 150.0 140.0 130.0 120.0 110.0 100.0 90.0 80.0 70.0 60.0 50.0 40.0 30.0 20.0 10.0 0 -10.0 -20.0
168.179
155.670
146.192
143.141
133.969
129.002
128.782
127.428
119.782
118.027
77.324
77.000
76.685
35.467
X : parts per Million : 13C

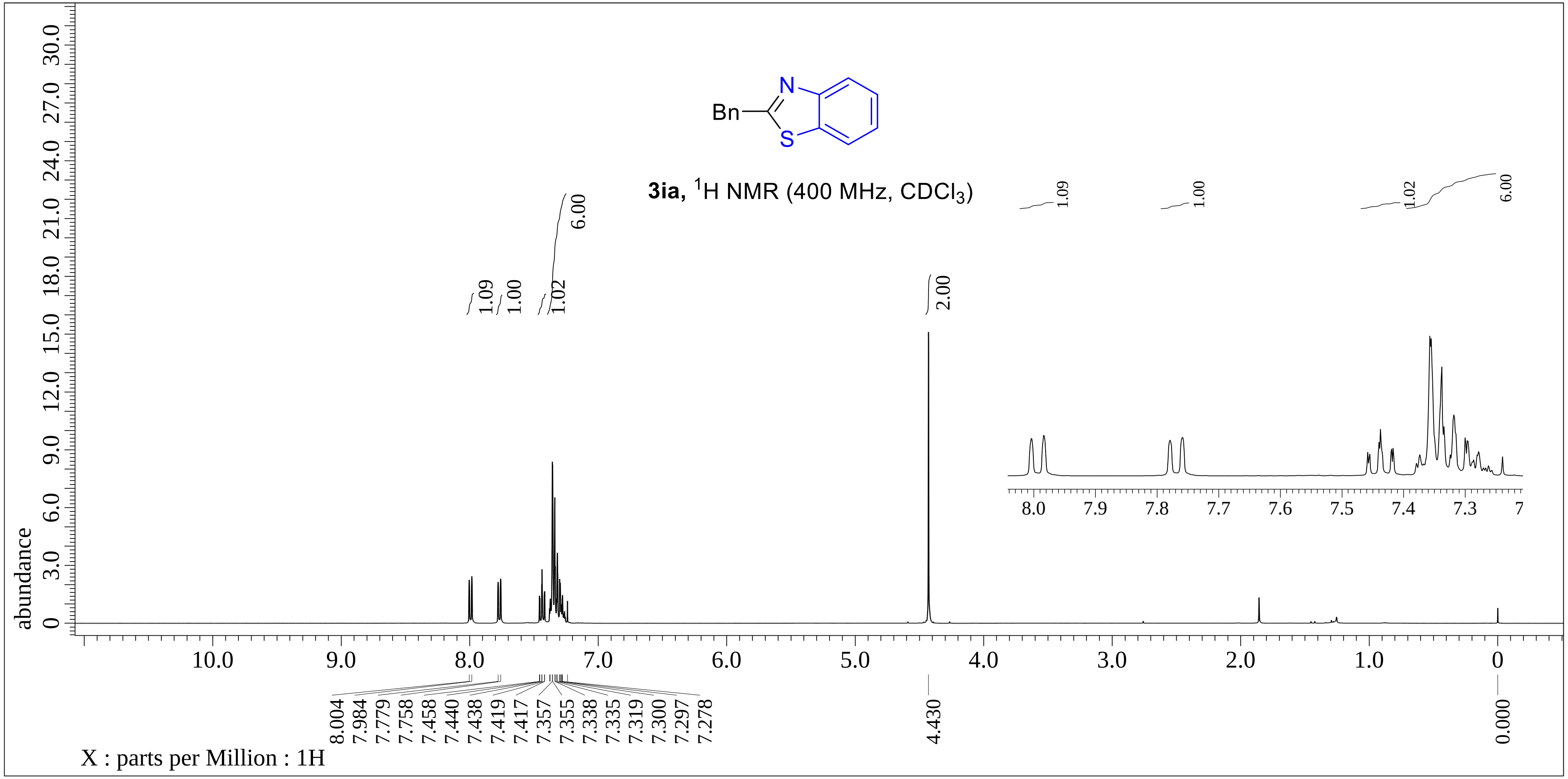
3ia, ^{1}H NMR (400 MHz, $CDCl_3$)
Bn
N
S
abundance
X : parts per Million : 1H
1.09
1.00
1.02
6.00
2.00
8.004
7.984
7.779
7.758
7.458
7.440
7.438
7.419
7.417
7.357
7.355
7.338
7.335
7.319
7.300
7.297
7.278
4.430
0.000

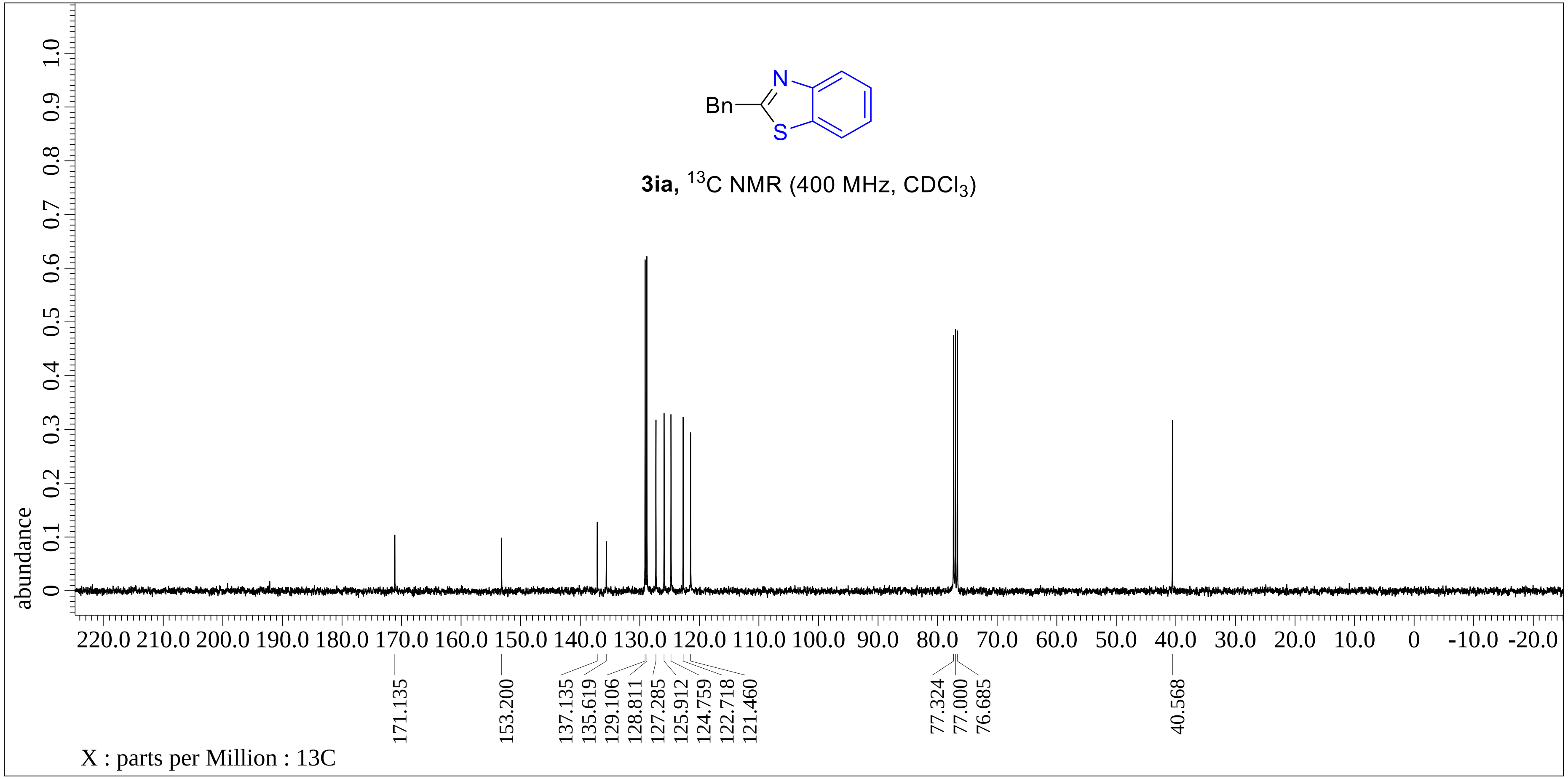

Bn
N
S
3ia, ^{13}C NMR (400 MHz, CDCl$_3$)
abundance
0 0.1 0.2 0.3 0.4 0.5 0.6 0.7 0.8 0.9 1.0
220.0 210.0 200.0 190.0 180.0 170.0 160.0 150.0 140.0 130.0 120.0 110.0 100.0 90.0 80.0 70.0 60.0 50.0 40.0 30.0 20.0 10.0 0 -10.0 -20.0
171.135
153.200
137.135
135.619
129.106
128.811
127.285
125.912
124.759
122.718
121.460
77.324
77.000
76.685
40.568
X : parts per Million : 13C
